# Advances in Diclofenac Derivatives: Exploring Carborane‐Substituted *N*‐Methyl and Nitrile Analogs for Anticancer Therapy

**DOI:** 10.1002/cmdc.202500084

**Published:** 2025-04-14

**Authors:** Christoph Selg, Robert Schuster, Aleksandr Kazimir, Peter Lönnecke, Mara Wolniewicz, Jonas Schädlich, Markus Laube, Jens Pietzsch, Vuk Gordić, Tamara Krajnović, Sanja Mijatović, Danijela Maksimović‐Ivanić, Evamarie Hey‐Hawkins

**Affiliations:** ^1^ Faculty of Chemistry and Mineralogy Institute of Bioanalytical Chemistry Leipzig University Deutscher Platz 5 04103 Leipzig Germany; ^2^ Institute for Drug Discovery Leipzig University Brüderstraße 34 04103 Leipzig Germany; ^3^ Faculty of Chemistry and Mineralogy Institute of Organic Chemistry Leipzig University Johannisallee 29 04103 Leipzig Germany; ^4^ Department of Radiopharmaceutical and Chemical Biology Institute of Radiopharmaceutical Cancer Research Helmholtz‐Zentrum Dresden‐Rossendorf (HZDR) Bautzner Landstraße 400 01328 Dresden Germany; ^5^ Faculty of Chemistry and Food Chemistry School of Science Technische Universität Dresden Mommsenstraße 4 01069 Dresden Germany; ^6^ Department of Immunology Institute for Biological Research “Siniša Stanković” National Institute of the Republic of Serbia University of Belgrade 11108 Belgrade Serbia; ^7^ Department of Chemistry Babeş‐Bolyai University Str. Arany Janos Nr. 11 RO‐400028 Cluj‐Napoca Romania

**Keywords:** aryl mimetic replacements, cancers, carboranes, cyclooxygenases, drug repurposing

## Abstract

This study explores the anticancer potential of *N*‐methylated open‐ring derivatives of carborane‐substituted diclofenac analogs. By *N*‐methylation, the open‐chain form could be trapped and cyclization back to lactam or amidine derivatives is inhibited. A small library of carborane‐ and phenyl‐based secondary and tertiary arylamines bearing carboxylic acid or nitrile groups is synthesized and analyzed for their COX affinity in vitro and in silico. The compounds are further evaluated against mouse adenocarcinoma (MC38), human colorectal carcinoma (HCT116), and human colorectal adenocarcinoma (HT29) cell lines and show potent cytotoxicity. Additional biological assessments of the mode of action are performed using flow cytometric techniques and fluorescence microscopy. The data obtained reveal a common antiproliferative effect coupled with the induction of caspase‐independent apoptosis and the specific effects of the compound on the phenotype of MC38 cells, resulting in impaired cell viability of MC38 cells and satisfactory selectivity exceeding the antitumor activity of diclofenac.

## Graphical abstract

By *N*‐methylation, the intramolecular lactam formation in carborane‐substituted diclofenac analogs is prevented, yielding a series of open‐chain derivatives alongside with their phenyl analogs exhibiting anticancer activity and thus offering a promising avenue for future drug development.

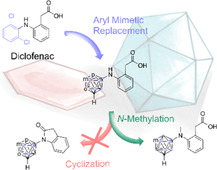

## 1 Introduction

The exploration of already established drugs in further therapeutic targets is a cost‐ and time‐efficient strategy in drug discovery as several steps of the development can be shortened.^[^ ^1^ ^]^ Especially in oncology, the repurposing of drugs is widely applied and has spawned numerous new anticancer applications.^[^ ^2^ ^]^ Among these, the repurposing of diclofenac and more recently, modified derivatives of diclofenac have caught our attention.^[^ ^3^ ^]^ Extensive literature as well as clinical trials support the efficacy of diclofenac and its derivatives in vitro against various cancer cell lines and in vivo.^[^ ^4^, ^5^, ^6^ ^]^ The inhibition of cyclooxygenase enzymes (COX‐1 and particularly COX‐2) plays a crucial role in inducing apoptosis by disrupting prostaglandin synthesis, thereby removing the antiapoptotic influence of prostaglandins such as PGE_2_.^[^ ^7^ ^]^ This inhibition also suppresses the inflammatory microenvironment conducive to tumorigenesis.^[^ ^3^, ^5^, ^6^, ^8^, ^9^ ^]^ Beyond COX‐dependent pathways, diclofenac was also shown to induce apoptosis through COX‐independent effects.^[^ ^10^ ^]^

These mechanisms involve the regulation of crucial cellular processes that affect cancer cell survival and proliferation. One such pathway includes the downregulation of Myc gene expression and glycolysis, alongside the inhibition of cellular lactate efflux.^[^ ^11^ ^]^ This reduction in lactate efflux decreases the availability of crucial substrates required for rapid cancer cell growth and proliferation, thereby stalling cancer progression. These effects collectively contribute to a significant decrease in cell proliferation, highlighting diclofenac's ability to interfere with cancer metabolism directly. Diclofenac also exhibits pronounced proapoptotic activity through the inhibition of β‐catenin signaling. This is mediated by the elevated expression of peroxisome proliferator‐activated receptor‐γ (PPAR‐γ), a nuclear receptor that, when activated, can suppress β‐catenin, a protein involved in promoting cell growth and survival.^[^ ^12^ ^]^ By inhibiting β‐catenin, diclofenac promotes apoptotic pathways within cancer cells, facilitating their programmed cell death (PCD). Furthermore, diclofenac has been observed to induce apoptosis by increasing the levels of intracellular reactive oxygen species (ROS).^[^ ^13^ ^]^ The accumulation of ROS leads to oxidative stress within cancer cells, triggering apoptosis through the inhibition of Akt phosphorylation via the PI3 kinase (PI3K) signaling pathway. This pathway is crucial for cell survival and growth, and its disruption is a significant strategy in cancer therapy. In studies involving leukemic cell lines such as HL‐60 and THP‐1, diclofenac induced apoptosis through the activation of growth arrest and DNA damage‐inducible protein 45α (GADD45α) expression, followed by the activation of the c‐Jun *N*‐terminal kinase (JNK).^[^ ^14^ ^]^ This process is initiated by the activation of AP‐1 family transcription factors, including c‐Jun, JunB, and Fra‐2, which play roles in cellular stress responses and apoptosis. Lastly, diclofenac's ability to induce apoptosis is further supported by its role in increasing the expression of the nonsteroidal anti‐inflammatory drug‐activated gene 1 (NAG‐1).^[^ ^15^ ^]^ NAG‐1 is associated with proapoptotic activities in various cancer types, underlining diclofenac's multifaceted approach to inducing cancer cell apoptosis through both direct and indirect mechanisms. The diclofenac scaffold thus proves an excellent foundation for further modification enhancing its potential as a cancer therapeutic.

Following the success of integrating carboranes into the framework of COX inhibitors such as mefenamic acid,^[^ ^16^ ^]^ fenoprofen,^[^ ^17^ ^]^ flurbiprofen,^[^ ^18^ ^]^ acetylsalicylic acid,^[^ ^19^ ^]^ celecoxib,^[^ ^20^ ^]^ and rofecoxib,^[^ ^21^ ^]^ we aim to further this approach.^[^ ^22^ ^]^ Carboranes, particularly the *closo*‐dicarbadodecaboranes (C_2_B_10_H_12_), are polyhedral clusters known for their structural versatility and stability, featuring substitutable carbon atoms, which, analogous to benzene derivatives, are divided into three distinct isomeric forms, *ortho*, *meta*, and *para*.^[^ ^23^, ^24^ ^]^ These clusters are linked by a robust multielectron–multicenter bond network, forming a sigma‐aromatic structure with unique properties useful in medicinal chemistry, leading to increased drug selectivity and reduced toxicity.^[^ ^25^, ^26^ ^]^

**Scheme 1:**
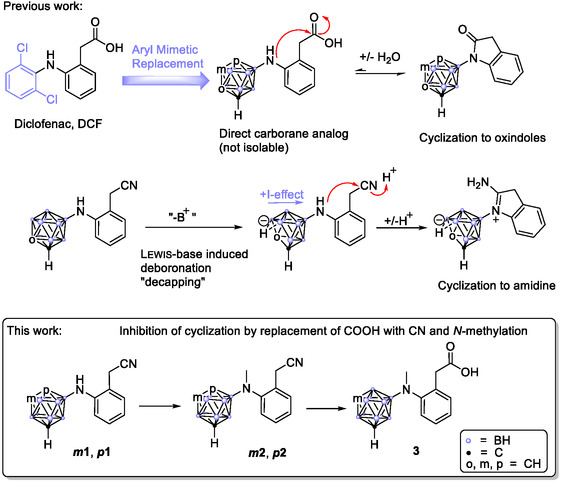
Synthetic concept for a direct carborane‐substituted diclofenac analog through aryl mimetic replacement. To prevent the preferred formation of lactams or cyclic amidines, a less‐reactive nitrile intermediate is synthesized and the free amine is trapped by *N*‐methylation.

In our previous work,^[^ ^27^ ^]^ we synthesized oxindole derivatives of *N*‐carboranyl diclofenac analogs (**Scheme** **1**, top right). Attempts to hydrolyze the lactam ring to obtain a free carboxylic acid were unsuccessful due to rapid reformation, most likely driven by the electron‐rich nitrogen, influenced by the strong +*I*‐effect of the boron‐linked cluster. Also, attempts with a nitrile group instead of a carboxylic acid led to the formation of an extraordinarily stable cyclic amidine species through the intermediate *nido*‐cluster deboronation. These compounds nonetheless proved highly effective against several cancer cell lines with IC_50_ values in the single‐digit micromolar range and showed high COX affinity.

To refine structure–activity relationships and build upon our previous work with cyclic diclofenac analogs, in the present work, we focused on designing open‐chain derivatives of carborane‐substituted diclofenac which have a closer structural similarity to the original substance (Scheme 1, bottom). This is either achieved by altering the nucleophile or the electrophile of the carboranyl diclofenacs: Therefore, on the one hand, the open‐ring form was trapped through tertiary modification of the nucleophilic nitrogen atom to impede the lactam reformation. This approach includes creating a series of *N*‐methyl analogs of diclofenac, inspired by previous successful modifications reported in the literature, which showed efficacy against cancer cell lines, sometimes surpassing diclofenac itself.^[^ ^9^, ^28^ ^]^ On the other hand, in view of the fact that the carboxylic acid functionality is not always essential for COX inhibition or cytotoxic effects, we also synthesized and evaluated carboranyl‐substituted nitrile derivatives. To provide a broader comparative framework, corresponding *N*‐methyl‐ and nitrile‐substituted phenyl analogs were also included in the study.^[^ ^29^ ^]^ Ultimately, this work extends the compound library of carborane‐based diclofenac analogs while allowing for a comparative evaluation of boron cluster‐based and isosteric phenyl modifications.^[^ ^30^ ^]^ Through flow cytometry and molecular docking studies, we aim to elucidate the mechanism of action and contribute to a deeper understanding of structure–activity relationships in this emerging class of anticancer agents.

## 2 Results and Discussion

### 2.1 Synthesis

Building on insights from our previous work and inspired by the carborane‐substituted mefenamic acid and isonimesulide analogs from Useini et al. we embarked on a synthetic strategy utilizing 2‐aminophenylacetonitrile and iodocarboranes.^[^ ^16^, ^31^ ^]^ These were fused in a Buchwald‐Hartwig‐type coupling reaction facilitated by palladium catalysis, specifically using SPhosPdG4 (methanesulfonato(2‐dicyclohexylphosphino‐2’,6’‐dimethoxy‐1,1’‐biphenyl)(2’‐methylamino‐1,1’‐biphenyl‐2‐yl)palladium(II)), which has demonstrated high efficacy in these reactions.^[^ ^32^, ^33^ ^]^ We initiated our synthesis with a literature‐known procedure to obtain 9‐iodo‐*meta*‐carborane and 2‐iodo‐*para*‐carborane from the native carboranes and iodine in 80% and 96% yield.^[^ ^16^, ^33^ ^]^

Our prior experiences with *ortho*‐carborane, where decapping led to *nido*‐cluster formation and increased electron density at the amine, promoting cyclization to form a cyclic amidine (Scheme 1, middle), guided our decision to focus exclusively on *meta*‐ and *para*‐carborane due to their enhanced stability against deboronation.^[^ ^23^, ^27^ ^]^

**Scheme 2:**
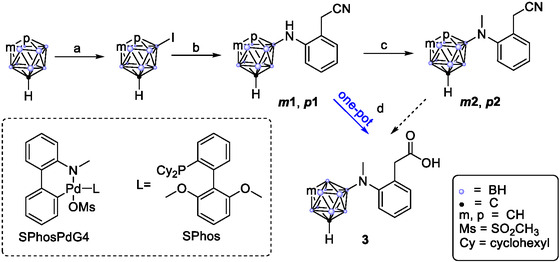
Synthesis of the diclofenac‐inspired carborane compounds ***m*** **1**, ***p*** **1**, ***m*** **2**, ***p*** **2,** and **3**. Reaction conditions. a) I_2_, H_2_SO_4_, HNO_3_, CH_3_COOH, 60–80 °C, 80–96%; b) 2‐aminophenylacetonitrile, KO^*t*^Bu, SPhosPdG4, 1,4‐dioxane, 90 °C, 71% and 60%; c) NaBH_4_, 37% aq. CH_2_O, 3 m H_2_SO_4_ aq., THF, 0 °C to RT, 5 min, 90% and 88%; d) NaBH_4_, 37% aq. CH_2_O, 8 m H_2_SO_4_ aq., THF, 90 °C, 18 h, 56%.

**Figure 1:**
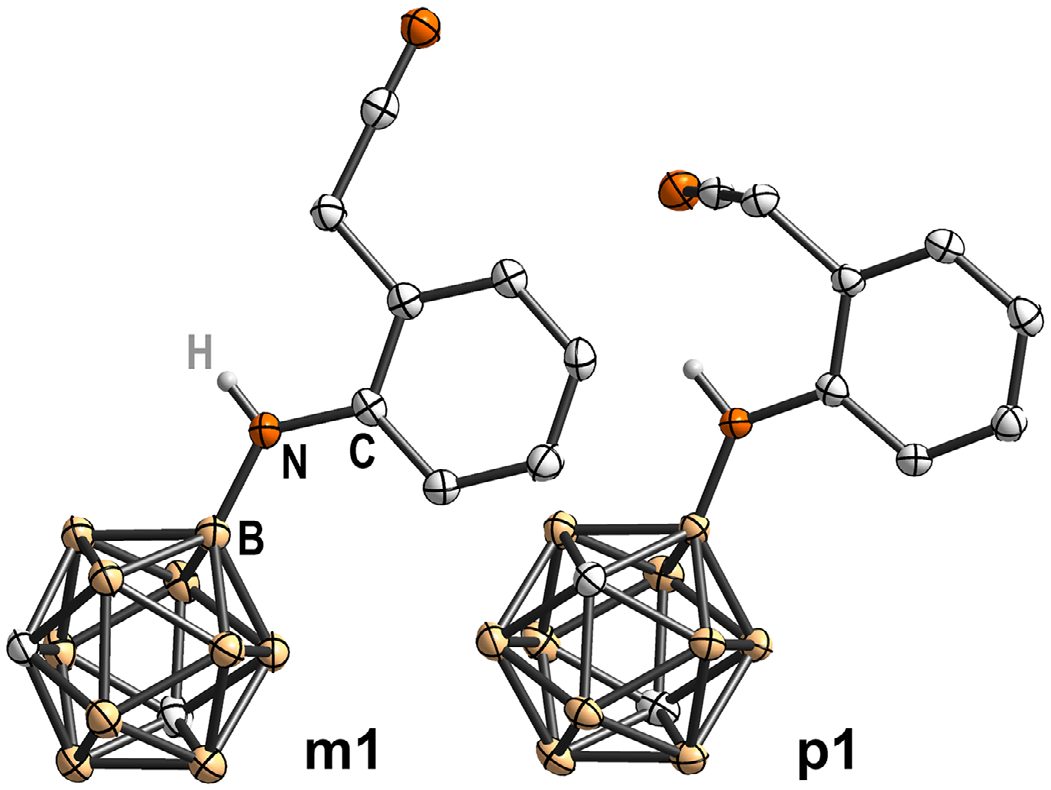
Molecular structures of the secondary arylamines ***m*** **1** and ***p*** **1** with displacement thermal ellipsoids drawn at 50% probability level. Detailed crystallographic parameters can be found in the Supporting Information.

The palladium‐mediated coupling reactions to synthesize the secondary amines proceeded smoothly using potassium *tert*‐butoxide and 1,4‐dioxane affording the diaryl amines ***m*** **1** and ***p*** **1** in 71% and 60% yield, respectively (**Scheme** **2**b). The resultant amines readily crystallized from a saturated dichloromethane solution layered with *n*‐hexane, allowing for detailed structure elucidation via X‐ray diffraction (**Figure** **1**, Supporting Information).^[^ ^49^ ^]^

Advancing to methylation, we initially avoided alkyl halide methods due to their probability to form quaternary ammonium salts. Instead, we employed an Eschweiler–Clarke‐like reductive amination using an aqueous formaldehyde solution, sulfuric acid, and sodium borohydride in tetrahydrofuran (THF).^[^ ^34^ ^]^ This approach prevents quaternation by forming an intermittent reactive iminium species, which is then efficiently reduced by the borohydride to form the monomethylamine. This reaction proved highly effective, completing within minutes for both the *meta*‐ and *para*‐carborane derivatives, yielding the *N*‐methylated nitriles ***m*** **2** and ***p*** **2** as yellow oils in 90% and 88% yield. The final synthetic step involved the hydrolysis of the nitrile group to the corresponding carboxylic acid. We initially tested this on a small scale with ***m*** **2** using a mixture of 60% aqueous sulfuric acid and acetic acid, which proved successful. Subsequently, we optimized the conditions to align with those of the reductive amination, enabling a seamless integration of both steps into a one‐pot reaction cascade (Scheme 2d). Starting from the secondary amines under identical starting conditions, it thus was possible to either isolate the *N*‐methylated nitrile ***m*** **2** after 5 min at room temperature (RT), while heating to 90 °C and extending the reaction overnight facilitated the conversion of ***m*** **1** directly to the *N*‐methylated phenylacetic acid **3** in 56% yield in the case of the *meta*‐carborane. In contrast, only the nitrile ***p*** **2** was obtained for the *para*‐carborane derivative, with several attempts to hydrolyze the nitrile under varied conditions (basic, acidic, extended (microwave oven) heating) failing to yield the desired product. Given the prohibitively high cost of *para*‐carborane and a rational prototype at hand, further experimentation was discontinued.

**Scheme 3:**
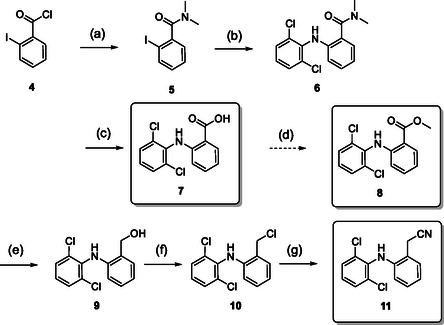
Synthesis of the phenyl analogs **7**, **8**, and **11**. Reaction conditions:^[^ ^36^ ^]^ a) 40% aq. NH(CH_3_)_2_, 0 °C, 30 min; b) 2,6‐dichloroaniline, K_2_CO_3_, Cu (powder), CuI, toluene, reflux, 7 d; c) NaOH, EtOH, H_2_O, 90 °C, 18 h, 75% (from **4**); d) MeOH, SOCl_2_, 0–60 °C, 18 h, 74%; e) LiAlH_4_, THF, 0–74 °C, 16 h, 77%; f) SOCl_2_, pyridine, THF, RT, 30 min; g) NaCN, DMSO, 45 °C, 1.5 h, 74%.

With this new set of carborane‐substituted diclofenac analogs synthesized, our next step was to prepare respective phenyl analogs as reference substances for comparative analysis and further biological evaluation. Additionally, we included two anthranilic acid intermediates **7** and **8** (**Scheme** **3**) in our tests. These intermediates, while previously tested in different contexts, were in our knowledge not evaluated for their anticancer and COX inhibition potential.^[^ ^35^ ^]^

The synthesis process for the analogous nitrile **11**, as described by Tateishi et al.^[^ ^36^ ^]^ started from 2‐iodobenzoic acid chloride **4** treated with aqueous dimethylamine as a protective group for subsequent reactions. The resulting *N*,*N*‐dimethylamide **5** was coupled with 2,6‐dichloroaniline via a copper‐mediated Ullmann‐type reaction to produce the secondary diarylamine **6**.

Subsequently, the amide protective group was cleaved off with aqueous sodium hydroxide to yield the anthranilic acid derivative **7**. This derivative served as starting material for methyl ester **8** that was obtained by simple esterification with thionyl chloride and methanol in 74% yield. For the further synthesis of the nitrile **11**, carboxylic acid **7** was reduced to benzyl alcohol **9** using lithium aluminum hydride in 77% yield. The alcohol was chlorinated with thionyl chloride, followed by substitution with sodium cyanide in dimethyl sulfoxide (DMSO) to produce the desired nitrile **11** in 74% yield. Efforts to synthesize the *N*‐methyl nitrile through various synthetic routes ultimately proved unsuccessful.

Consequently, we synthesized the *N*‐methylated phenylacetic acid derivative from commercially available diclofenac, adhering to a procedure described in the literature.^[^ ^9^ ^]^ This process involved reducing diclofenac to phenethyl alcohol **12**, which was then protected to form silyl ether **13**.

**Scheme 4:**
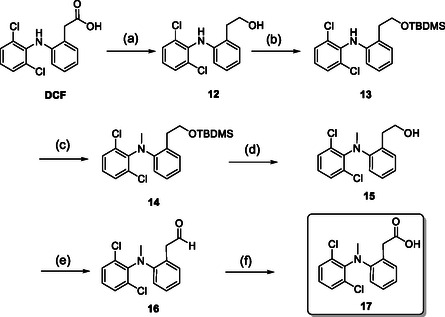
Synthesis of *N*‐methylated diclofenac **17**. Reaction conditions:^[^ ^9^ ^]^ a) LiAlH_4_, THF, 0–74 °C, 16 h, 72%; b) *tert*‐butyldimethylsilyl chloride, dimethylaminopyridine, CH_2_Cl_2_, RT, 1 h, quant.; c) NaH (60 wt% in mineral oil), CH_3_I, THF, 0 °C–RT, 1 h; d) THF, (NBu_4_)F, RT, 67% (from **13**); e) 1,1,1‐triacetoxy‐1,1‐dihydro‐1,2‐benziodoxol‐3(1*H*)‐on, CH_2_Cl_2_, RT, 90 min; f) NaClO_2_, NaH_2_PO_4_, 2‐methyl‐2‐butene, ^*t*^BuOH, H_2_O, RT, 1 h, 59% (from **15**).

The nitrogen was methylated using sodium hydride and iodomethane, and after deprotection, the alcohol **15** was oxidized back to the carboxylic acid **17** via the aldehyde (**16**) in a two‐step protocol using Dess–Martin periodinane and Pinnick oxidation (**Scheme** 4).

With this comprehensive set of carborane and dichlorophenyl‐substituted derivatives at hand, we proceeded to assess their potential through a series of biological and computational tests.

### 2.2 COX Inhibition and Molecular Docking

In our investigation of COX inhibition potential, the synthesized compounds were evaluated in vitro against human recombinant COX‐2, using diclofenac as the reference standard. The assays were conducted using a commercial COX Fluorescent Inhibitor Screening Assay Kit (Cayman Chemical, Ann Arbor, MI). In a preliminary assay, the compounds were tested for their inhibitory potential at a concentration of 100 μM (**Table** **1**). Among the nine compounds tested, only the dichlorophenyl nitrile **11** exhibited modest COX‐2 inhibition, achieving 34% enzyme inhibition. From the remaining compounds only ***m*** **1**, ***p*** **1**, ***m*** **2**, ***p*** **2**, **3**, **11**, and **17** demonstrated significant inhibition ranging from 5 to 26%. However, in comparison to established COX inhibitors, the binding affinity to COX‐2 is rather neglectable.

**Table 1:** COX‐2 inhibition potential at 100 μM and IC_50_ values [μM] of diclofenac (DCF), carborane derivatives (***m*1**, ***m*2**, ***p*1**, ***p*2**, and **3**), and dichlorophenyl analogs (**7**, **8**, **11**, and **17**) on cancer cell lines. Data is presented as the mean of duplicate determination of percent inhibition of COX‐2 at 100 μM and as mean ± standard deviation (SD) of three independent experiments obtained by MTT and CV tests.

#	Percent inhibition of COX‐2 @100 μM	IC_50_ [μM]			
		Assay	MC38	HCT116	HT29
**DCF**	102	MTTa)	>100	>100	>100
		CVb)	>100	>100	>100
*m*1	26	MTT	17.6 ± 5.1	23.0 ± 5.1	20.9 ± 4.6
		CV	24.5 ± 0.3	33.3 ± 2.1	33.0 ± 3.2
*p*1	9	MTT	12.7 ± 0.9	23.0 ± 5.1	20.9 ± 4.6
		CV	29.9 ± 1.4	40.3 ± 3.5	35.7 ± 2.6
*m*2	5	MTT	17.7 ± 0.8	24.1 ± 1.6	26.4 ± 2.4
		CV	37.8 ± 1.1	43.0 ± 3.4	34.4 ± 1.9
*p*2	n.i.c)	MTT	17.7 ± 3.0	20.0 ± 1.2	25.8 ± 3.0
		CV	32.7 ± 1.6	48.2 ± 2.1	41.9 ± 2.1
3	19	MTT	>100	>100	>100
		CV	>100	>100	>100
7	n.i.	MTT	>100	>100	>100
		CV	>100	>100	>100
8	n.i.	MTT	51.6 ± 0.6	32.7 ± 0.7	67.7 ± 6.2
		CV	49.7 ± 4.3	42.0 ± 3.2	76.2 ± 1.7
11	34	MTT	24.6 ± 5.6	40.4 ± 2.9	48.5 ± 5.6
		CV	46.3 ± 4.2	63.4 ± 5.5	68.8 ± 2.4
17	17	MTT	>100	>100	>100
		CV	>100	>100	>100

a) 3‐(4,5‐Dimethylthiazol‐2‐yl)‐2,5‐diphenyltetrazolium bromide;

b) Crystal violet;

c) n.i. = no inhibition, meaning inhibition <5%.

**Figure 2:**
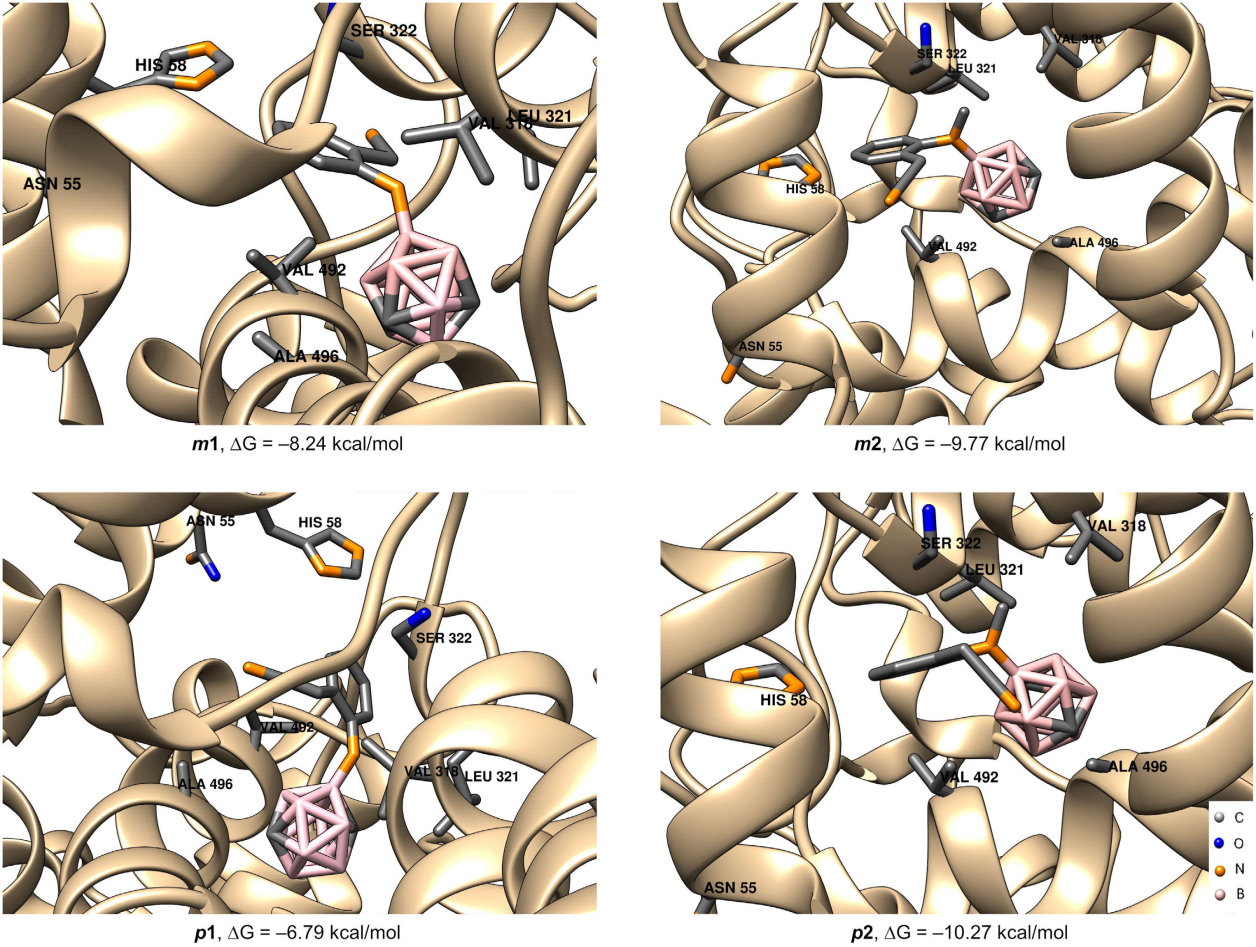
In silico investigation of the binding modes of carborane derivatives ***m*** **1** *, **p*** **1**, ***m*** **2**, and ***p*** **2** based on docking in COX‐2. The structure with PDB ID 4Z0L was used as the starting point for docking.^[^ ^37^ ^]^ The highest ranked docked positions of compounds ***m*** **1** *, **p*** **1**, ***m*** **2**, and ***p*** **2** are shown together with the labeled amino acid residues essential for the noncovalent interactions with the ligands (Leu321, Ser322, His58, Asn55, Val392, Ala496).

To further investigate the underlying reasons for this low COX‐2 activity, we conducted molecular docking based on the position of the cocrystal structure of the carborane‐substituted COX‐2 inhibitor *nido*‐indoborine and COX‐2 reported in the literature (PDB ID: 4Z0L).^[^ ^37^, ^38^ ^]^ The initial carborane derivative was removed from the binding pocket, and the structures of ***m*** **1**, ***p*** **1**, ***m*** **2**, and ***p*** **2** were docked in the similar position (**Figure** **2**). All four structures share a similar arrangement, engaging in noncovalent interactions with analogous amino acid residues (Figure 2).

While for ***m*** **1** and ***p*** **1** the structures were based on their crystal structures, ***m*** **2** and ***p*** **2** structures were modeled in silico. Among the two secondary amine compounds ***m*** **1** and ***p*** **1**, meta‐carborane derivative ***m*** **1** (ΔG = –8.24 kcal mol^−1^) displayed superior binding potential with COX‐2. In contrast, the para‐carborane derivative ***p*** **1** exhibited the weakest binding affinity Δ*G* at –6.79 kcal mol^−1^. This is in alignment with the results from the COX‐2 assays (Table 1), where ***m*** **1** showed the second highest enzyme inhibition of 26% as opposed to 9% for ***p*** **1**. With the tertiary amines ***m*** **2** and ***p*** **2**, despite reasonable docking poses, our predicted binding energies (ΔG = –9.77 kcal mol^−1^ for ***m*** **2** and –10.27 kcal mol^−1^ for ***p*** **2**) showed no clear correlation with the in vivo enzyme inhibition outcomes (5% inhibition for ***m*** **2**% and 5% for ***p*** **2**), highlighting that a more favorable docking score did not translate into significantly higher biological activity. This lack of correlation is consistent with the known limitations of docking‐based affinity predictions like the neglect of explicit solvation and entropic effects, which would require a higher level of theory (e.g., quantum mechanics model). On the other hand, in vivo efficacy is influenced by pharmacokinetic and cellular factors beyond the static binding event including possible off‐target interactions or alternative mechanisms of action not captured by the docking model.^[^ ^39^ ^]^

### 2.3 Cytotoxicity

The anticancer efficacy of the five carborane derivatives ***m*** **1**, ***p*** **1**, ***m*** **2**, ***p*** **2**, and **3**, along with their dichlorophenyl reference compounds (**DCF**, **7**, **8**, **11**, and **17**), were evaluated against three cancer cell lines: mouse colon adenocarcinoma (MC38), human colorectal carcinoma (HCT116), and human colorectal adenocarcinoma (HT29). These cell lines were treated with a spectrum of concentrations of each compound, ranging from 1.56 to 100 μM, for a period of 72 h.

Cell viability was assessed using two different assays: 3‐(4,5‐dimethylthiazol‐2‐yl)‐2,5‐diphenyltetrazolium bromide (MTT) and crystal violet (CV). Due to inconsistencies in the data obtained with the MTT test, the evaluation relied primarily on the results of the CV assay and was validated by light microscopy in all cases to ensure reliability. We conducted in vitro testing of all our synthesized compounds against the indicated cancer cell lines (Table 1). Interestingly, none of the carboxylic acid compounds, including diclofenac (**3**, **7**, **17**, and **DCF**), demonstrated any cytotoxic effects. In contrast, all nitrile derivatives (***m*** **1**, ***p*** **1**, ***m*** **2**, ***p*** **2**, and **11**) exhibited significant cytotoxicity in all three cell lines tested.

Among these, the four carborane‐substituted compounds (***m*** **1**, ***p*** **1**, ***m*** **2**, and ***p*** **2**) generally presented lower IC_50_ values compared to the phenyl derivatives (**8** and **11**), indicating enhanced potency caused by introduction of a carborane moiety. Notably, the *N*‐methylated derivatives ***m*** **2** and ***p*** **2** were less cytotoxic than their corresponding secondary amines ***m*** **1** and ***p*** **1**, suggesting that the introduction of a methyl group at the nitrogen atom may slightly attenuate the cytotoxic effect (Figure S4, Supporting Information).

Furthermore, a comparison within the carborane series revealed that the meta‐carborane substituted derivatives ***m*** **1** and ***m*** **2** exhibited the lowest IC_50_ values, highlighting the influence of cluster carbon arrangement on biological activity.^[^ ^26^ ^]^ The best‐performing compound within this group was identified as ***m*** **1**, with an IC_50_ concentration of 24.5 ± 0.3 μM against the MC38 cell line. Close contenders included the *N*‐methylated analog ***m*** **2** with an IC_50_ concentration of 37.8 ± 1.1 μM and the dichlorophenyl analog **11** with an IC_50_ concentration of 46.3 ± 4.2 μM.

Finally, the most active compounds ***m*** **1**, ***m*** **2,** and **11** exerted cytotoxic activity against primary mouse embryonic fibroblasts NIH/3T3, albeit to a lesser extent, and showed moderate but significant selectivity against the malignant phenotype with selectivity indices (SI) varying between 1.1 and 2.65 (Figure S5 and Table S1, Supporting Information). These three compounds were selected for further biological evaluation to elucidate their potential as cancer therapeutics (**Table** **2**).

**Table 2:** SI (IC_50_ value of the primary NIH/3T3 cell line vs. cancer cell lines). SI > 1 indicates greater activity of the compound against cancer cells.

Compounds	Cancer cell lines		
	MC38	HCT116	HT29
*m*1	2.65	1.88	1.9
*m*2	1.57	1.38	1.73
11	1.58	1.15	1.1

### 2.4 Mechanisms of Action

**Figure 3:**
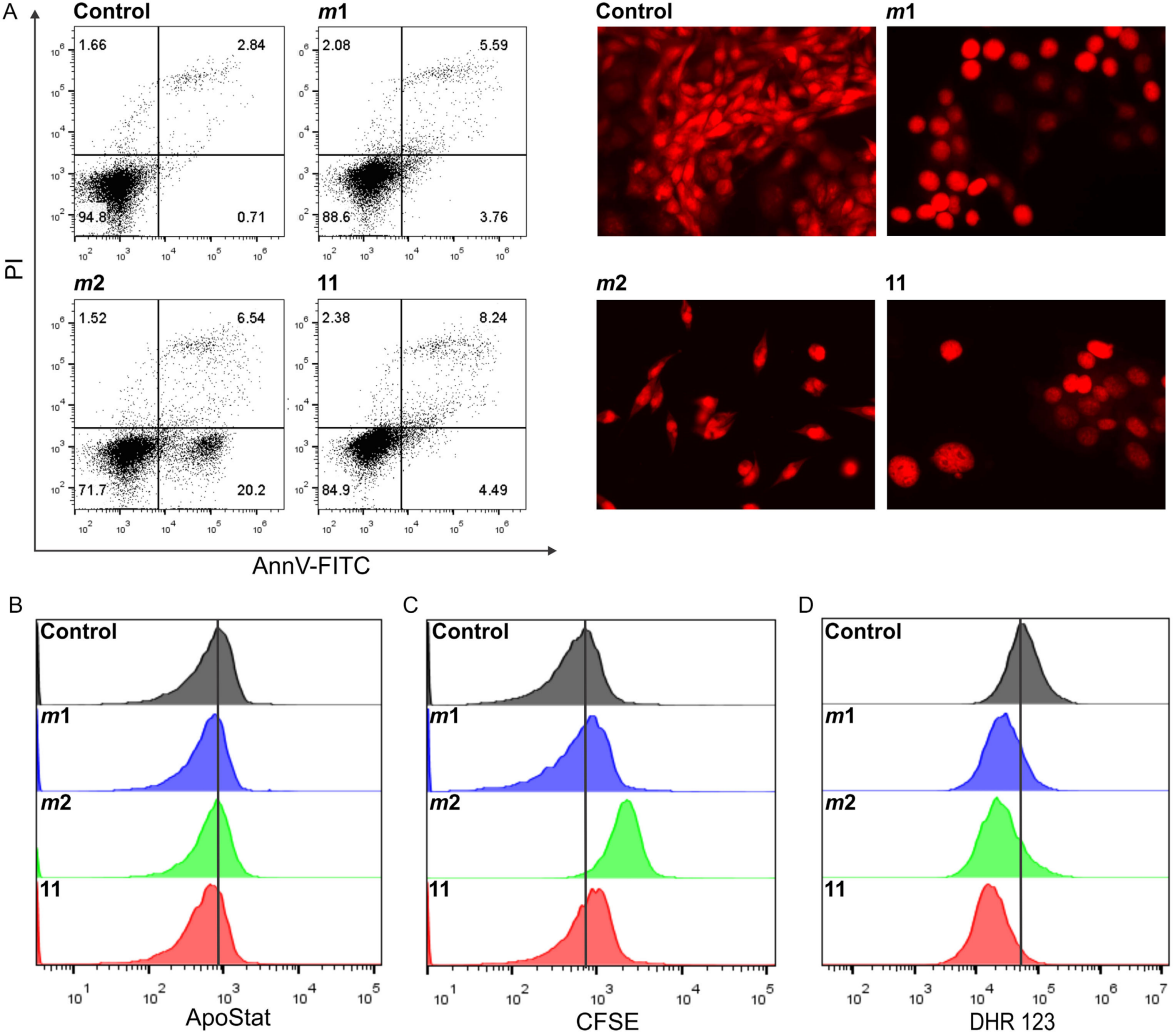
The mechanism of action of compounds ***m*** **1**, ***m*** **2**, and **11** on MC38 cells, treated with IC_50_ concentrations of the respective compounds for 72 h, was evaluated by flow cytometry and fluorescence microscopy (400 × magnification) after the corresponding staining: AnnV‐FITC/PI (A, left panel), PI (A, right panel), ApoStat (B), CFSE (C), and DHR 123 (D). Representative charts and micrographs from three independently performed experiments are shown.

In order to elucidate in detail the mechanisms of action of the best compounds in the class ***m*** **1**, ***m*** **2**, and **11** on the MC38 cell line, we used different fluorescent stainings to label, quantify, and characterize the presence and type of cell death, the rate of cell proliferation, and the production of ROS and reactive nitrogen species (RNS) in response to the treatment. The collected data were analyzed by flow cytometry and subsequently verified by fluorescence microscopy. MC38 cells were seeded and treated with the IC_50_ concentrations of each of the three compounds (for 72 h). Staining with Annexin V‐FITC (AnnV‐FITC) in combination with propidium iodide (PI), which was used to distinguish between early (AnnV‐FITC^+^/PI^−^) and late apoptotic/necrotic cells (AnnV‐FITC^+^/PI^+^), showed an increased percentage of apoptotic cells in cultures exposed to compound ***m*** **2**, preferentially in the early phase of this process. In parallel, compound **11** led to an enhanced apoptotic process compared to ***m*** **1**, but to a much lesser extent than ***m*** **2** (**Figure** **3**A, left panel; Figure S6, Supporting Information).

PI staining of paraformaldehyde‐fixed cells after incubation for 72 h with ***m*** **1**, ***m*** **2**, and **11** allowed profiling of the size, morphology, chromatin density, and structure of the cell nuclei, providing insight into the effects of the compounds, disclosure of differences between them, and comparison with previously obtained data.

As shown in Figure 3A (right panel), the cells exposed to ***m*** **1** exhibited a rounded morphology, but without significant changes in genetic material that could be described as signs of apoptosis. The rounded morphology indicates possible changes in their adhesive properties and their tendency to detach from the bottom of the plate wells, suggesting the induction of anoikis, a PCD that occurs after cell detachment from the surrounding tissue, as a possible mode of action of the drug. Anoikis is described as a cell death that hinders the cancer cell dissemination and usually ends with the realization of the apoptotic process.^[^ ^40^ ^]^ This suggests the possibility that the drug ***m*** **1** could intervene in the metastatic process, which needs to be investigated further.^[^ ^40^ ^]^ On the other hand, compound ***m*** **2** caused changes typical for apoptosis: apoptotic nuclei with condensed chromatin and abnormal shape.

Finally, in cultures exposed to compound **11**, large nuclei with a chromatin structure characteristic of senescent cells were evident, along with cells with duplicated genetic material, but incomplete cell division. In contrast to numerous examples of diclofenac‐induced caspase‐dependent apoptosis, none of the treatments applied resulted in an increase in caspase activity, indicating that the apoptotic process, regardless of its intensity and dynamics, cannot be explained as caspase dependent (Figure 3B; Figure S6, Supporting Information).^[^ ^41^ ^]^ In the last decade, a growing body of evidence underlines the existence of PCD independent of caspases qualified as the death backup system. Its important role in effective removal of abnormal cells from the organism is well confirmed. Similar to classical apoptotic pathways, mitochondria play a central role in this process orchestrating a series of events resulting in cell death.^[^ ^42^ ^]^ After the same incubation period, an increased percentage of undivided cells was observed in all cell cultures exposed to the tested compounds, as measured by an enhanced mean value of fluorescence of cells stained with carboxyfluorescein diacetate succinimidyl ester (CFSE) compared to the untreated controls (Figure 3C; Figure S6, Supporting Information). This effect qualified all compounds as antiproliferative. In parallel, the reduced production of ROS/RNS detected by the cell permanent‐specific fluorescent dye DHR 123 was observed after 72 h of incubation with each of the compounds (Figure 3D). The mentioned phenomenon may be in the background of viability reduction and diminished proliferative capacity of MC38. In a scenario where ROS are essential mediators of caspase‐independent cells death, compounds with ROS scavenging capacities compromised the cell death process.^[^ ^42^ ^]^ Since the antitumor potential of experimental therapeutics tested in this study resulted in caspase‐independent cell death under ROS‐depleted conditions, it can be concluded that the outcome of the treatment is a consequence of modulation of cell death pathways rather than cell damage.

**Figure 4:**
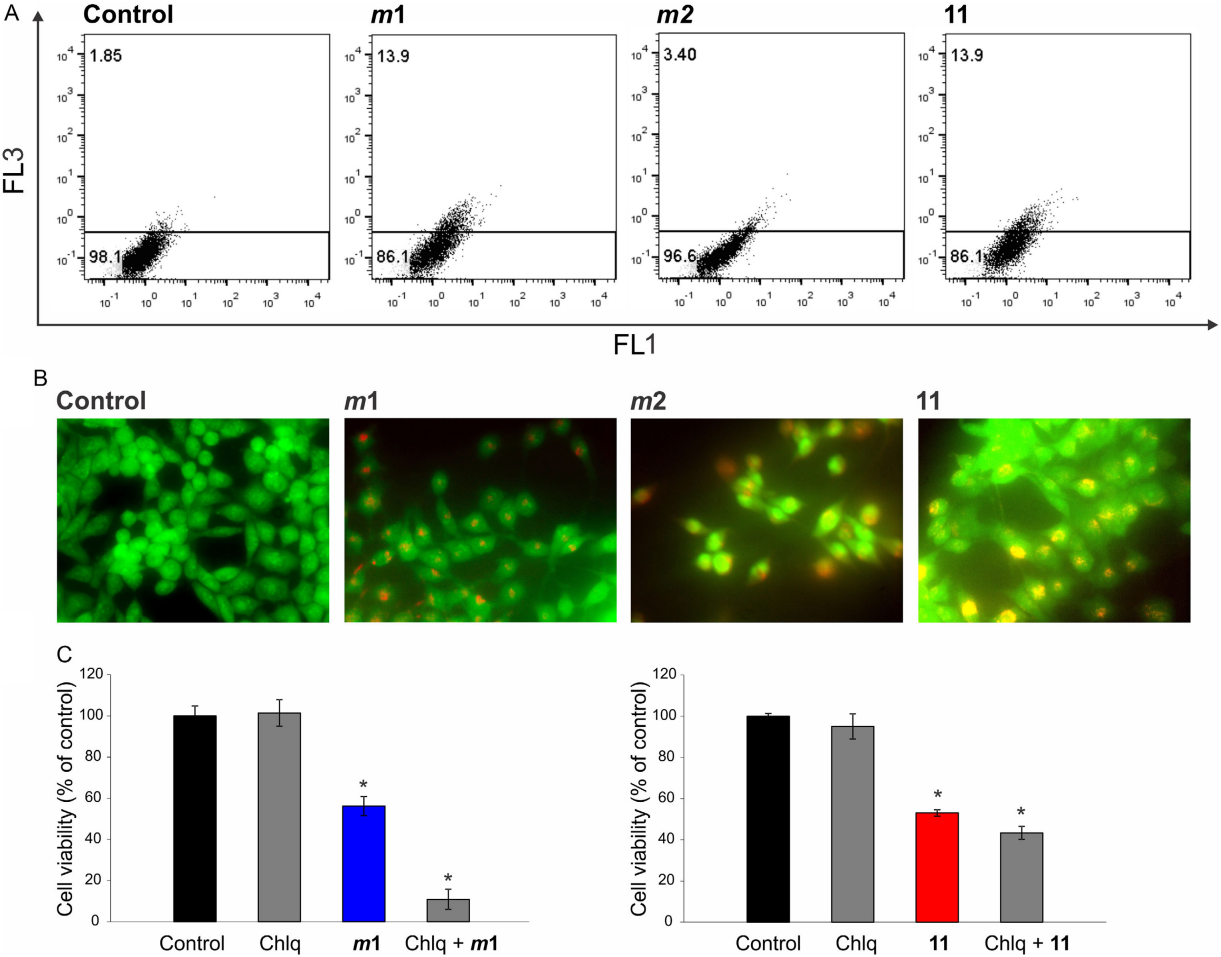
A) Autophagy was assessed by flow cytometry, B) fluorescence microscopy (400 × magnification), and C) viability assay. MC38 cells were exposed to IC_50_ concentrations of compounds ***m*** **1**, ***m*** **2**, and **11** for 72 h followed by AO staining (A,B). Representative charts and micrographs from three independently performed experiments are shown. Cell viability was determined upon simultaneous treatment of the autophagy inhibitor chloroquine (Chlq) with compound ***m*** **1** (C, left graph) and compound **11** (C, right graph). * p < 0.05 compared to the control.

High level of the intrinsic production of reactive species involved in the essential metabolic demands of these cells is well documented, and the maintenance of cancer cell viability is closely related to their intracellular availability.^[^ ^43^ ^]^ In response to cytotoxic stimuli and proapoptotic signals, cells often enhance the autophagic process to prevent intracellular destruction by impaired digestion of damaged structures.^[^ ^44^ ^]^ However, under certain conditions, self‐digestion can serve as a form of cell death known as autophagic cell death or PCD type 2.^[^ ^45^ ^]^ Compounds ***m*** **1** and **11** enhanced the formation of autophagosomes, while compound ***m*** **2**, characterized by a strong ability to induce apoptosis in a selected cell line, did not significantly trigger this process (**Figure** **4**A; Figure S7, Supporting Information).

This phenomenon was also confirmed by acridine orange (AO) staining of cell cultures, where yellow‐to‐red intracellular dots resembling autophagosomes can be seen in the cell cytoplasm, while an increase in green intensity indicates condensation of genetic material and, thus, an ongoing apoptotic process (Figure 4B). Simultaneous treatment of compounds ***m*** **1** and **11** with the autophagy inhibitor chloroquine (Chlq) showed a cytoprotective role of autophagy, suggesting that intense autophagy counteracts the realization of the apoptotic program and, thus, at least partially neutralized the cytotoxicity of the drugs (Figure 4C).

Taken together, the results showed that all tested compounds exerted their antitumor activities predominantly through inhibition of proliferation and induction of apoptosis, with the most profound proapoptotic capacity observed after treatment with ***m*** **2** potentiated by inhibition of autophagy. Apart from the similar cytotoxic potential of all tested compounds and according to the calculated IC_50_ concentrations, their effects varied from typical apoptotic changes in ***m*** **2**‐exposed cultures to cell detachment and the anoikis‐like action pathway in response to ***m*** **1** and induction of senescence in the presence of **11**. In the background of all treatments, depletion of ROS/RNS is consistently observed, indicating a fundamental contribution of endogenously produced reactive molecules to the maintenance of colorectal cancer cell viability and highlighting their importance and the possibility of their use as a platform for therapeutic intervention.

## 3 Conclusion

In summary, in this study, we elucidated the synthesis and characterization, as well as conducted a biological and computational evaluation of a series of carborane‐substituted diclofenac analogs for their COX‐inhibitory and anticancer potential. As described in preliminary work, first‐generation carborane‐substituted diclofenac analogs easily undergo intramolecular cyclization to form oxindoles or amidines. In the present work, we describe a synthetic strategy to obtain open‐chain analogs by either replacing the carboxylic acid moiety with a less reactive nitrile (***m*** **1** and ***p*** **1**) and/or by methylation of the secondary amine (***m*** **2**, ***p*** **2,** and **3**). In a temperature‐ and time‐controlled one‐pot procedure, methylation and generation of the carboxylic acid could be merged successfully. Additionally, the respective dichlorophenyl analogs were synthesized and all compounds were biologically evaluated. In an in vitro test, none of the compounds showed promising COX‐2 inhibition (0–34% enzyme inhibition) indicating mostly COX‐independent proapoptotic properties. In silico analysis of the interaction of ***m*** **1**, ***p*** **1**, ***m*** **2**, and ***p*** **2** with COX‐2 revealed similar binding poses for all four compounds. The calculated binding energy within the secondary amines was lowest for ***m*** **1**, which is in accordance with the inhibitory potential determined in the COX‐2 assay. For the tertiary amines ***m*** **2** and ***p*** **2** however, no clear correlation between in silico and in vivo experiments was observed.

In mouse colon adenocarcinoma (MC38), human colorectal carcinoma (HCT116), and human colorectal adenocarcinoma (HT29) cell lines, all nitrile‐based compounds showed moderate‐to‐strong cytotoxic activity, while all carboxylic acid derivatives, including DCF, were found to be biologically inert. Within the active nitriles, the carborane derivatives were found to be more efficient than their phenyl analog, and *N*‐methylation generally resulted in slightly weaker activity. The best‐in‐class nitrile compounds ***m*** **1**, ***m*** **2**, and **11** were selected for further evaluation of the drug's mechanism of action by flow cytometry and fluorescence microscopy on the most sensitive MC38 cell line. All three compounds had potent antiproliferative activity and different potential to promote caspase‐independent apoptosis and to acquire prosenescence properties. The enhanced autophagy opposes the cytotoxicity of the drugs and provides an explanation for the moderate‐to‐low apoptosis in cultures exposed to **11** and ***m*** **1**, respectively, and the absence of autophagy is in accordance with the intense apoptosis observed in cultures treated with compound ***m*** **2**. All effects, apart from the differences between compounds, were uniformly associated with intracellular ROS/RNS depletion, suggesting that their cytotoxicity can be related to this.

Our results underline the potential of these carborane‐substituted diclofenac analogs for cancer therapeutic approaches and provide a basis for further research and development of more effective anticancer drugs. Future studies will focus on refining these compounds to improve their selectivity and efficacy, ultimately aiming to provide new therapeutic options for cancer treatment.

## 4 Experimental Section

4.1

4.1.1

##### General Information

Unless otherwise noted, all reactions were carried out in standard Schlenk technique using oven‐dried glassware that was heated to 600 °C (with a heat gun) under high vacuum and refilled with dry argon three times prior to use (securated). NMR spectra were recorded on a Bruker Avance DRX 400 MHz (^1^H NMR 400.16 MHz and ^13^C NMR 100.59 MHz) and a Bruker Avance III 400 MHz (^1^H NMR 400.20 MHz, ^13^C NMR 100.64 MHz) spectrometer. The chemical shift (δ) is given in ppm. ^1^H NMR spectra were either referenced to Si(CH_3_)_4_ as internal standard or to the solvent residual signal as internal reference for ^1^H and ^13^C NMR (CDCl_3_: δH 7.26; δC 77.0, (CD_3_)_2_SO: δH 2.50; δC 39.5). ^11^B NMR chemical shifts were calculated according to the ≡‐scale.^[^ ^46^ ^]^ Multiplicities were indicated, s (singlet), d (doublet), t (triplet), q (quartet), quint (quintet), sept (septet), m (multiplet), b (broad singlet); coupling constants (*J*) were in Hertz (Hz). High‐resolution mass spectra were recorded using a Bruker Daltonics Impact II with electrospray ionization and time‐of‐flight detection. The predicted mass spectra were generated using MestreLab Research MestReNova v14.1.0.‐24,037. Thin‐layer chromatography (TLC) analysis was performed on precoated silica gel 60 F_254_ slides and visualized by UV light (254 nm) or palladium staining (5% m/v PdCl_2_ solution in MeOH). Analytical high performance liquid chromatography (HPLC) analysis was carried out using a JASCO system equipped with an JASCO PU 4180 pump, an JASCO MD 4015 photodiode array detector, an JASCO AS 4050 autosampler, and a RHEODYNE column compartment by IDEX Health & Science. The system was operated using a Daicel Chiralpak IA column (amylose based with tris(3,5‐dimethylphenylcarbamate) immobilized on 5 μm silica gel, 6 × 25.0 mm). UV absorption was detected in the area of 209–338 nm with the respective solvent ratio of hexane to isopropanol at a flow rate of 1 mL min^−1^. Microwave‐assisted reactions were carried out in an Initiator 8 (Biotage AB) single‐mode microwave at 2450 MHz‐controlled irradiation using standard sealed microwave glass vials (20 mL). Reaction temperatures were monitored by an IR sensor on the outside wall of the reaction vials. Reaction times refer to hold times at the selected set temperature, not to total irradiation times. Diethyl ether, THF, toluene and 1,4‐dioxane were distilled from sodium/benzophenone and stored under argon over 4 Å molecular sieves. All other solvents were distilled prior to use. *n*‐Butyllithium was titrated with *N*‐benzyl benzamide prior to use and stored under argon.

Compounds **5**, **6**,**7**, **9**, **10**, **11**,^[^ ^36^ ^]^ **12**, **13**, **14**, **15**, **16**, **17**,^[^ ^9^ ^]^ and the iodocarboranes, 9‐iodo‐1,7‐dicarba‐*closo‐*dodecaborane(12) and 2‐iodo‐1,12‐dicarba‐*closo‐*dodecaborane(12)^[^ ^16^ ^]^ were prepared according to known literature procedures. All other chemicals were purchased from commercial sources and used as received.

##### Synthesis of Nitriles m1 and p1

In a 10 mL Schlenk tube containing a stir bar, the respective iodocarborane (81 mg, 0.3 mmol, 1.0 equiv.), 2‐aminophenylacetonitrile (40 mg, 0.3 mmol, 1.0 equiv.), potassium *tert*‐butoxide (40 mg, 0.35 mmol, 1.2 equiv.), and methanesulfonato(2‐dicyclohexylphosphino‐2’,6’‐dimethoxy‐1,1’‐biphenyl)(2’‐methylamino‐1,1’‐biphenyl‐2‐yl)palladium(II) (SPhosPdG4, 24 mg, 0.03 mmol, 10 mol%) were added. The vial was evacuated and refilled with argon three times, before 3 mL absolute 1,4‐dioxane were added. The resulting mixture was stirred at 90 °C for 18 h, cooled to RT, and diluted with 5 mL ethyl acetate. The black opaque mixture was filtered through a 1 cm pad of celite, washed with 3 mL of water and 3 mL of brine, dried over Na_2_SO_4_, and filtered. To the filtrate 2 g of silica was added and the volatiles were removed under reduced pressure. The resulting dry load was directly used in a flash column chromatography (25 g silica, ethyl acetate/*n*‐hexane gradient from 0–66%) to afford compounds ***m*** **1** or ***p*** **1**.

##### 2‐[9‐(1,7‐Dicarba‐Closo‐Dodecaboranyl‐2‐Aminopheny)]Acetonitrile (m1)

Off‐white powder. 71% yield (58 mg, 0.22 mmol). mp: 101.0 °C (ethyl acetate/*n*‐hexane). ^1^H NMR (400 MHz, DMSO) δ 7.20–7.08 (m, 3H_Ar_), 6.66 (td, *J* = 7.3, 1.3 Hz, 1H_Ar_), 4.51 (s, 1H_Amine_), 3.92 (s, 2H_Benzyl_), 3.89 (s, 2H_Cluster‐CH_), 3.19–1.08 (m, 9H_Cluster‐BH_). ^11^B{^1^H} NMR (128 MHz, DMSO) δ 2.7 (s, 1B_BN_), −8.1 (s, 2B), −11.7 (s, 1B), −14.4 (s, 2B), −15.9 (s, 2B), −19.1 (s, 1B), −23.4 (s, 1B). ^11^B NMR (128 MHz, DMSO) δ 2.7 (s, 1B_BN_), −8.3 (m, 1B), −9.5–−21.1 (m, 7B), −23.0 (s, 1B). ^13^C{^1^H} NMR (101 MHz, DMSO) δ 146.9 (s, C_CN_) 129.9 (s, C_Ar_), 128.8 (s, C_Ar_), 119.3 (s, C_Ar_), 117.8 (s, C_Ar_), 115.9 (s, C_Ar_), 52.1 (s, C_Cluster_), 19.9 (s, C_Benzyl_). HR‐ESI‐MS (positive mode, ACN) m/z [M + H]^+^: calcd. for C_10_H_19_B_10_N_2_: 275.2551, found: 275.2544 with the expected isotopic distribution.

##### 2‐[2‐(1,12‐Dicarba‐Closo‐Dodecaboranyl‐2‐Aminopheny)]Acetonitrile (p1)

Light yellow powder. 60% yield (49 mg, 0.18 mmol). mp: 93.9 °C (ethyl acetate/*n*‐hexane). ^1^H NMR (400 MHz, DMSO) δ 7.33 (d, *J* = 8.0 Hz, 1H_Ar_), 7.22 (t, *J* = 8.0 Hz, 2H_Ar_), 6.80 (td, *J* = 7.4, 1.2 Hz, 1H_Ar_), 4.97 (s, 1H_Amine_), 3.90 (s, 2H_Benzyl_), 3.87 (s, 1H_Cluster‐CH_), 3.78 (s, 1H_Cluster‐CH_), 2.50 (m, 9H_Cluster‐BH_). ^11^B{^1^H} NMR (128 MHz, DMSO) δ − 2.2 (s, 1B), −16.2 (m, 8B), −21.5 (s, 1B). ^11^B NMR (128 MHz, DMSO) δ − 2.1 (s, 1B), −16.4 (m, 8B), −21.5 (s, 1B). ^13^C{^1^H} NMR (101 MHz, DMSO) δ 146.0 (s, C_CN_), 129.7 (s, C_Ar_), 128.9 (s, C_Ar_), 119.8 (s, C_Ar_), 119.6 (s, C_Ar_), 119.3 (s, C_Ar_), 118.3 (s, C_Ar_), 70.7 (s, C_Cluster_) 62.3 (s, C_Cluster_), 19.0 (s, C_Benzyl_). HR‐ESI‐MS (positive mode, ACN) m/z [M + Na]^+^: calcd. for C_10_H_18_B_10_N_2_Na: 297.2370, found: 297.2375 with the expected isotopic distribution.

##### Synthesis of N‐Methylated Nitriles m2 and p2

In a 10 mL Schlenk tube (flask A, open to air) containing a stir bar 120 μL 37% v/v aqueous formaldehyde solution and 2 mL 3 m H_2_SO_4_ were mixed and cooled to 0 °C. In a second flask (flask B), the respective nitrile ***m*** **1** or ***p*** **1** (40 mg, 0.15 mmol, 1.0 equiv.) and sodium borohydride (23 mg, 0.6 mmol, 4.0 equiv.) were suspended in 2 mL THF. The resulting suspension from flask B was transferred to a syringe (diameter of the canula should be >1.2 mm to prevent clogging) and added dropwise to the THF solution in flask A over 5 min (with visible gas evolution). After addition, the mixture was warmed to RT and left to stir for 10 min. The reaction was quenched by dropwise addition of ≈2 mL 4 m aqueous NaOH until the pH of the mixture was ≈8–9. The mixture was extracted three times with 10 mL ethyl acetate. The combined organic extracts were dried over Na_2_SO_4_ and filtered. To the filtrate 1 g of silica was added and the volatiles were removed under reduced pressure. The resulting dry load was directly used in a flash column chromatography (10 g silica, CH_2_Cl_2_/*n*‐hexane gradient from 0%–60%) to afford compounds ***m*** **2** or ***p*** **2**.

##### 2‐[N‐9‐(1,7‐Dicarba‐Closo‐Dodecaboranyl)‐N‐Methyl‐2‐Aminopheny)]Acetonitrile (m2)

Beige powder. 90% yield (39 mg, 0.14 mmol). mp: 87.3 °C (CH_2_Cl_2_/*n*‐hexane). ^1^H NMR (400 MHz, CDCl_3_) δ 7.54–7.42 (m, 1H_Ar_), 7.33–7.27 (m, 2H_Ar_), 7.17 (td, *J* = 7.2, 2.0 Hz, 1H_Ar_), 3.86 (s, 2H_Benzyl_), 2.89 (s, 3H_Methyl_), 2.71 (s, 2H_Cluster‐CH_), 3.50–1.00 (m, 9H_Cluster‐BH_). ^11^B{^1^H} NMR (128 MHz, CDCl_3_) δ 6.1 (s, 1B_BN_), −8.6 (s, 2B), −11.6 (s, 1B), −14.9 (s, 2B), −15.9 (s, 2B), −19.5 (s, 1B), −24.7 (s, 1B). ^11^B NMR (128 MHz, CDCl_3_) δ 6.1 (s, 1B_BN_), −8.6 (d, *J* = 160.9 Hz, 2B), −11.7 (d, *J* = 150.6 Hz, 1B), −15.4 (t, *J* = 153.8 Hz, 4B), −19.5 (d, *J* = 181.0 Hz, 1B), −24.7 (d, *J* = 181.6 Hz, 1B). ^13^C{^1^H} NMR (101 MHz, CDCl_3_) δ 150.6 (s, C_CN_), 129.4 (s, C_Ar_), 128.9 (s, C_Ar_), 128.9 (s, C_Ar_), 125.4 (s, C_Ar_), 119.1 (s, C_Ar_), 49.9 (s, C_Cluster_), 43.9 (s, C_Benzyl_), 19.8 (s, C_Methyl_). HR‐ESI‐MS (positive mode, ACN) m/z [M + H]^+^: calcd. for C_11_H_21_B_10_N_2_: 289.2708, found: 289.2721 with the expected isotopic distribution.

##### 2‐[N‐(9‐(1,12‐Dicarba‐Closo‐Dodecaboranyl)‐N‐Methyl‐2‐Aminopheny)]Acetonitrile (p2)

Yellow oil. 88% yield (38 mg, 0.13 mmol). ^1^H NMR (400 MHz, DMSO) δ 7.44 (dd, *J* = 7.7, 1.6 Hz, 1H_Ar_), 7.32 (dtd, *J* = 15.6, 7.8, 1.7 Hz, 2H_Ar_), 7.24 (td, *J* = 7.3, 1.7 Hz, 1H_Ar_), 4.00 (s, 1H_Cluster‐CH_), 3.92 (s, 2H_Benzyl_), 3.68 (s, H_Cluster‐CH_), 2.88 (s, 3H_Methyl_), 3.50–1.00 (m, 9H_Cluster‐BH_). ^11^B{^1^H} NMR (128 MHz, DMSO) δ 0.6 (s, 1B_BN_), −16.0 (s, 5B), −17.7 (s, 2B), −21.9 (s, 2B). ^11^B NMR (128 MHz, DMSO) δ 0.6 (s, 1B_BN_), −16.4 (m, 6B), −21.7 (s, 3B). ^13^C{^1^H} NMR (101 MHz, DMSO) δ 150.8 (s, C_CN_), 130.3 (s, C_Ar_), 129.9 (s, C_Ar_), 129.8 (s, C_Ar_), 129.6 (s, C_Ar_), 126.5 (s, C_Ar_), 119.9 (s, C_Ar_), 67.3 (s, C_Cluster_), 62.5 (s, C_Cluster_), 44.2 (s, C_Benzyl_), 19.6 (s, C_Methyl_). HR‐ESI‐MS (positive mode, ACN) m/z [M + Na]^+^: calcd. for C_11_H_20_B_10_N_2_Na: 311.2527, found: 311.2531 with the expected isotopic distribution.

##### One‐Pot Methylation and Hydrolysis

In a 25 mL round‐bottom flask (open to air) containing a stir bar, nitrile ***m*** **1** (or ***m*** **2**) (1.2 mmol, 1.0 equiv.) and sodium borohydride (181 mg, 4.8 mmol, 4.0 equiv.) were suspended in 5 mL THF. The reaction mixture was cooled to 0 °C and 1.5 mL 37% aqueous formaldehyde and 5 mL 40% v/v aqueous H_2_SO_4_ were slowly added via a syringe (with visible gas evolution). After addition, a reflux condenser was connected and the mixture was stirred at 90 °C for 18 h. The mixture was cooled to 0 °C and 10 mL saturated aqueous NaHCO_3_ was added carefully to quench the reaction. When gas evolution stopped, the mixture was extracted three times with 10 mL ethyl acetate. The combined organic extracts were dried over MgSO_4_ and filtered. To the filtrate 4 g of silica were added and the volatiles were removed under reduced pressure. The resulting dry load was directly used in a flash column chromatography (50 g silica, CH_2_Cl_2_/*n*‐hexane gradient from 0%–95%) to afford **3**.

##### 2‐[N‐9‐(1,7‐Dicarba‐Closo‐Dodecaboranyl)‐N‐Methyl‐2‐Aminopheny)]Acetic Acid (3)

Vanilla yellow powder. 56% yield (206 mg, 0.67 mmol). mp: 108.7 °C (CH_2_Cl_2_/*n*‐hexane). ^1^H NMR (400 MHz, DMSO) δ 12.13 (s, 1H_COOH_), 7.21 (ddd, *J* = 16.2, 7.4, 1.7 Hz, 2H_Ar_), 7.14 (dd, *J* = 7.9, 1.5 Hz, 1H_Ar_), 7.06 (td, *J* = 7.3, 1.5 Hz, 1H_Ar_), 3.70 (s, 2H_Cluster‐CH_), 3.60 (m, 2H_Benzyl_), 2.78 (s, 3H_Methyl_), 3.50–1.00 (m, 9H_Cluster‐BH_). ^11^B{^1^H} NMR (128 MHz, DMSO) δ 6.3 (s, 1B_BN_), −9.0 (s, 2B), −12.0 (s, 1B), −14.8 (s, 1B), −16.3 (s, 2B), −19.3 (s, 1B), −24.5 (s, 1B). ^11^B NMR (128 MHz, DMSO) δ 6.3 (s, 1B_BN_), −9.3 (m, 2B), −12.0 (d, *J* = 153.0 Hz, 1B), −16.2 (m, 3B), −19.4 (d, *J* = 176.0 Hz, 1B), −24.4 (m, 1B). ^13^C{^1^H} NMR (101 MHz, DMSO) δ 173.6 (s, C_COOH_), 152.5 (s, C_Ar_), 134.4 (s, C_Ar_), 131.3 (s, C_Ar_), 129.2 (s, C_Ar_), 127.9 (s, C_Ar_), 124.9 (s, C_Ar_), 51.2 (s, C_Cluster_), 44.2 (s, C_Benzyl_), 36.9 (s, C_Methyl_). HR‐ESI‐MS (positive mode, ACN) m/z [M + H]^+^: calcd. for C_11_H_22_B_10_NO_2_: 308.2661, found: 308.2654 with the expected isotopic distribution.

##### Synthesis of Methyl Ester 8

In a 100 mL round‐bottom Schlenk flask covered with a rubber septum and containing a stir bar, anthranilic acid derivative **7** (4.06 g, 14.4 mmol, 1.0 equiv.) was suspended in 40 mL dry methanol. The mixture was cooled to 0 °C and SOCl_2_ (1.88 mL, 3.08 g, 26 mmol, 1.8 equiv.) was added over 5 min via syringe. The septum was exchanged for a stopcock and the mixture was stirred at 60 °C for 18 h. The resulting red brown solution was brought to RT and poured on 50 mL ice water. The methanol was removed under reduced pressure and the resulting brown slurry was extracted three times with 10 mL ethyl acetate. The combined organic extracts were dried over MgSO_4_ and filtered. To the filtrate 4 g of silica were added and the volatiles were removed under reduced pressure. The resulting dry load was directly used in a flash column chromatography (50 g silica, CH_2_Cl_2_/*n*‐hexane gradient from 0%–50%) to afford **8**.

##### Methyl 2‐[(2,6‐Dichlorophenyl)Amino]Benzoate (8)

White powder. 74% yield (3.16 g, 10.67 mmol). mp: 101.3 °C (CH_2_Cl_2_/*n*‐hexane). ^1^H NMR (400 MHz, DMSO) δ 9.25 (s, 1H_Amin_), 7.92 (dd, *J* = 7.9, 1.7 Hz, 1H_Ar_), 7.63 (d, *J* = 8.0 Hz, 2 H_Ar_), 7.38 (q, *J* = 7.4, 6.8 Hz, 2 H_Ar_), 6.86–6.77 (m, 1 H_Ar_), 6.24 (d, *J* = 8.5 Hz, 1 H_Ar_), 3.90 (s, 3 H_OMe_). ^13^C{^1^H} NMR (101 MHz, DMSO) δ 168.6 (s, C_COOMe_), 147.3 (s, C_Ar_), 135.1 (s, C_Ar_), 135.0 (s, C_Ar_), 134.0 (s, C_Ar_), 131.6 (s, C_Ar_), 129.7 (s, C_Ar_), 129.1 (s, C_Ar_), 118.2 (s, C_Ar_), 113.7 (s, C_Ar_), 111.5 (s, C_Ar_), 52.6 (s, C_OMe_). HR‐ESI‐MS (positive mode, ACN) m/z [M + Na]^+^: calcd. for C_14_H_11_Cl_2_NO_2_Na: 318.0059, found: 318.0050 with the expected isotopic distribution.

##### Biological Evaluation: Materials and Methods

The materials used for biological study were Roswell Park Memorial Institute‐1640 (RPMI‐1640) and Dulbecco's modified Eagle medium (DMEM) culture media (both from Capricorn Scientific GmbH, Ebsdorfergrund, Germany); fetal calf serum, phosphate‐buffered saline (PBS), trypsin, dimethyl sulfoxide (DMSO), CV, CFSE, PI (all from Sigma–Aldrich, St. Louis, MO, USA); penicillin/streptomycin solution (Biological Industries, Cromwell, CT, USA); 3‐(4,5‐dimethythiazol‐2‐yl)‐2,5‐diphenyltetrazolium bromide (MTT; AppliChem, St. Louis, MO, USA); dihydrorhodamine 123 (DHR123; Thermo Fisher Scientific, Waltham, MA, USA); paraformaldehyde (PFA; Serva, Heidelberg, Germany); annexin V‐fluorescein isothiocyanate (Ann V‐FITC; BD Pharmingen, San Diego, CA, USA); ApoStat (R&D Systems, Minneapolis, MN, USA); AO (LaboModerne, Paris, France).

DMSO was used for the preparation of the compound stocks to achieve a concentration of 100 mM. The stock solutions were stored at –20 °C for up to 14 days. Working solutions were freshly prepared from the stock solutions by further dissolution in a culture medium immediately prior to treatment.

A murine colon adenocarcinoma cell line (MC38), two human colorectal carcinoma cell lines (HCT116 and HT29), and mouse embryonic fibroblasts (NIH/3T3) were used for the biological study. All cell lines were obtained from the American Type Culture Collection (Rockville, MD, USA). The HCT116 and HT29 cell lines were cultivated in 4‐(2‐hydroxyethyl)‐1‐piperazineethanesulfonic acid (HEPES) buffered RPMI‐1640 medium, while the MC38 and NIH/3T3 cell lines were maintained in DMEM medium. Both media were previously supplemented with 10% heat‐inactivated FBS, 2 mM L‐glutamine, 0.01% sodium pyruvate, and antibiotics (penicillin (100 units mL^−1^), and streptomycin (100 μg mL^−1^)). For the cultivation of the MC38 cell line, nonessential amino acids were also added to the DMEM medium at a final concentration of 1%. All cells were grown under standard conditions at 37 °C in a humidified atmosphere with 5% CO_2_.

For the viability tests, the density of MC38, HCT116, and HT29 cells in 96‐well plates was 2 × 10^3^, 5 × 10^3^, and 6 × 10^3^ cells/well, respectively. To determine whether the drug affected the viability of healthy, nonmalignant cells, NIH/3T3 were seeded at a density of 7 × 10^3^ in 96‐well plates. MC38 cells were seeded in six‐well plates at 5 × 10^4^ cells/well for the flow cytometric analyzes. To observe morphological changes of nuclei and to detect autophagy, MC38 cells were seeded in two‐well chamber slides at 1.5 × 10^4^ cells/well.

##### Viability Assays

Colorimetric assays, MTT, and CV were used to evaluate the cytotoxic effect of the compounds on cell viability. All cell lines were exposed to a wide range of concentrations (1.56–100 μM) of the experimental compounds for 72 h. Afterward, cells were incubated with an MTT solution (0.5 mg mL^−1^) for 30 min and the formazan crystals formed were dissolved by adding DMSO. To perform the CV assay, the cells were fixed with 4% PFA for 10 min and then stained with a 0.02% CV solution for 15 min at RT. Eventually, the dye was dissolved in 33% acetic acid. For both viability assays, absorbance was measured using an automated microplate reader at 540/670 nm, with results expressed as a percentage of untreated cells (control), arbitrarily set at 100%. IC_50_ values were calculated using a four‐parameter logistic function and presented as mean ± SD. All experiments were performed three times.

To define the role of autophagy, a concomitant treatment with the autophagy inhibitor chloroquine (Chlq) and compounds ***m*** **1** or **11** was performed. MC38 cells were seeded overnight and treated simultaneously with IC_50_ concentrations of experimental compounds and Chlq (20 μM). After 72 h, cell viability was determined using a viability assay.

##### Flow Cytometric Analyzes

For all flow cytometric analyzes, MC38 cells were seeded overnight in six‐well plates and then treated with the IC_50_ concentrations of compounds ***m*** **1**, ***m*** **2**, and **11** for 72 h. Cells were trypsinized, washed, and stained as per the instructions provided by the manufacturer for each method. Cells were double stained with AnnV‐FITC and PI (15 μg mL^−1^) for 15 min at RT, protected from light, to determine whether the tested compounds induced apoptosis. The FITC‐conjugated caspase inhibitor ApoStat was used to detect total caspase activity. After treatment, cells were stained with it for 30 min at 37 °C. For detection of acidic vesicles, autophagosomes, cells were stained with a 10 μM solution of AO for 15 min at 37 °C. Before seeding, MC38 cells were prestained with a CFSE solution (1 μM) for 10 min at 37 °C and with the redox‐sensitive dye DHR 123 (1 μM) for 20 min at 37 °C, to detect cell proliferation and intracellular production of ROS/RNS, respectively. The cells were then seeded and treated as described above. Upon 72 h of incubation, all samples were washed, resuspended in PBS, and fluorescence intensity was analyzed on a CytoFLEX flow cytometer (Beckman Coulter, Pasadena, CA, USA). The flow cytometry data were analyzed using FlowJo software.

##### Fluorescence Microscopy

Fluorescence microscopy analysis was performed on MC38 cells seeded in two‐well chamber slides and treated with IC_50_ concentrations of compounds ***m*** **1**, ***m*** **2**, and **11** for 72 h. To detect autophagosomes, cells were incubated with 10 μM AO solution (in PBS) at 37 °C for 15 min after the treatment. The dye was then removed, the cells were washed with PBS and immediately examined under a Leica DM4 B microscope with a DFC7000 T camera (Leica Microsystems CMS GmbH, Wetzlar, Germany) at 400 × magnification. To assess morphological signs of apoptosis, cells were fixed with 4% PFA for 15 min at RT and then stained with PI solution (0.1% Triton X‐100, 0.5 m EDTA pH 8, 50 μg mL^−1^ RNase, and 50 μg mL^−1^ PI, final concentrations in PBS) for 1–2 min at RT. Finally, the fluorescent mounting medium was added (Fluoromount‐G, eBioscience, San Diego, CA, USA) and the slides were observed with a ZeissAxio Observer Z1 inverted fluorescence microscope (Carl Zeiss AG, Oberkochen, Germany) at 400 × magnification.

##### Statistical Analysis

Data were presented as mean ± SD of at least three independent experiments. Differences between groups were analyzed using Student's t‐test. p‐values of less than 0.05 were considered statistically significant.

##### Molecular Docking

The molecular docking was performed with the AutoDockTools4 software using the Lamarckian Genetic Algorithm.^[^ ^47^ ^]^ The partial charges for structures ***m*** **1**, ***p*** **1**, ***m*** **2**, and ***p*** **2** were obtained using HF‐3c implemented in ORCA.^[^ ^48^ ^]^ The structures of ***m*** **2** and ***p*** **2** were not available from X‐ray diffraction (XRD) data; therefore, preliminary structures were created using Avogadro and optimized by semiempirical method PBEh‐3c suggested by ORCA.^[^ ^48^ ^]^ The water molecules were eliminated, and the nonpolar hydrogen atoms were merged. The docking area was limited by the constructed grid box of the size 40 × 40 × 40 centered at 109.572, 51.688, 67.892 of *x*,*y*,*z*‐coordinates (based on the position of the carborane derivative reported in the crystal structure 4Z0L).^[^ ^37^ ^]^ The following parameters were used in the docking: number of hybrid GA‐LS runs: 100; population size: 150; maximum number of energy evaluations: 2,500,000, maximum number of top individuals to survive to next generation: 1; rate of gene mutation: 0.02; rate of crossover: 0.8; mean of Cauchy distribution for gene mutation: 0.0; variance of Cauchy distribution for gene mutation: 1.0.

## Supporting Information

The authors have cited additional references within the Supporting Information.^[^ ^49^ ^]^

## Conflict of Interest

The authors declare no conflict of interest.

## Author Contributions

**Christoph Selg** and **Evamarie Hey‐Hawkins** designed the experiments. **Christoph Selg** and **Robert Schuster** performed the synthesis and characterization of all compounds. **Aleksandr Kazimir** performed all docking studies. **Peter Lönnecke** conducted all X‐ray structure determinations. **Markus Laube**, **Jens Pietzsch**, and **Jonas Schädlich** conducted the COX inhibition studies. **Vuk Gordić**, **Tamara Krajnović**, **Sanja Mijatović**, and **Danijela Maksimović‐Ivanić** conducted all biological studies other than COX inhibition studies. **Mara Wolniewicz** and **Christoph Selg** performed HPLC measurements. **Christoph Selg** and **Evamarie Hey‐Hawkins** wrote the manuscript with contributions from all authors. All authors reviewed the article.

## Supporting information

Supplementary Material

## Data Availability

The data that support the findings of this study are available in the supplementary material of this article. Deposition Numbers 2411971 (for m1), 2411972 (for p1) contain the supplementary crystallographic data for this paper. These data are provided free of charge by the joint Cambridge Crystallographic Data Centre and Fachinformationszentrum Karlsruhe Access Structures service.
