# Supplementary material for: Advances in Diclofenac Derivatives: Exploring Carborane‐Substituted N‐Methyl and Nitrile Analogs for Anticancer Therapy
Source: ChemMedChem. 2025 Apr 14;20(11):e202500084. doi: 10.1002/cmdc.202500084 (PMC12132914; doi:10.1002/cmdc.202500084)
Supplement: Supplementary file 1 — Supplementary Material [file CMDC-20-e202500084-s001.pdf]

Advances in Diclofenac Derivatives: Exploring Carborane-Substituted *N*-Methyl and Nitrile Analogs for Anti-Cancer Therapy

Christoph Selg,[a] Robert Schuster,[a] Aleksandr Kazimir,[b] Peter Lönnecke,[a] Mara Wolniewicz,[c] Jonas Schädlich,[d,e] Markus Laube,[d] Jens Pietzsch,[d,e] Vuk Gordić,[f] Tamara Krajnović,[f] Sanja Mijatović,[f] Danijela Maksimović-Ivanić,[f] Evamarie Hey-Hawkins*[a,g]

[a] Department of Chemistry and Mineralogy, Institute of Bioanalytical Chemistry, Leipzig University, Deutscher Platz 5, 04103 Leipzig, Germany.

[b] Institute for Drug Discovery, Leipzig University, Brüderstraße 34, 04103, Leipzig, Germany

[c] Faculty of Chemistry and Mineralogy, Institute of Organic Chemistry, Leipzig University, Johannisallee 29, 04103 Leipzig, Germany.

[d] Department of Radiopharmaceutical and Chemical Biology, Institute of Radiopharmaceutical Cancer Research, Helmholtz-Zentrum Dresden Rossendorf (HZDR), Bautzner Landstraße 400, 01328 Dresden, Germany.

[e] Faculty of Chemistry and Food Chemistry, School of Science, Technische Universität Dresden, Mommsenstraße 4, 01069 Dresden, Germany

[f] Department of Immunology, Institute for Biological Research "Siniša Stanković", National Institute of the Republic of Serbia, University of Belgrade, 11108 Belgrade, Serbia.

[g] Department of Chemistry, Babeș-Bolyai University, Str. Arany Janos Nr. 11, RO-400028 Cluj-Napoca, Romania.

E-mail: hey@uni-leipzig.de

Supporting Information

Table of Contents

1 Stability Tests for Compounds ***m*1**, ***m*2** and **11** 2

2 Biological Evaluation 2

3 Single Crystal X-ray Diffraction 7

4 NMR Data for Compounds ***m*1**, ***p*1**, ***m*2**, ***p*2**, **8** and **11** 14

5 HPLC Data for compounds ***m*1**, ***p*1**, ***m*2**, ***p*2**, **3**, **7**, **8** and **11** 25

1 Stability Tests for Compounds *m*1, *m*2 and 11

In a 1 mL Eppendorf vial, 10 µL (0.05 µmol) of a freshly prepared 5 mM stock solution of compounds ***m*1**, ***m*2** and **11** in DMSO was mixed with 490 µL of DMEM (Dulbecco's Modified Eagle Medium) containing 10% of fetal bovine serum and stored in an incubation oven at 40 °C for a total 72 hours. After 0, 24 and 72 hours, a 50 µL sample was collected from the mixture and diluted with 250 µL acetonitrile in a 500 µL Eppendorf vial to precipitate the proteins. The mixture was vortexed for 30 s before it was centrifuged for 20 min at 15,000 min^{-1} and 4 °C. 100 µL of the supernatant solution was transferred into a HPLC vial with a pipette and the vial was screw-sealed with a rubber septum. 5 µL of the solution were injected into the HPLC-MS system. The chromatograms after 0, 24 and 72 hours showed no signs of decomposition.

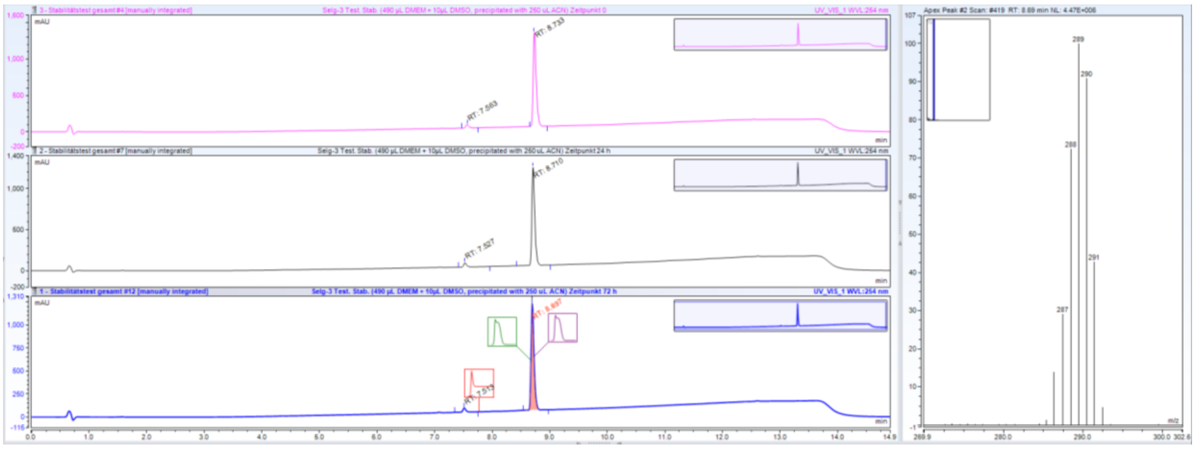

*Figure S 1. Chromatograms to monitor the stability of compound **m1** in the culture medium. Samples measured at 0, 24 and 72 hours (top to bottom) including the mass spectrum (right).*

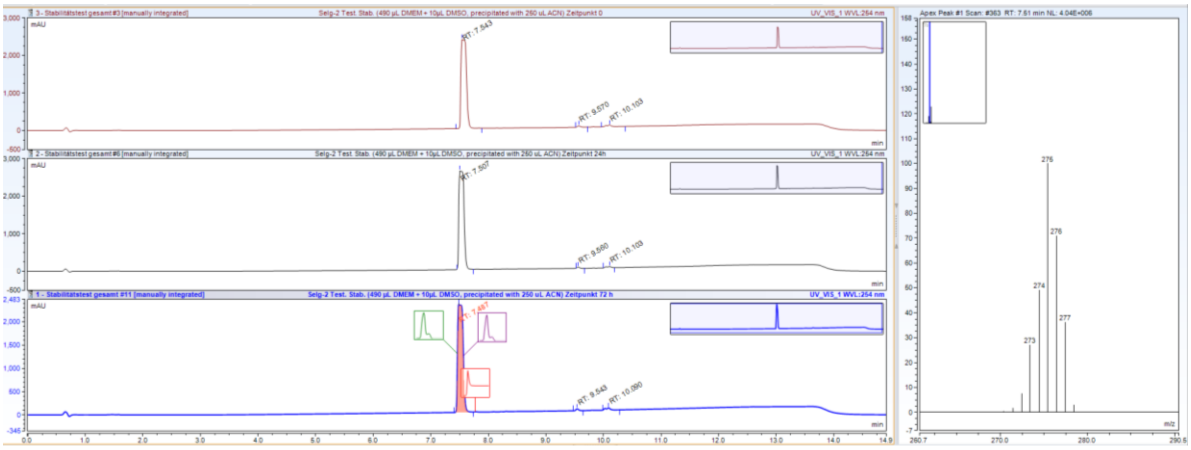

*Figure S 2. Chromatograms to monitor the stability of compound **m2** in the culture medium. Samples measured at 0, 24 and 72 hours (top to bottom) including the mass spectrum (right).*

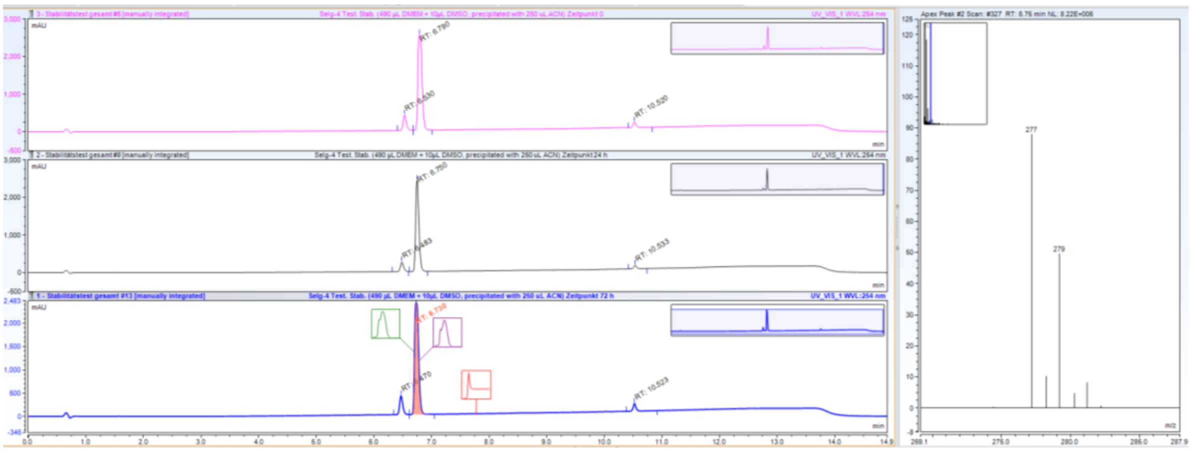

*Figure S 3. Chromatograms to monitor the stability of compound **11** in the culture medium. Samples measured at 0, 24 and 72 hours (top to bottom) including the mass spectrum (right).*

2 Biological Evaluation

COX inhibition assay

The COX inhibition activity against ovine COX-1 and human recombinant COX-2 was determined using the COX Fluorescent Inhibitor Screening Assay Kit (Cayman Chemical Company, Ann Arbor, MI, USA) according to the manufacturer's instructions as reported.[1] Compounds ***m*1**, ***p*1**, ***m*2**, ***p*2**, **3**, **7**, **8**, **11** and **17** were screened at a concentration of 100 µM in duplicate. COX-2 selective inhibitor celecoxib (0.0078 µM–1 µM) and COX-1 selective inhibitor SC-560 (0.0005 µM–1 µM) served as references and were assayed in the concentrations given in parentheses (Table S 1).

Table S 1. COX inhibition potential of references celecoxib and SC-560. Data are presented as mean±SD of three independent measurements.

#	IC_{50} [µM]		pIC_{50}	
	COX-1	COX-2	COX-1	COX-2
**Celecoxib**	-[a]	0.056±0.010	-	7.25±0.08
**SC-560**	0.012±0.002	-	7.91±0.08	-

[a] not determined.

[1] Laube, M. et al. *J. Org. Chem.* **2015**, *80* (11), 5611–5624.

Cell viability

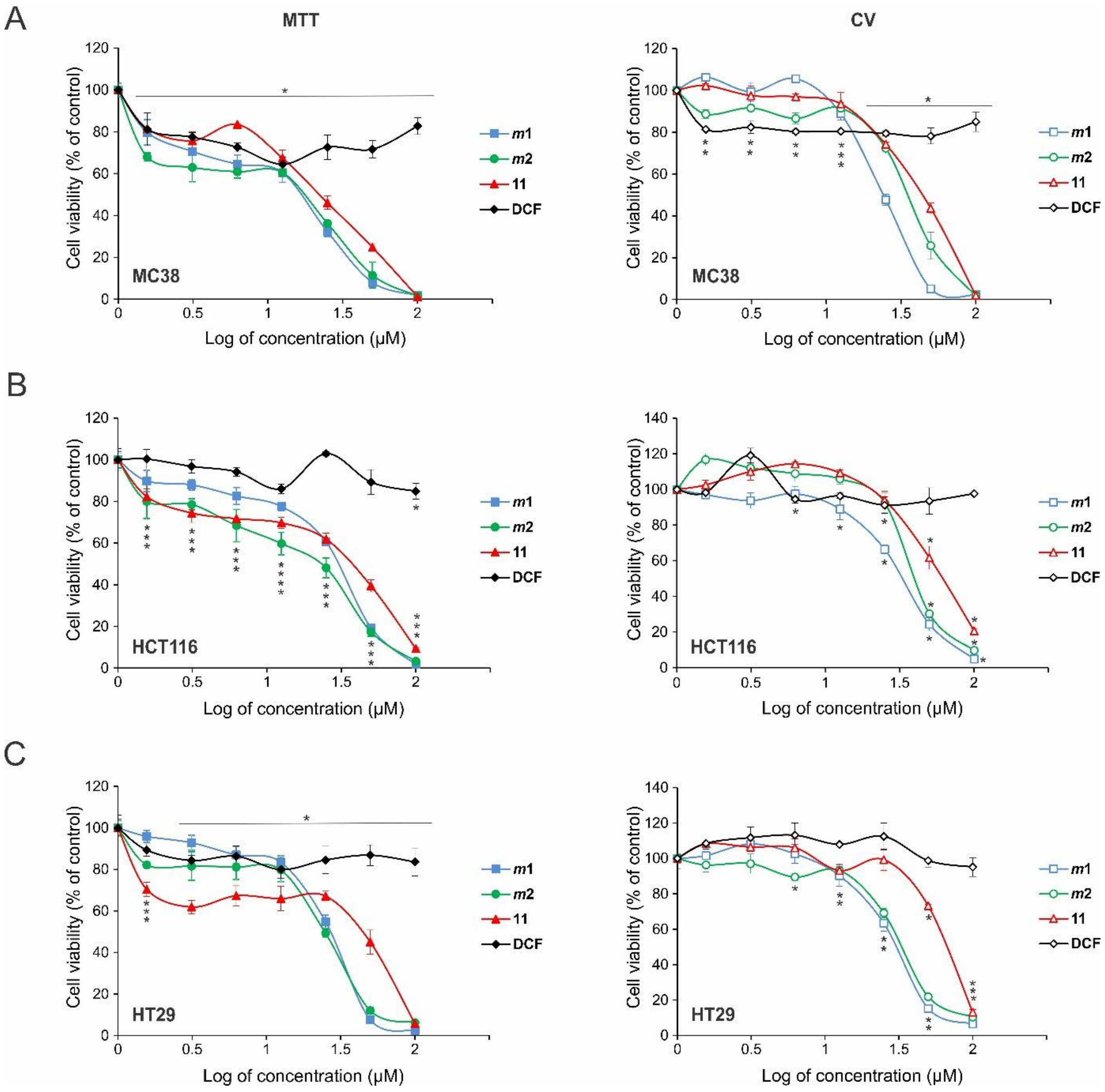

*Figure S 4. Viability of colon cancer cell lines MC38 (A), HCT116 (B) and HT29 (C) treated for 72 h with a wide range of concentrations (1.56 – 100 μM) of **m1**, **m2**, **11**, and **DCF** was determined by MTT (left panel) and CV (right panel) tests. Viability is expressed as a percentage of the absorbance value of control cells that was arbitrarily assigned a viability value of 100%. Results represent SV ± SD of one representative of three independent experiments. * $p < 0.05$ compared to the control.*

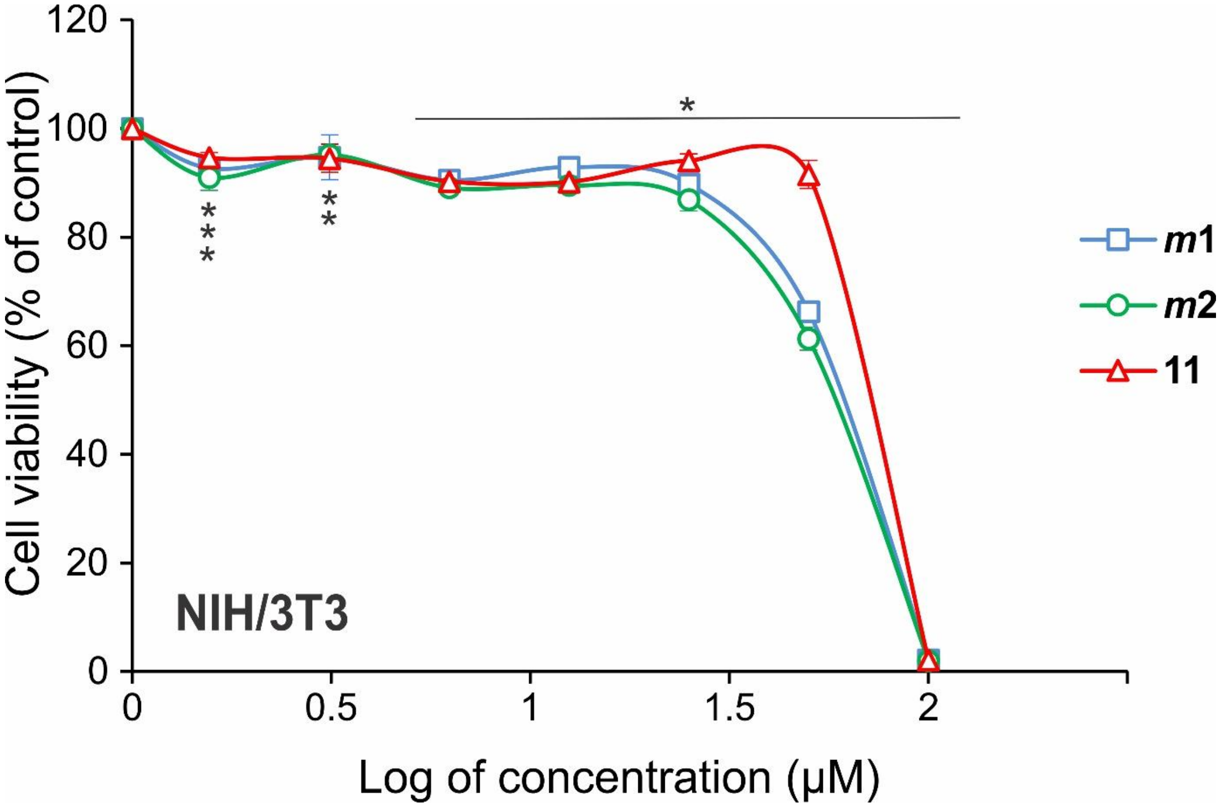

Figure S 5. Viability of the mouse embryonic fibroblast cell line NIH/3T3 treated for 72 h with a wide range of concentrations (1.56 – 100 μM) of ***m1****,* ***m2****, and* ***11*** *was determined by CV assay. Viability is expressed as a percentage of the absorbance value of control cells that was arbitrarily assigned a viability value of 100%. Results represent SV ± SD of one representative of three independent experiments. * p < 0.05 compared to the control.*

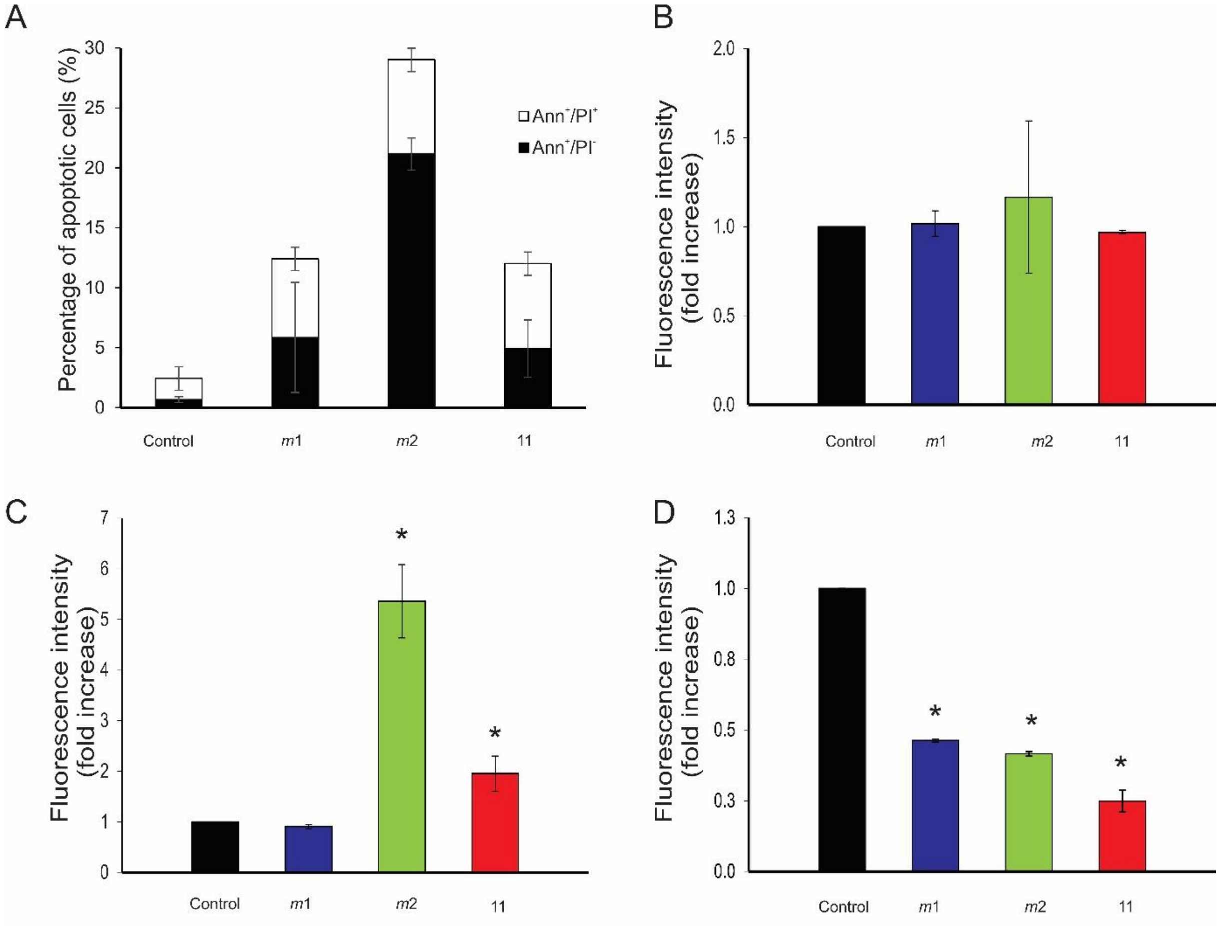

*Figure S 6: The mechanism of action of compounds **m1**, **m2**, and **11** on MC38 cells treated with IC50 concentration of the experimental compounds for 72 h was evaluated by flow cytometry after the corresponding staining: AnnV-FITC/PI (A), ApoStat (B), CFSE (C), and DHR 123 (D). Average + SD from three repeated experiments are shown.*

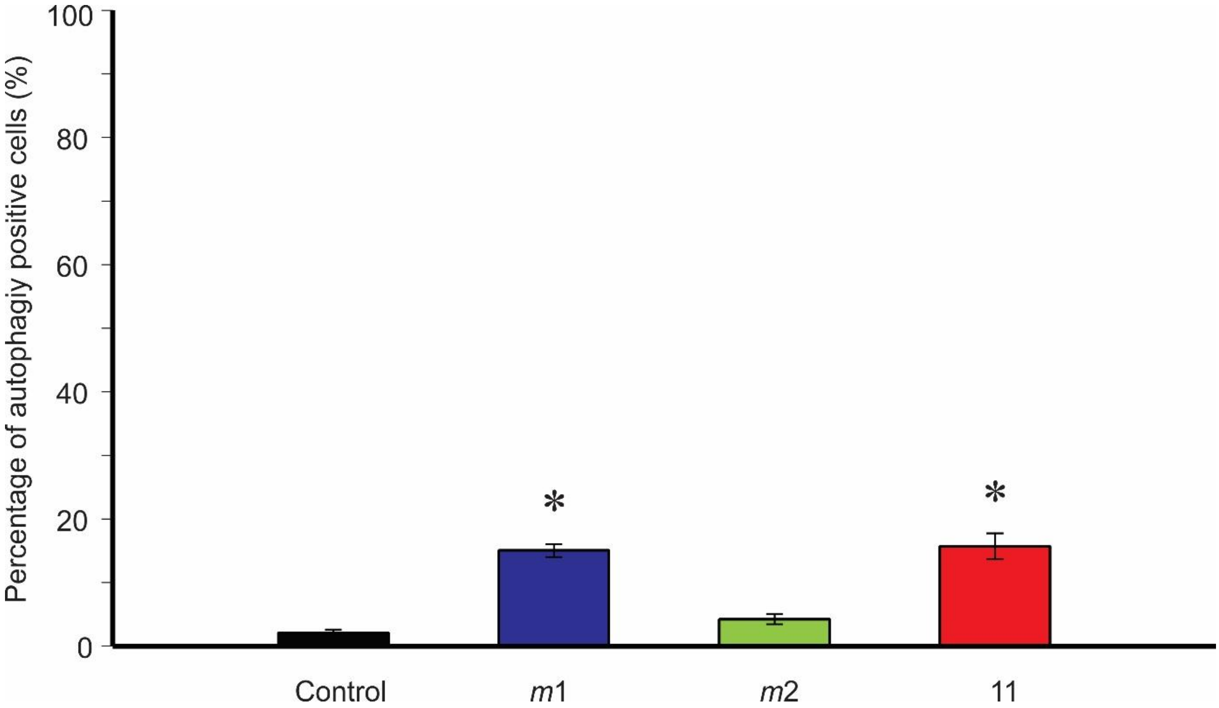

Figure S 7: Autophagy detection. MC38 cells were exposed to IC50 concentrations of compounds ***m1***, ***m2***, *and* ***11*** *for 72 h followed by the AO staining. Average + SD from three repeated experiments are shown.*

3 Single Crystal X-ray Diffraction

The data were collected on a Gemini diffractometer (Rigaku Oxford Diffraction) using Mo-Kα radiation and ω-scan rotation. Data reduction was performed with CrysAlisPro[2] including the program SCALE3 ABSPACK for an empirical absorption correction. All structures were solved by dual space methods with SHELXT[3] and the refinement was performed with SHELXL[4]. All hydrogen atoms are located with a difference-density Fourier map. Carbaborane carbon atoms could be localized for all compounds with a bond length and displacement parameter analysis. Structure figures were generated with DIAMOND-4.[5]

CCDC deposition numbers given in Table S 2 and Table S 3 contain the supplementary crystallographic data for this paper. These data can be obtained free of charge via https://summary.ccdc.cam.ac.uk/structure-summary-form (or from the Cambridge Crystallographic Data Centre, 12 Union Road, Cambridge CB2 1EZ, UK; fax: (+44)1223-336-033; or deposit@ccdc.cam.uk).

[2] Rigaku Corporation. CrysAlisPro Software System. Rigaku Oxford Diffraction; Wroclaw, Poland: 1995–2023.
[3] Sheldrick G.M. SHELXT–integrated space-group and crystal-structure determination. Acta Crystallogr. A Found. Adv. 2015;71:3–8.
[4] Sheldrick G.M. Crystal structure refinement with SHELXL. Acta Crystallogr. C Struct. Chem. 2015;71:3–8.
[5] Crystal Impact GbR. Brandenburg, K; Bonn, Germany: version 4.6.8. DIAMOND 4.

Table S 2. Crystal data and structure refinement for ***m1****.*

Empirical formula	$C_{10}H_{18}B_{10}N_2$	
Formula weight	274.36	
Temperature	130(2) K	
Wavelength	71.073 pm	
Crystal system	Monoclinic	
Space group	P c	
Unit cell dimensions	a = 1510.00(2) pm	a= 90°.
	b = 725.600(10) pm	b= 90.3630(10)°.
	c = 2729.56(4) pm	g = 90°.
Volume	2.99060(7) nm^3	
Z	8	
Density (calculated)	1.219 Mg/m^3	
Absorption coefficient	0.062 mm^{-1}	
F(000)	1136	
Crystal size	0.35 x 0.30 x 0.25 mm^3	
Theta range for data collection	2.698 to 30.352°.	
Index ranges	-20<=h<=21, -10<=k<=10, -37<=l<=35	
Reflections collected	31694	
Independent reflections	13424 [R(int) = 0.0427]	
Completeness to theta = 28.285°	99.9 %	
Absorption correction	Semi-empirical from equivalents	
Max. and min. transmission	1.00000 and 0.99648	
Refinement method	Full-matrix least-squares on F^2	
Data / restraints / parameters	13424 / 2 / 1081	
Goodness-of-fit on F^2	1.030	
Final R indices [I>2sigma(I)]	R1 = 0.0487, wR2 = 0.0939	
R indices (all data)	R1 = 0.0636, wR2 = 0.1008	
Absolute structure parameter	1.5(8)	
Largest diff. peak and hole	0.169 and -0.215 e.Å^{-3}	
CCDC deposition number	2411971	

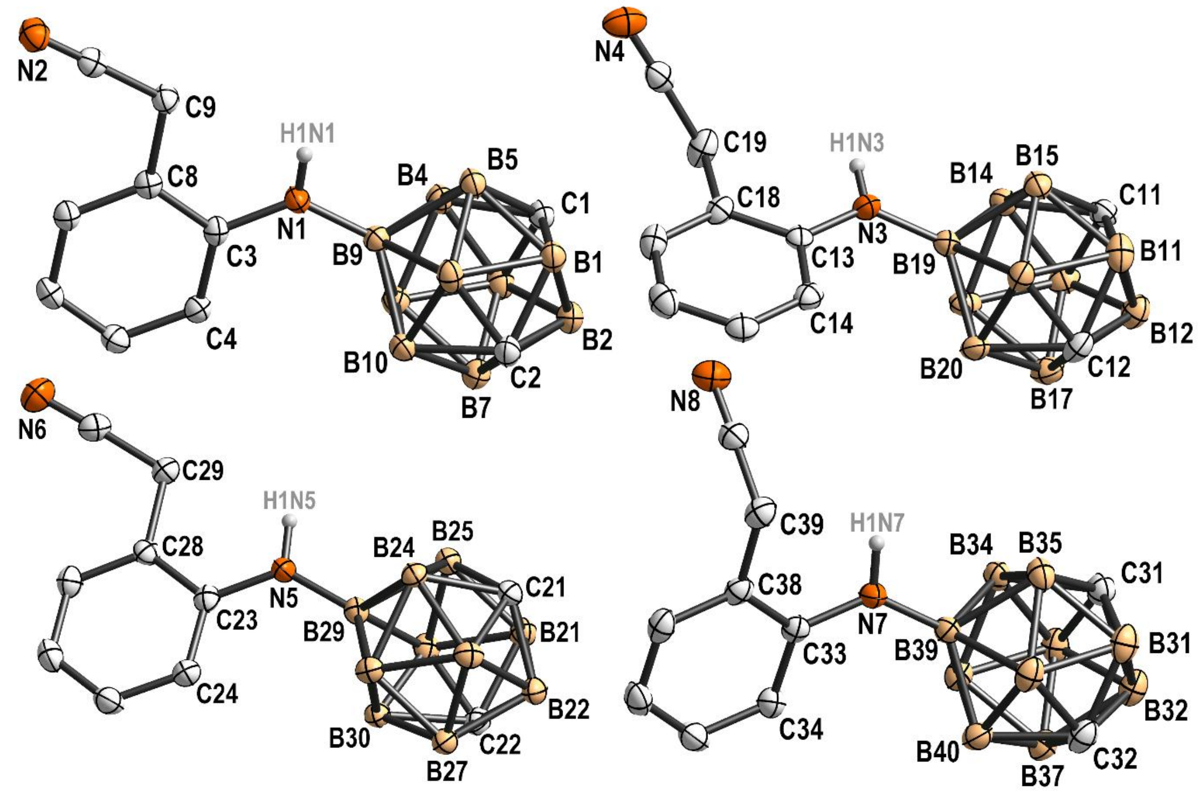

*Figure S 8. Molecular structure and labelling scheme of **m1**. Hydrogen atoms, except NH, were omitted for clarity. Displacement ellipsoids are drawn at the 50% probability level.*

*Table S 3. Crystal data and structure refinement for **p1**.*

Empirical formula	$C_{10}H_{18}B_{10}N_2$	
Formula weight	274.36	
Temperature	130(2) K	
Wavelength	71.073 pm	
Crystal system	Triclinic	
Space group	P -1	
Unit cell dimensions	a = 1017.86(3) pm	a= 98.295(3)°.
	b = 1151.59(4) pm	b= 93.861(3)°.
	c = 1360.73(5) pm	g = 102.082(3)°.
Volume	1.53548(9) nm^3	
Z	4	
Density (calculated)	1.187 Mg/m^3	
Absorption coefficient	0.060 mm^{-1}	
F(000)	568	
Crystal size	0.38 x 0.34 x 0.17 mm^3	
Theta range for data collection	2.431 to 32.482°.	
Index ranges	-14<=h<=14, -14<=k<=17, -20<=l<=19	
Reflections collected	17981	
Independent reflections	9954 [R(int) = 0.0260]	
Completeness to theta = 30.510°	99.9 %	
Absorption correction	Semi-empirical from equivalents	
Max. and min. transmission	1.00000 and 0.99093	
Refinement method	Full-matrix least-squares on F^2	
Data / restraints / parameters	9954 / 0 / 541	
Goodness-of-fit on F^2	1.020	
Final R indices [I>2sigma(I)]	R1 = 0.0501, wR2 = 0.1165	
R indices (all data)	R1 = 0.0726, wR2 = 0.1301	
Largest diff. peak and hole	0.342 and -0.211 e.$Å^{-3}$	
CCDC deposition number	2411972	

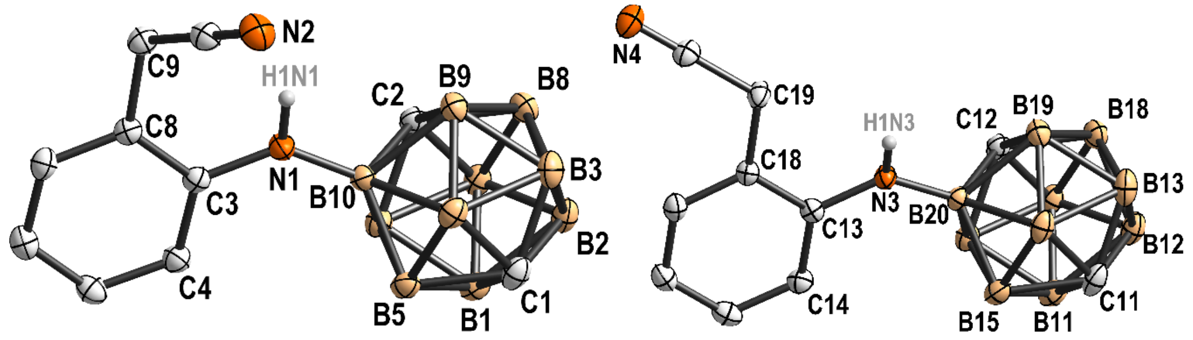

Figure S 9. Molecular structure and labelling scheme of ***p1****. Hydrogen atoms, except NH, were omitted for clarity. Displacement ellipsoids are drawn at the 50% probability level.*

4 NMR Data for Compounds *m*1, *p*1, *m*2, *p*2, 8 and 11

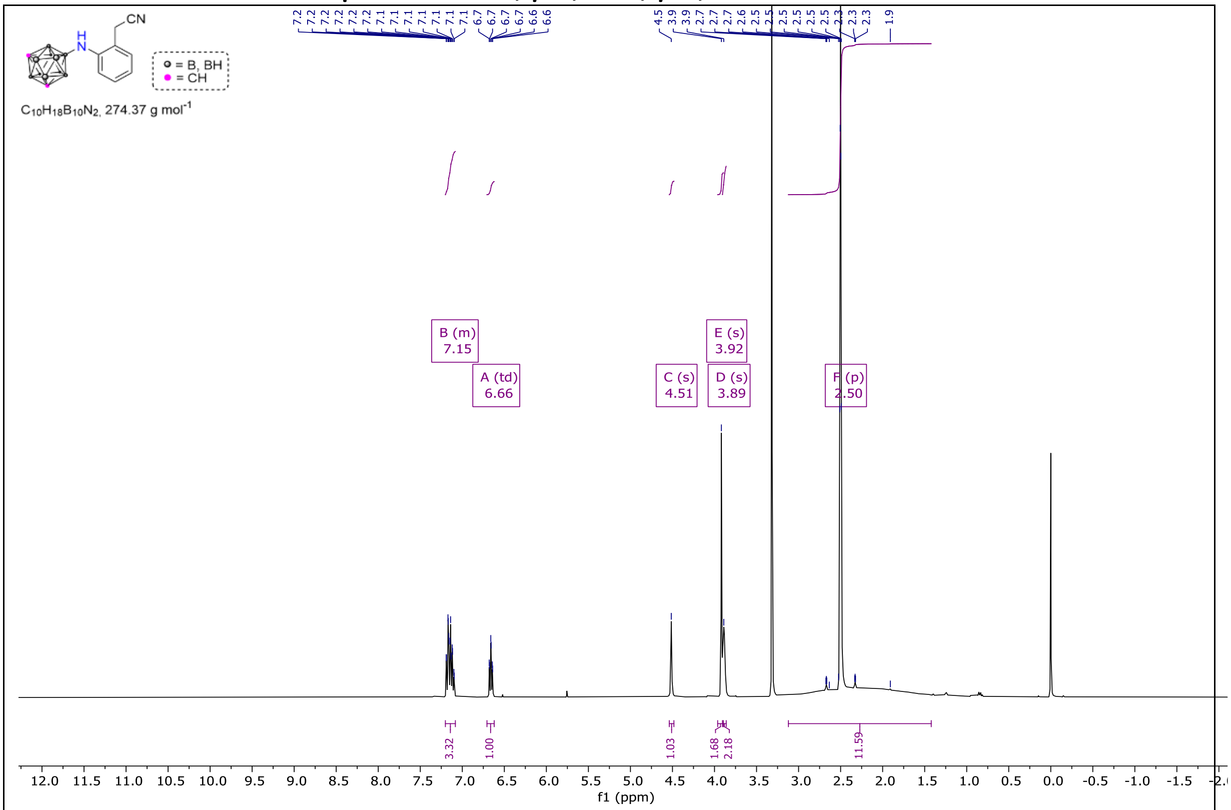

*Figure S 10. ^{1}H NMR spectrum of **m1** in DMSO-d_6.*

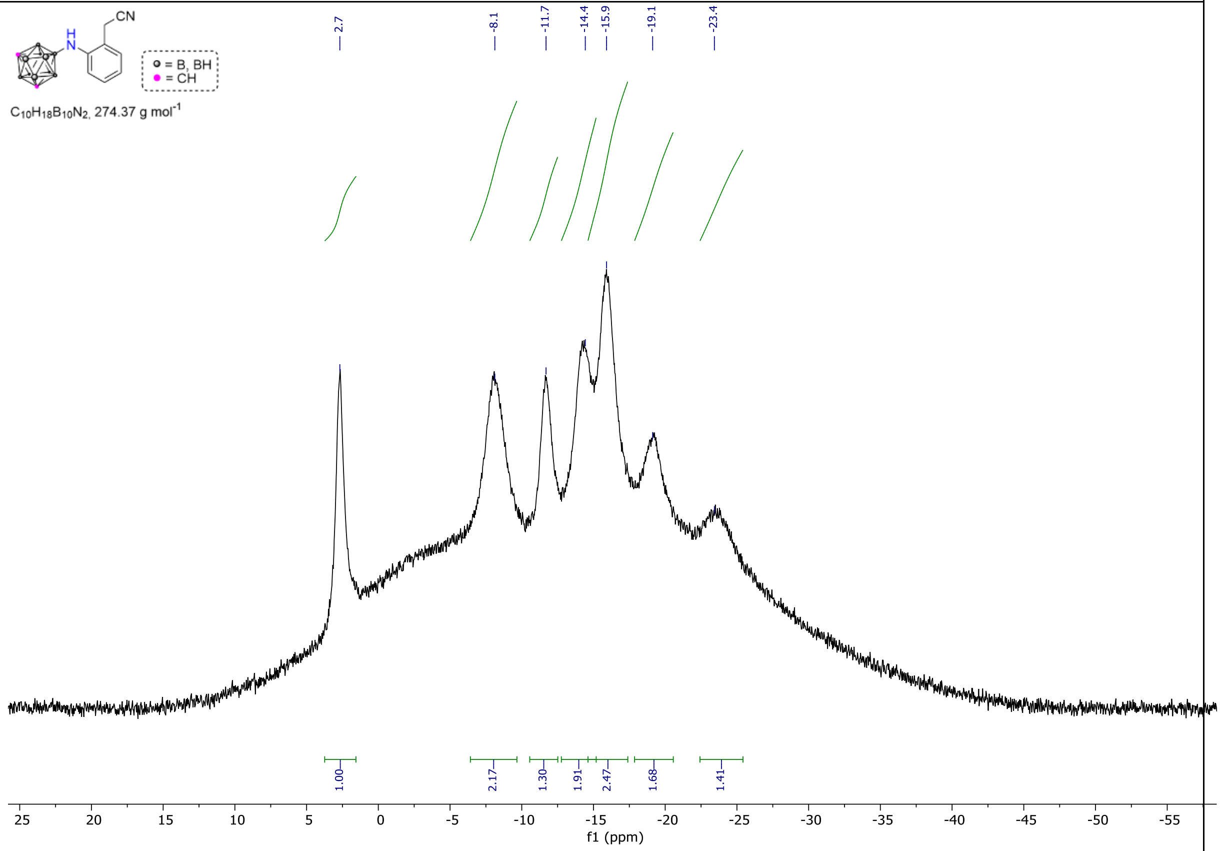

*Figure S 11. $^{11}B\{^{1}H\}$ NMR spectrum of **m1** in DMSO-d_6.*

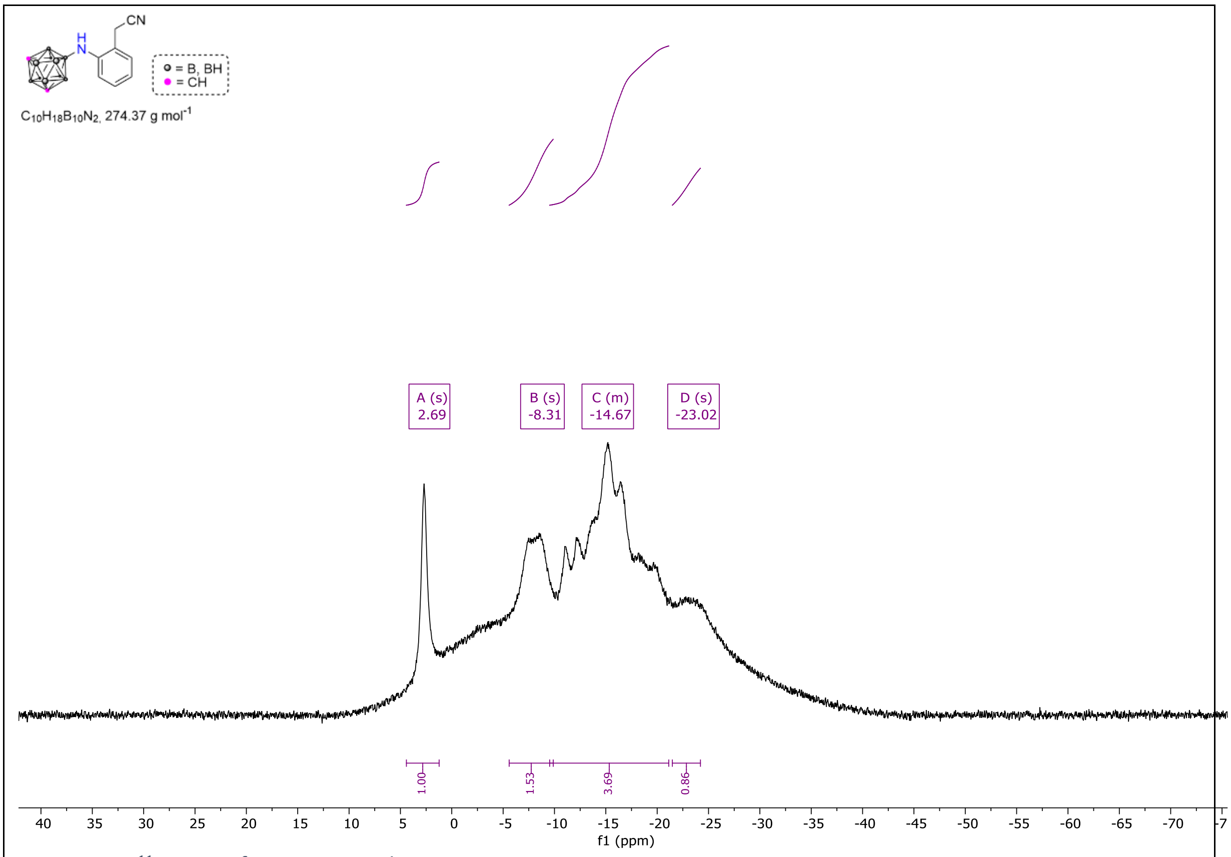

Figure S 12. ^{11}*B NMR of* ***m1*** *in DMSO-d*$_6$.

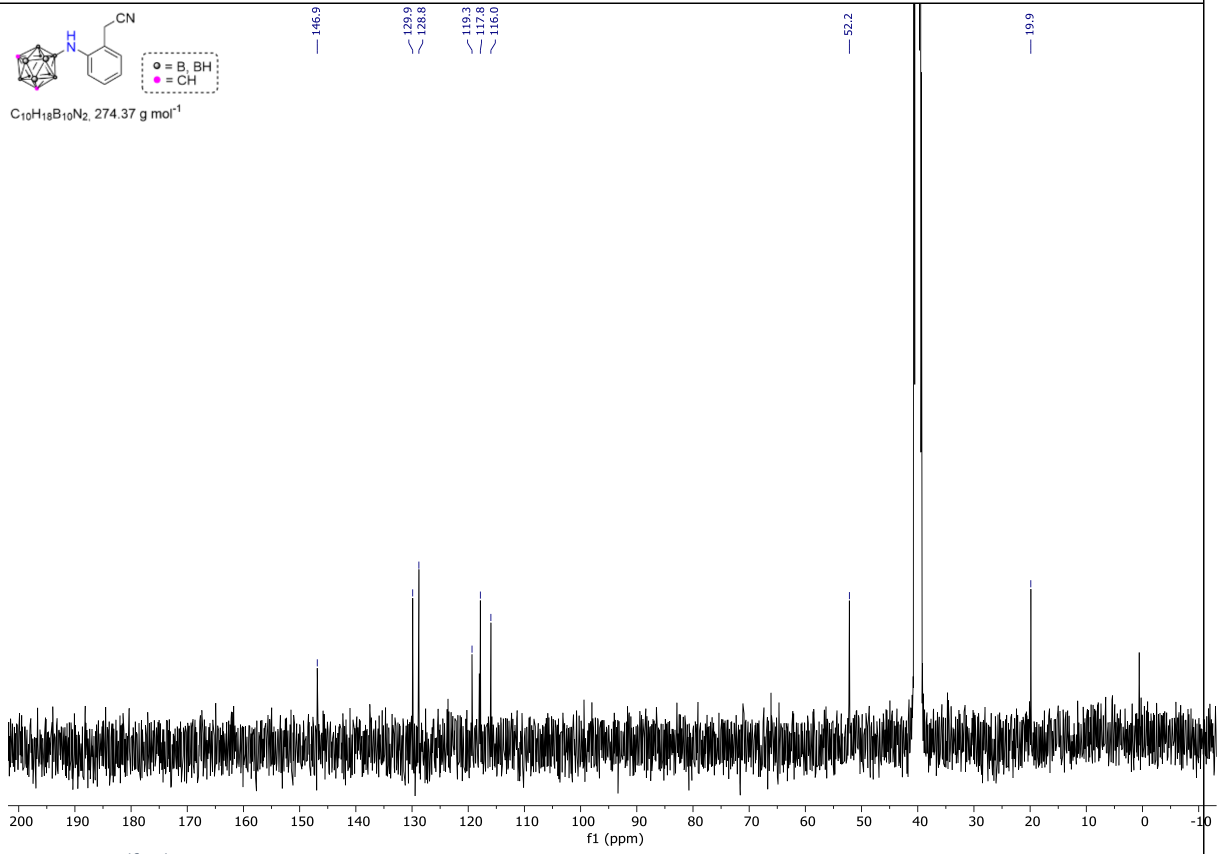

Figure S 13. ^{13}*C{*^{1}*H} NMR spectrum of* ***m1*** *in DMSO-d*$_6$.

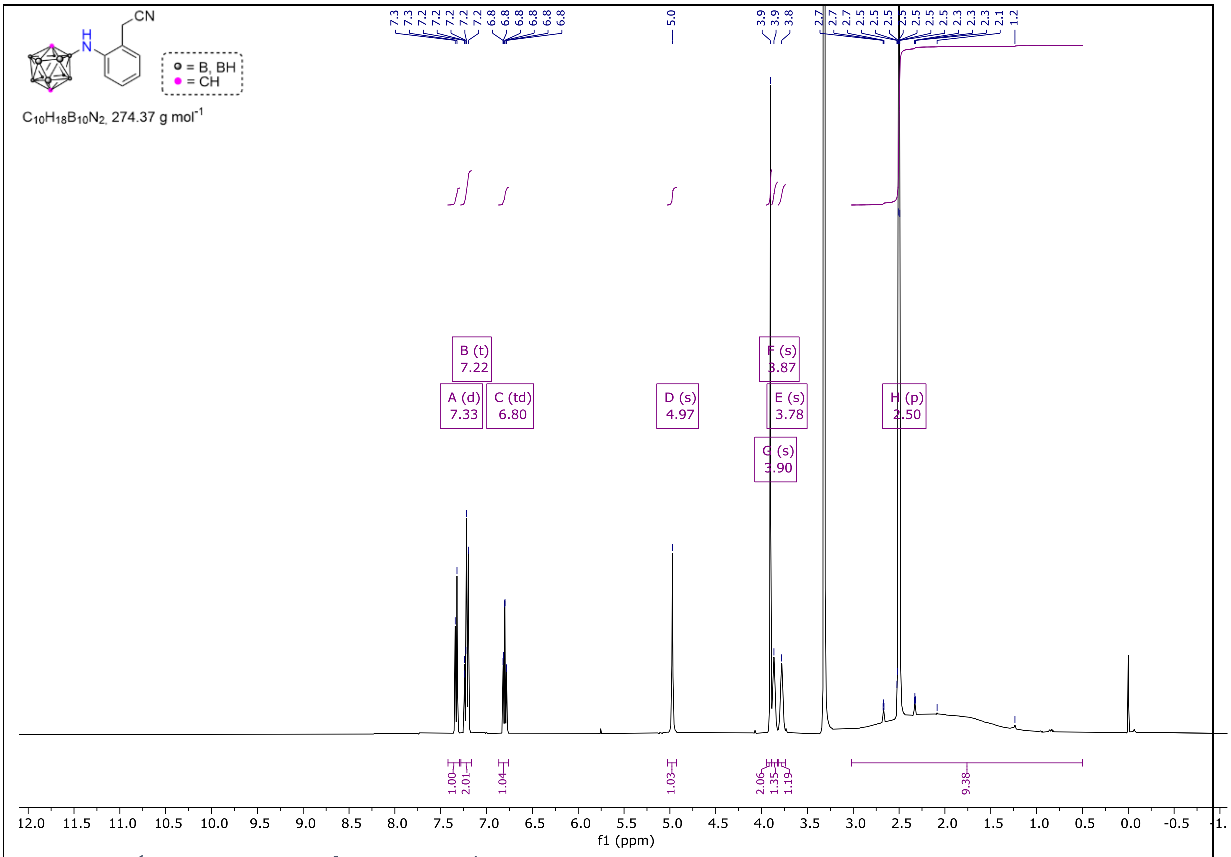

*Figure S 14. ^{1}H NMR spectrum of **p1** in DMSO-d_6.*

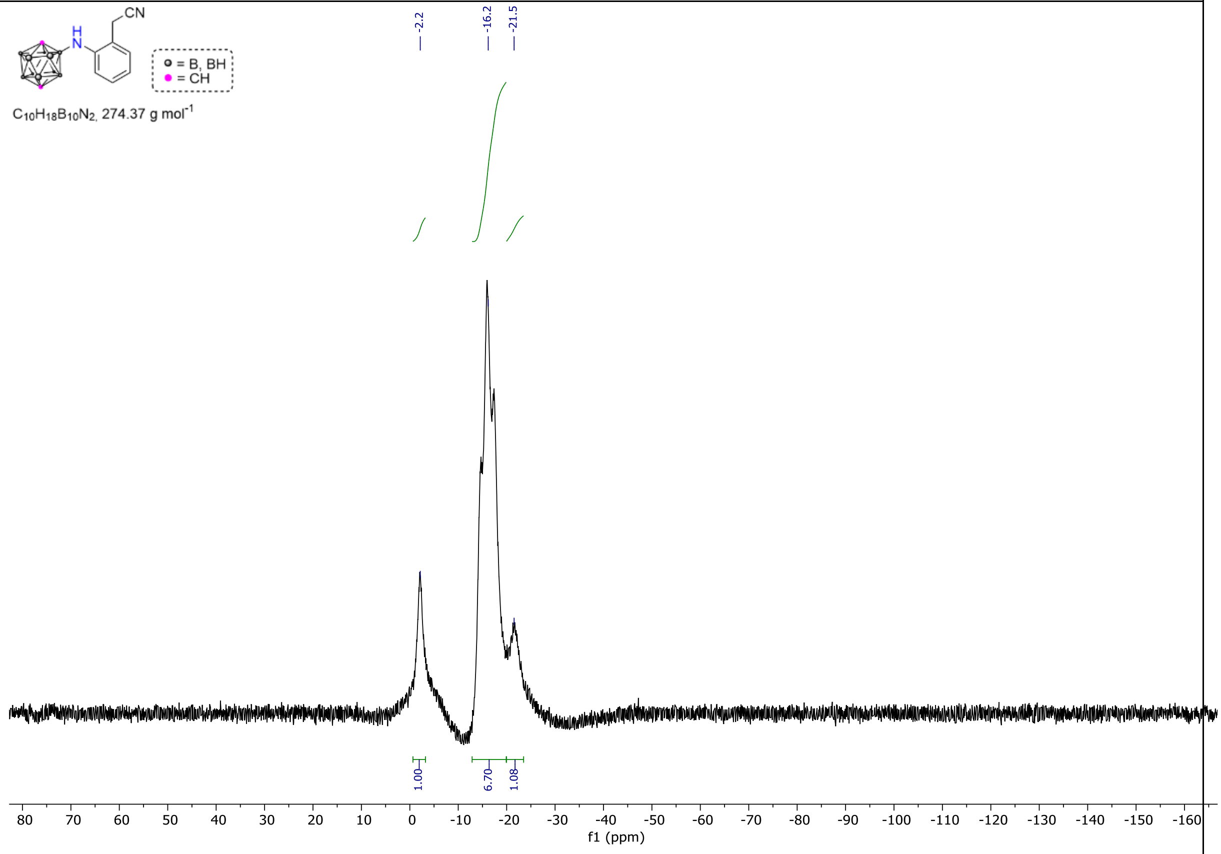

*Figure S 15. ^{11}B{^{1}H} NMR spectrum of **p1** in DMSO-d_6.*

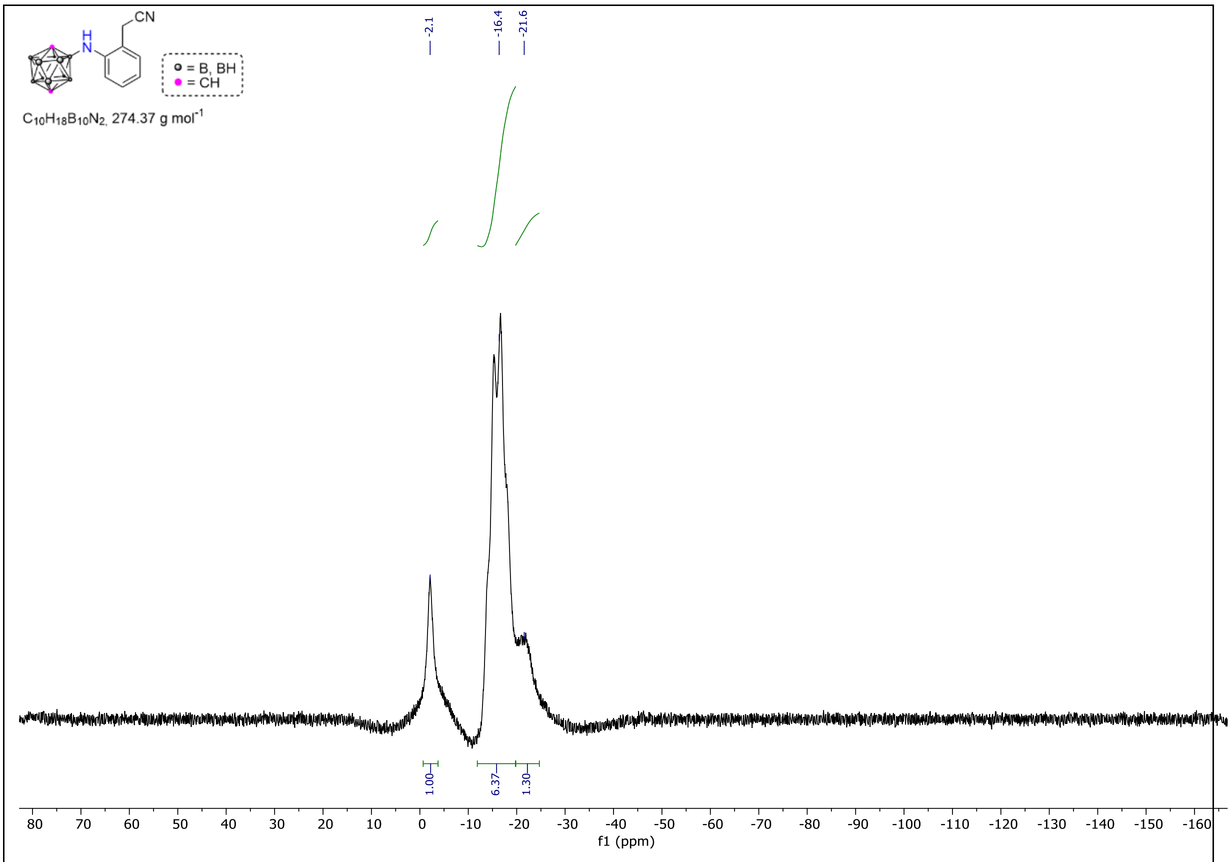

*Figure S 16. ^{11}B NMR spectrum of **p1** in DMSO-d$_6$.*

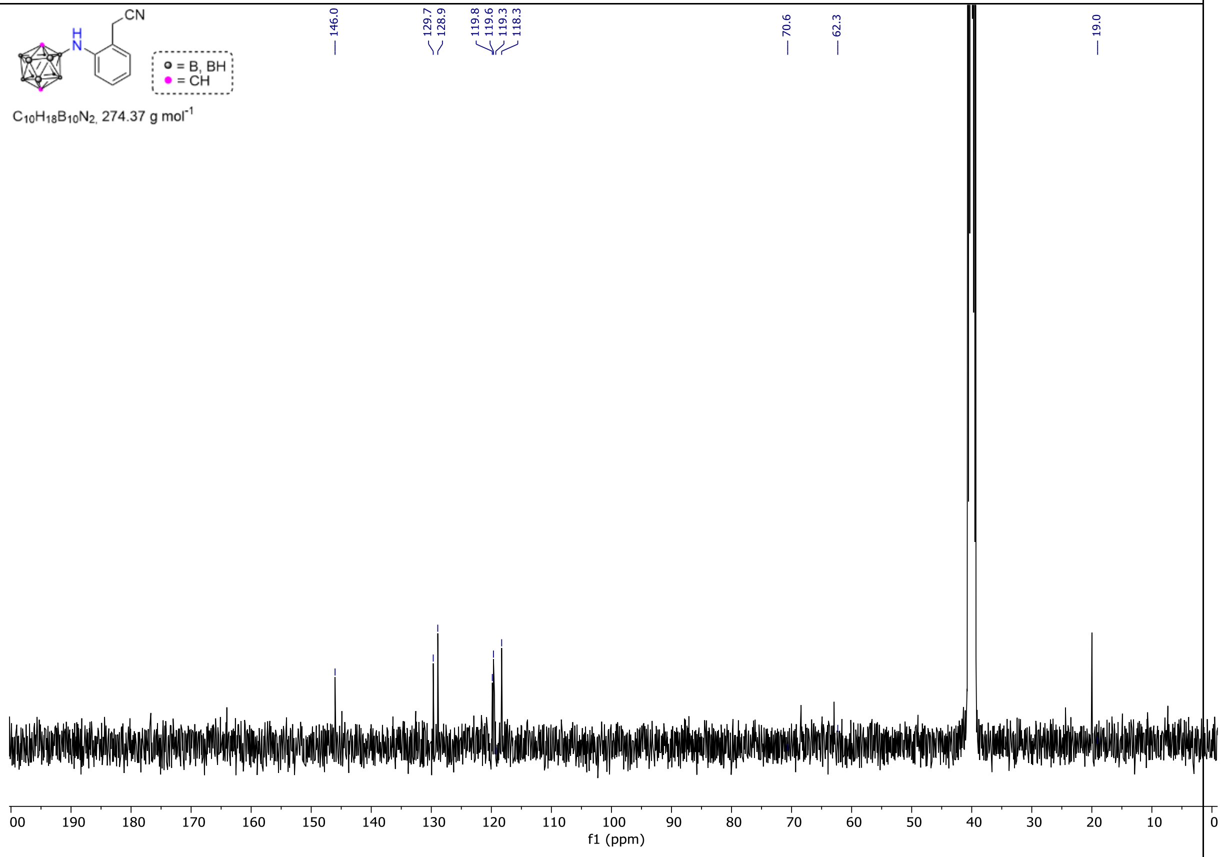

*Figure S 17. ^{13}C{^{1}H} NMR spectrum of **p1** in DMSO-d$_6$.*

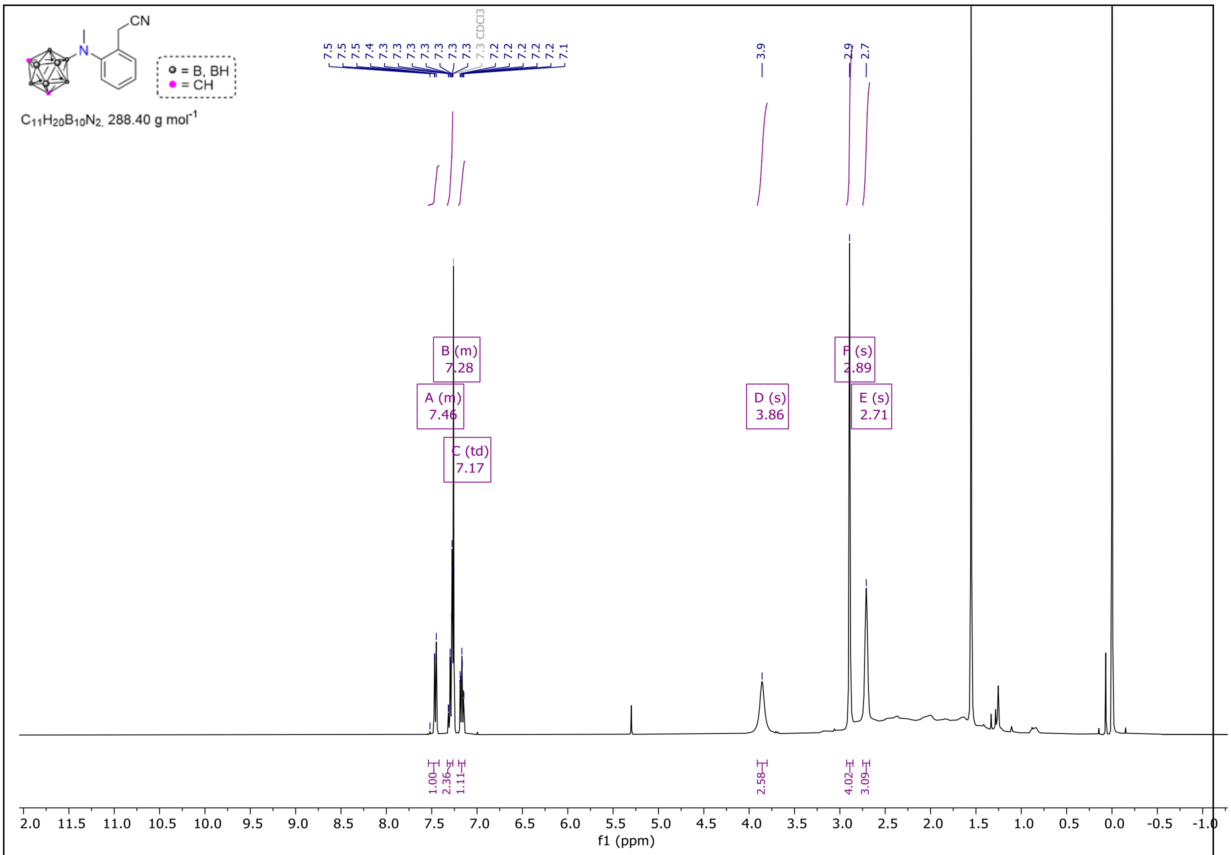

Figure S 18. [1]*H NMR spectrum of* ***m2*** *in* $CDCl_3$.

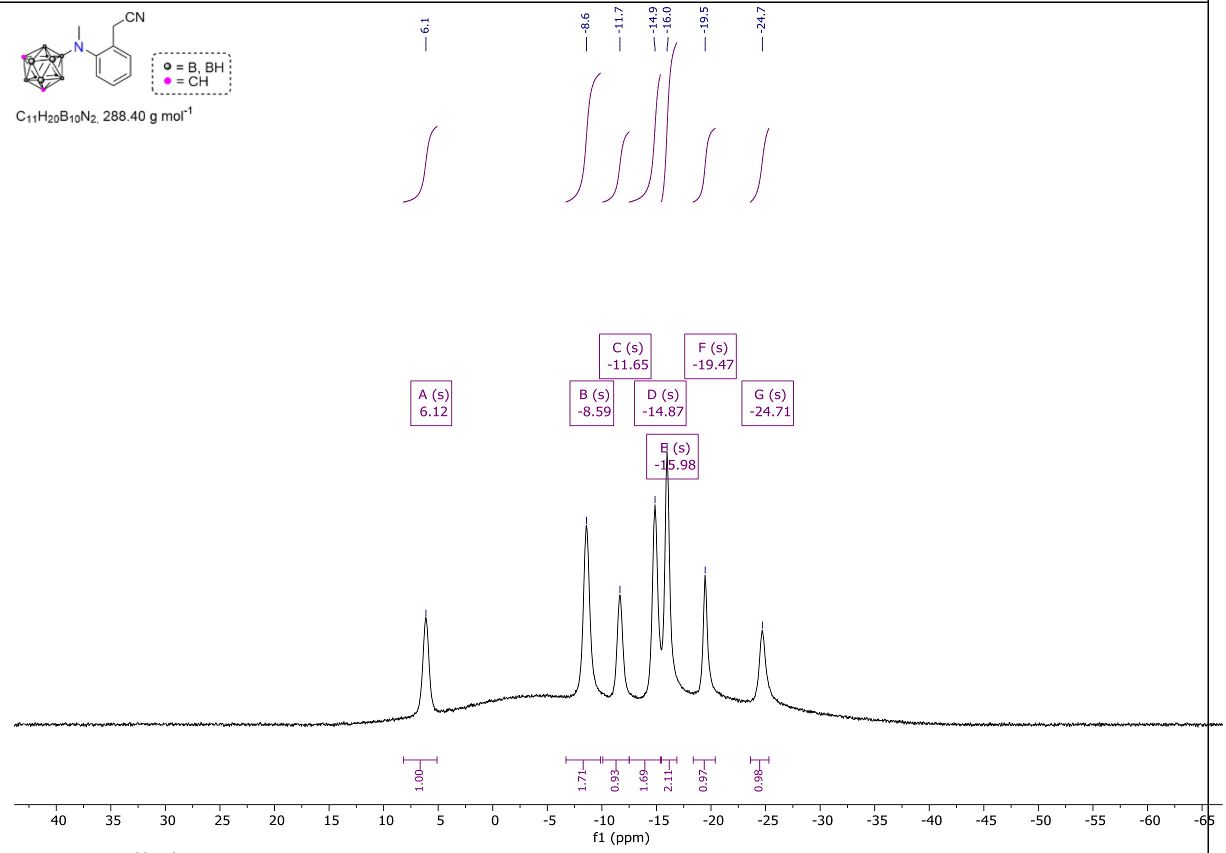

Figure S 19. [11]*B{*[1]*H} NMR spectrum of* ***m2*** *in* $CDCl_3$.

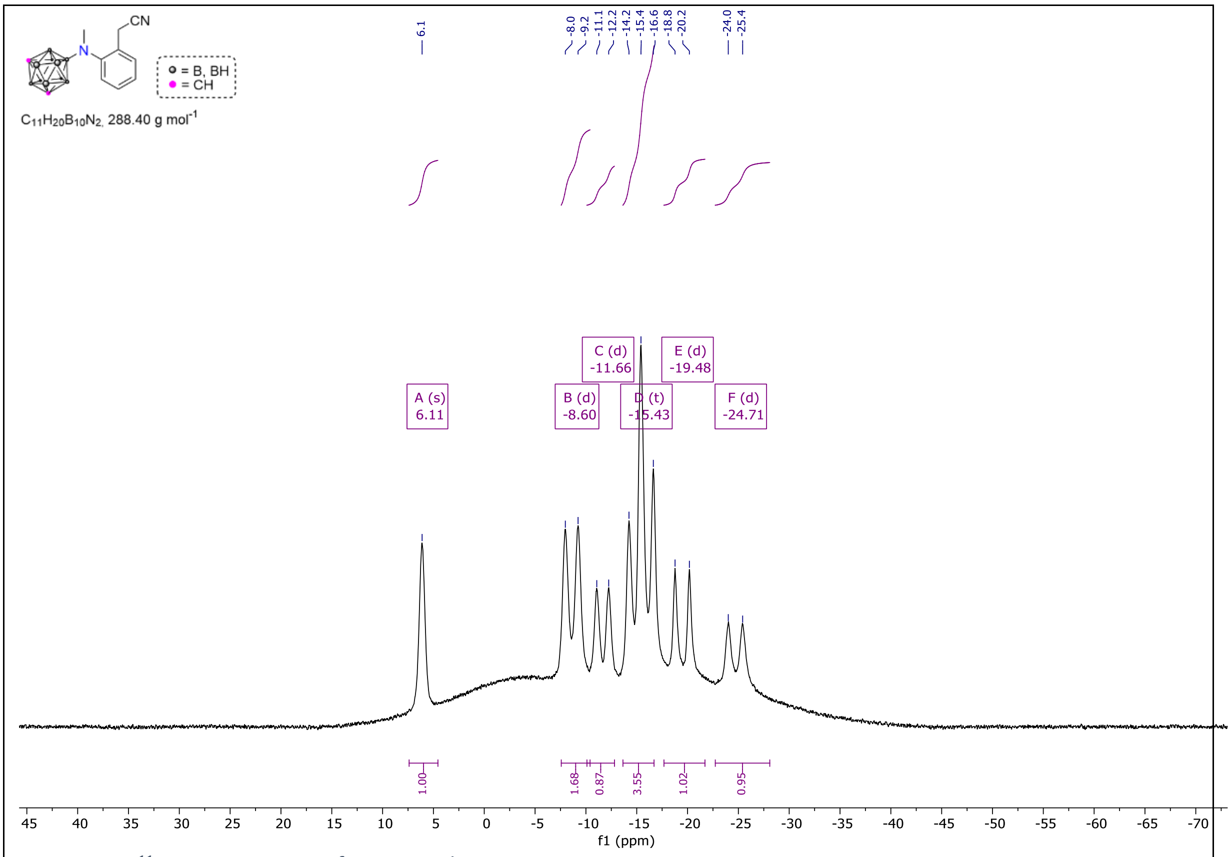

*Figure S 20. ^{11}B NMR spectrum of **m2** in $CDCl_3$.*

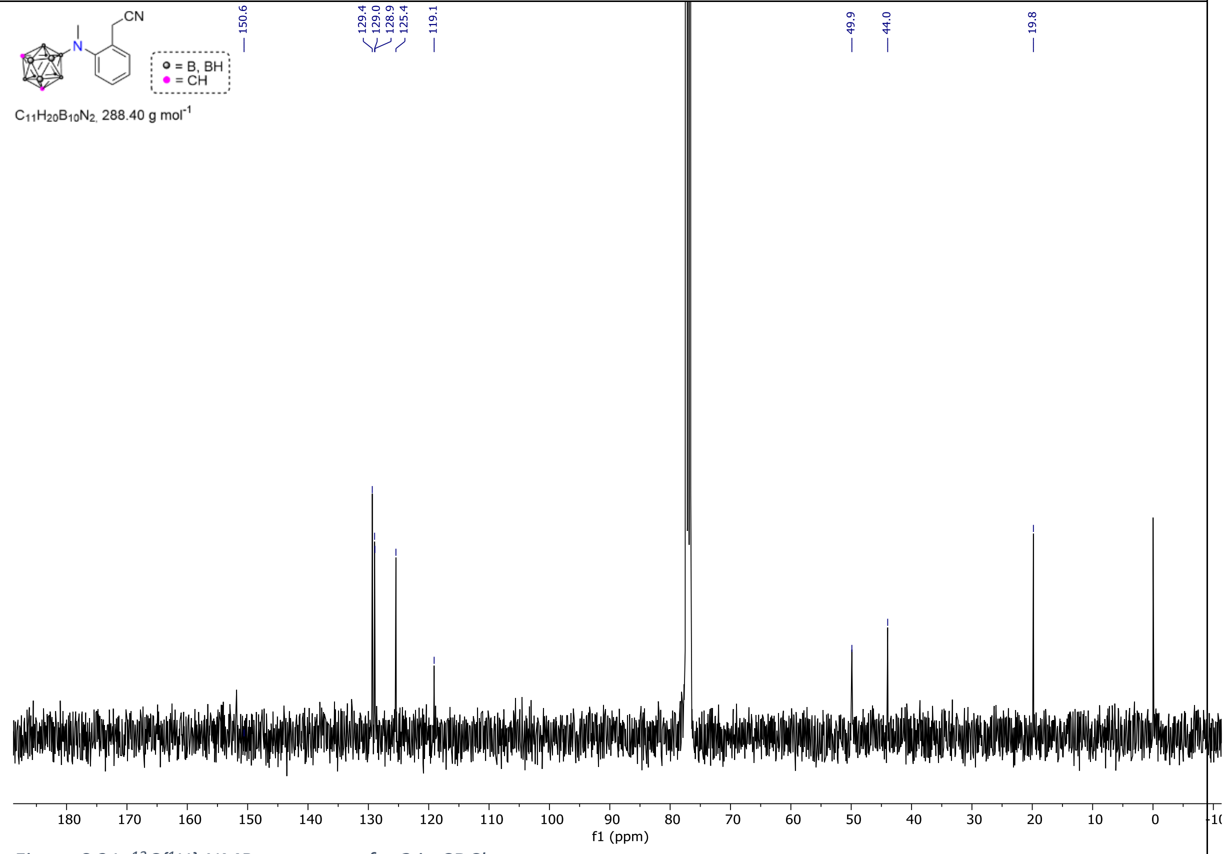

*Figure S 21. $^{13}C\{^1H\}$ NMR spectrum of **m2** in $CDCl_3$.*

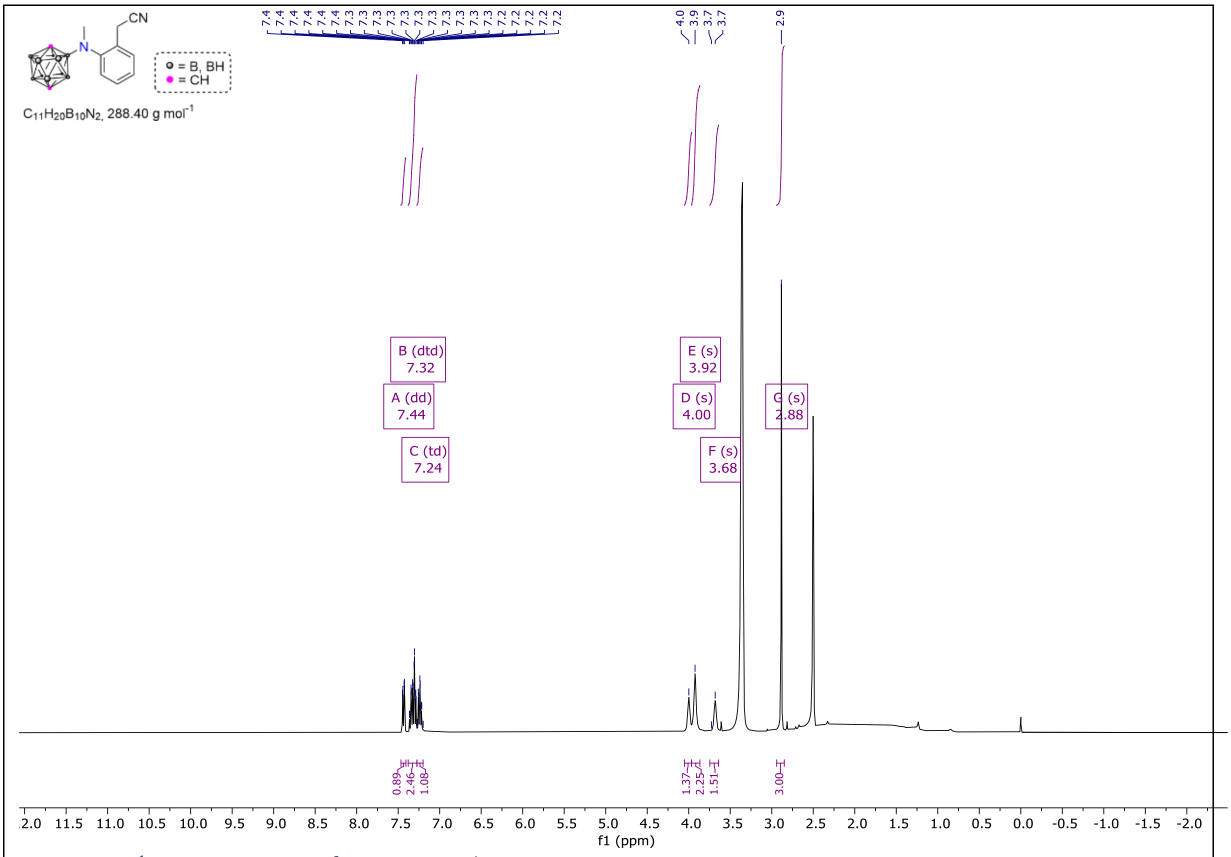

*Figure S 22. ^{1}H NMR spectrum of **p2** in DMSO-d_6.*

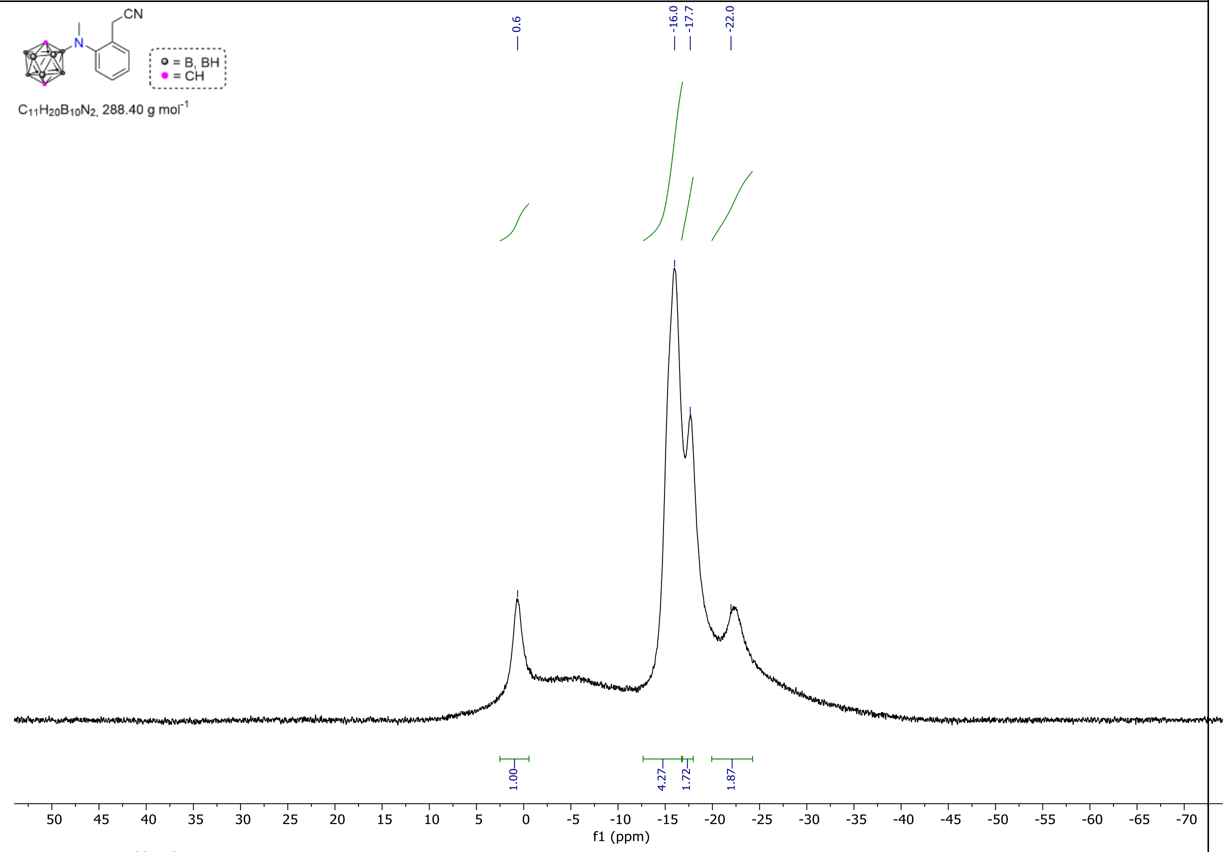

*Figure S 23. ^{11}B{^{1}H} NMR spectrum of **p2** in DMSO-d_6.*

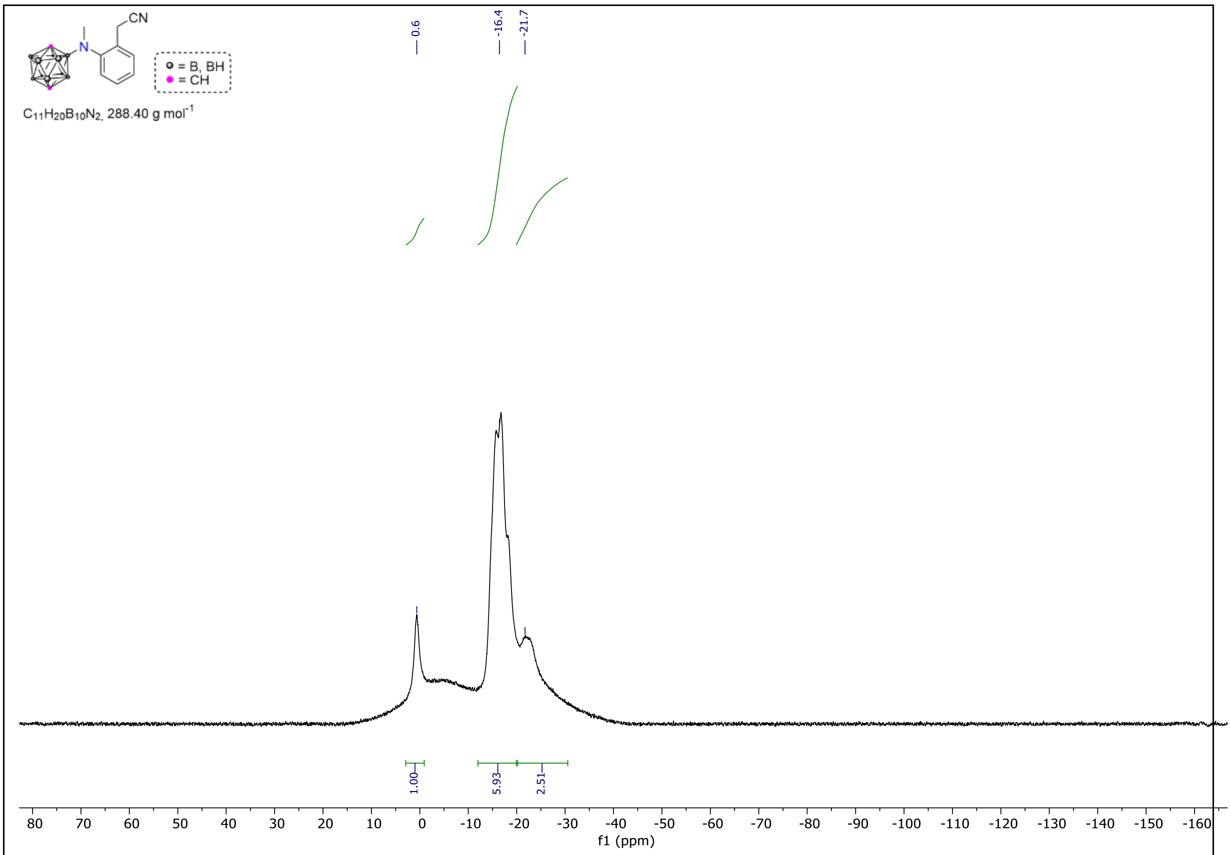

Figure S 24. ^{11}*B NMR spectrum of* ***p2*** *in DMSO-d*$_6$*.*

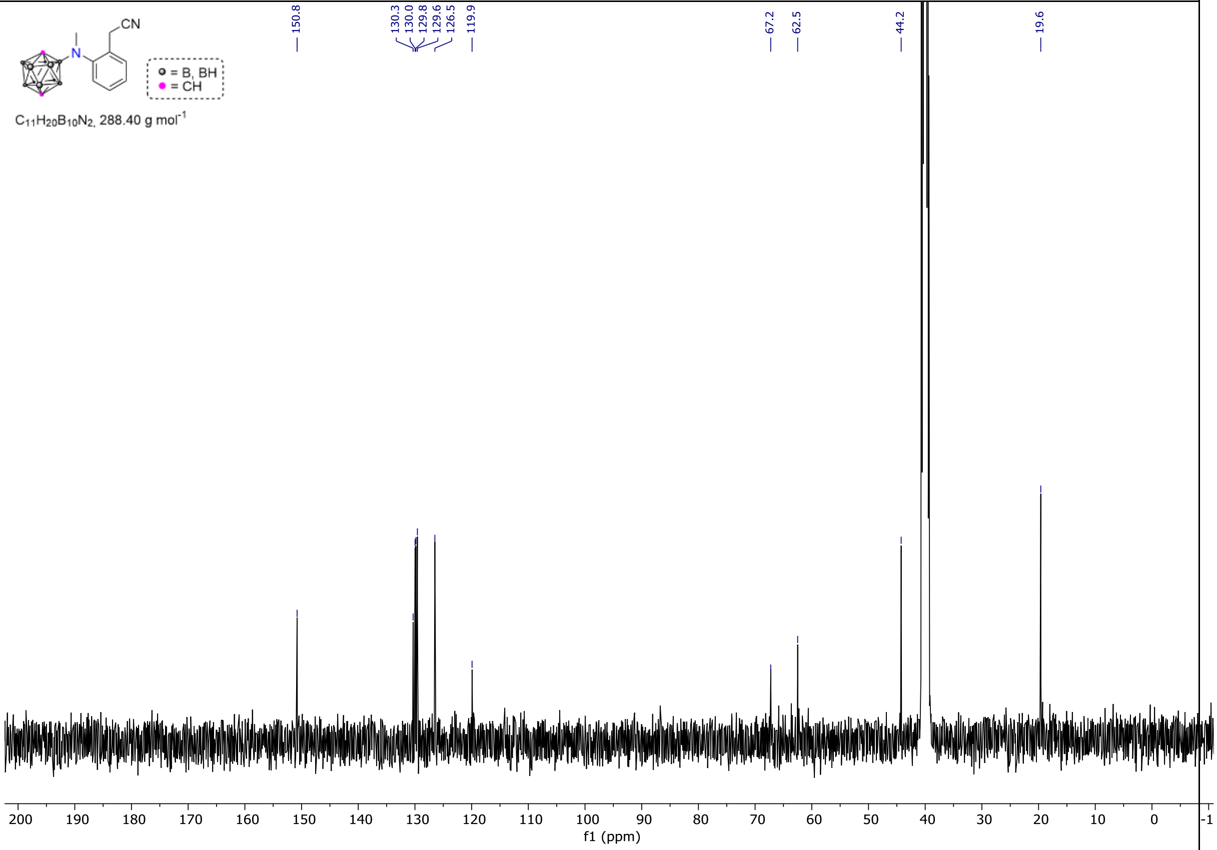

Figure S 25. ^{13}*C NMR spectrum of* ***p2*** *in DMSO-d*$_6$*.*

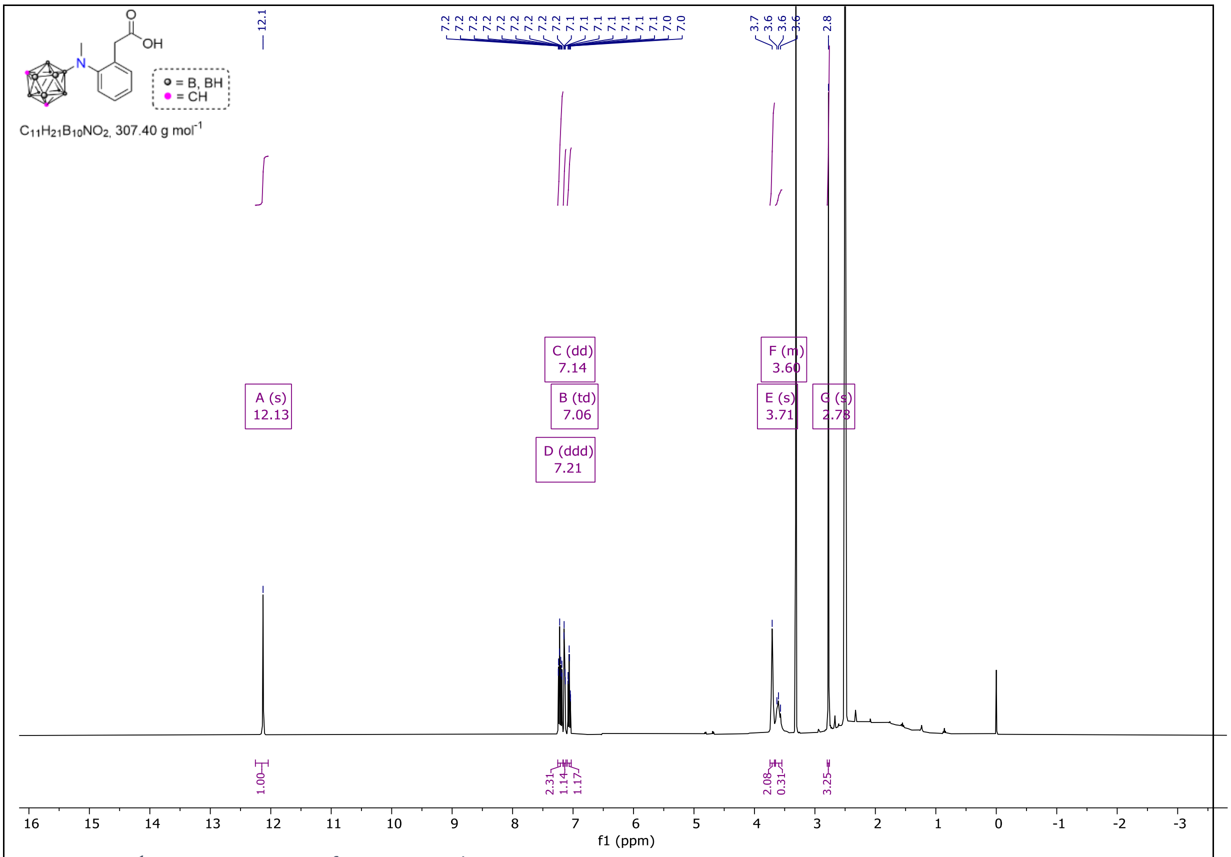

*Figure S 26. ^{1}H NMR spectrum of **3** in DMSO-d_6.*

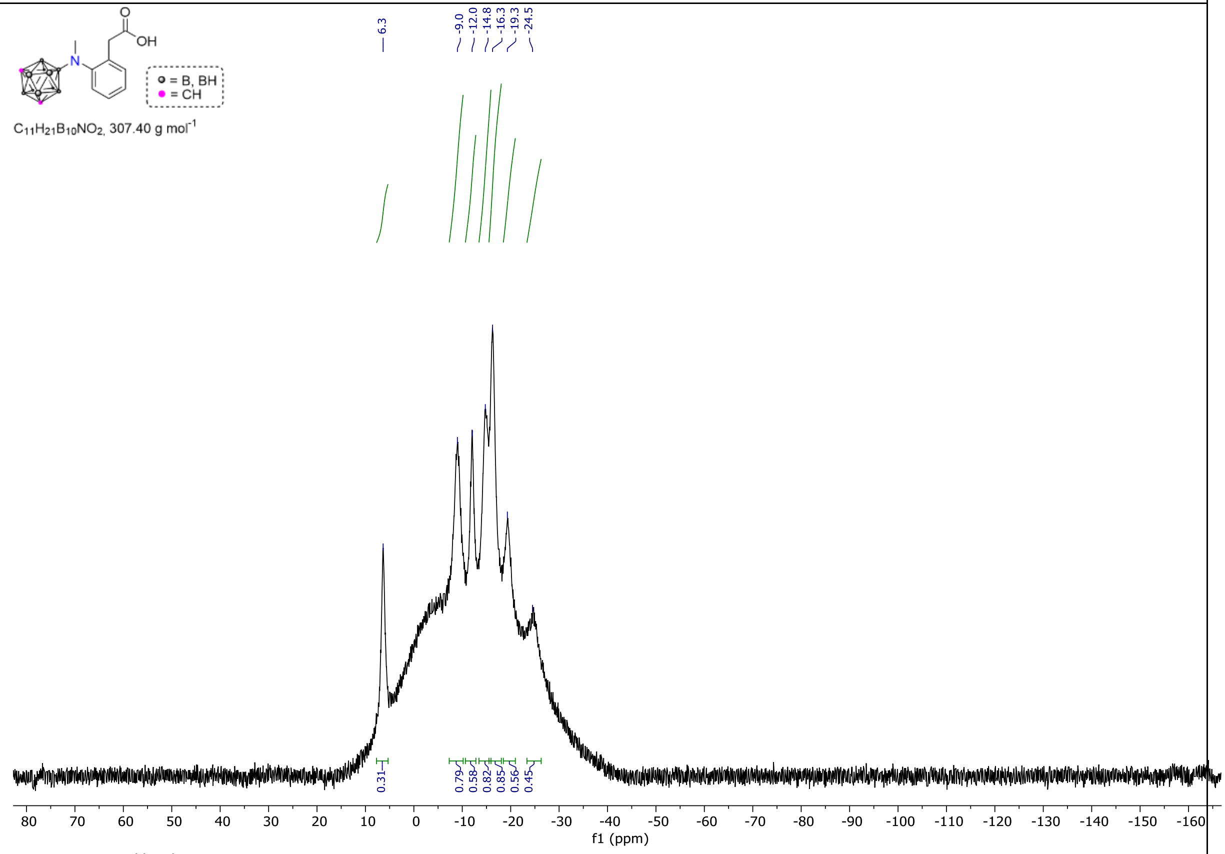

*Figure S 27. $^{11}B\{^{1}H\}$ NMR spectrum of **3** in DMSO-d_6.*

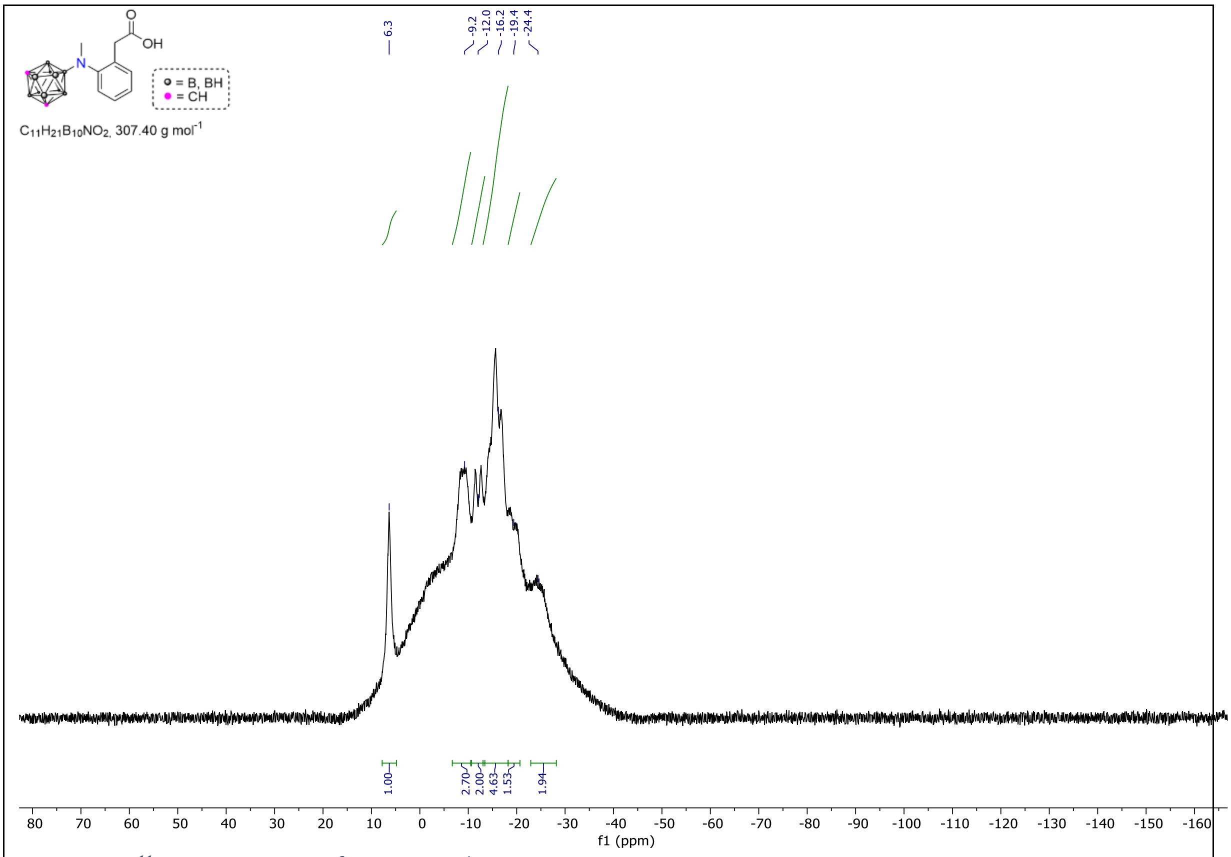

*Figure S 28. ^{11}B NMR spectrum of **3** in DMSO-d$_6$.*

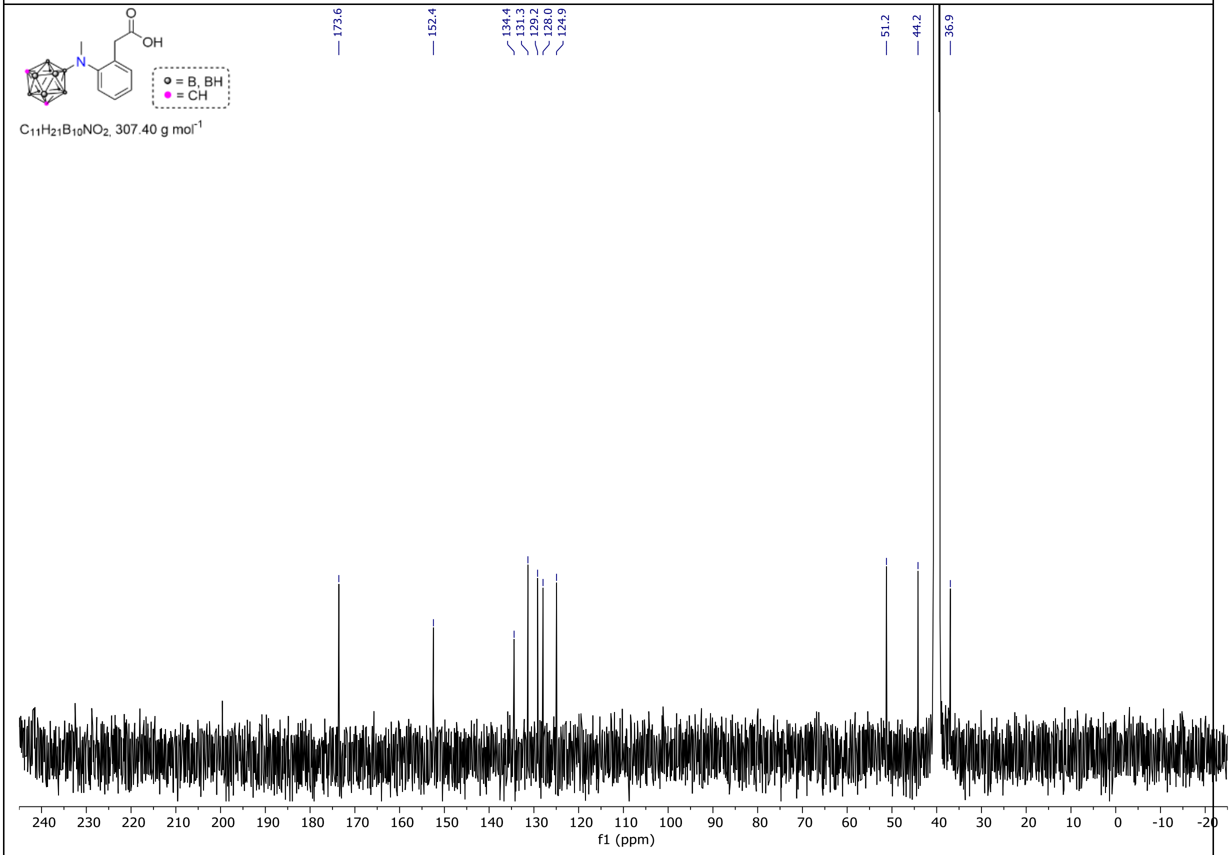

*Figure S 29. ^{13}C NMR spectrum of **3** in DMSO-d$_6$.*

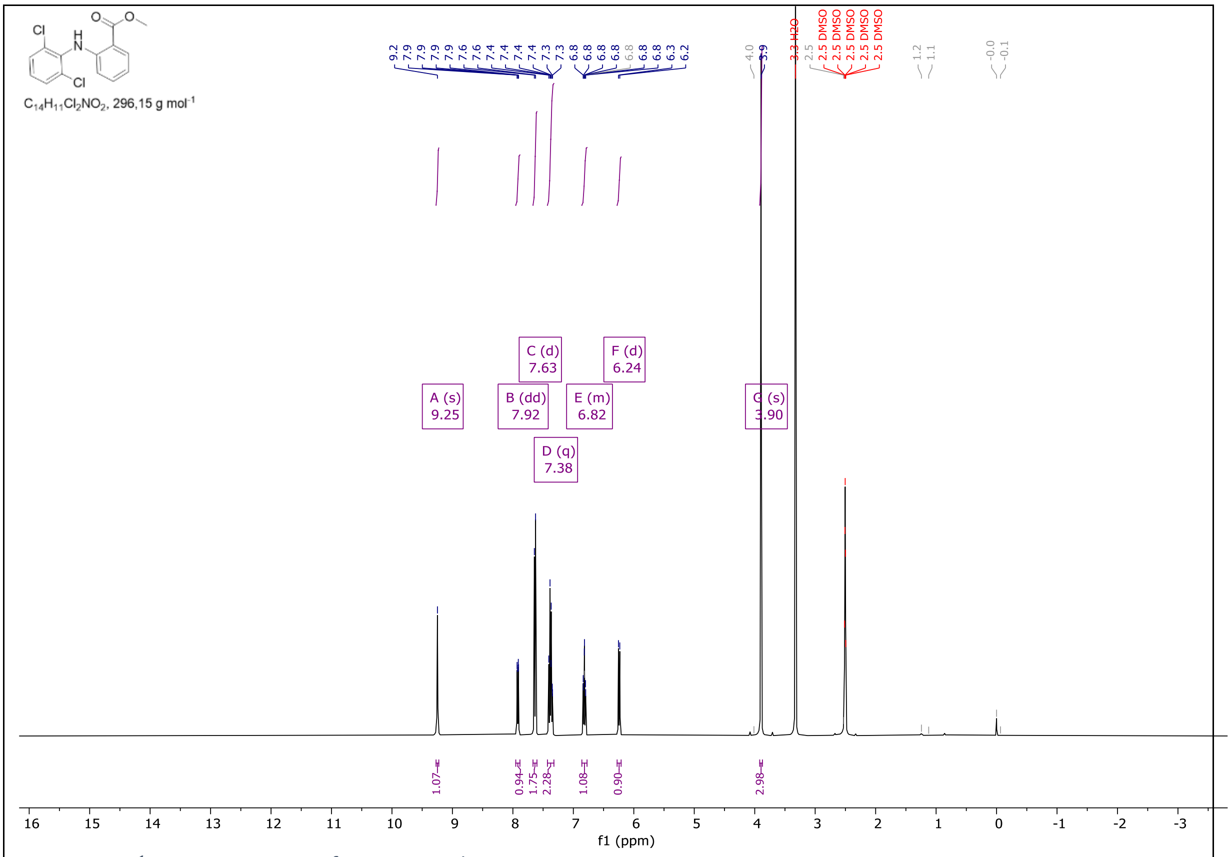

Figure S 30. ^{1}H NMR spectrum of **8** in DMSO-d_6.

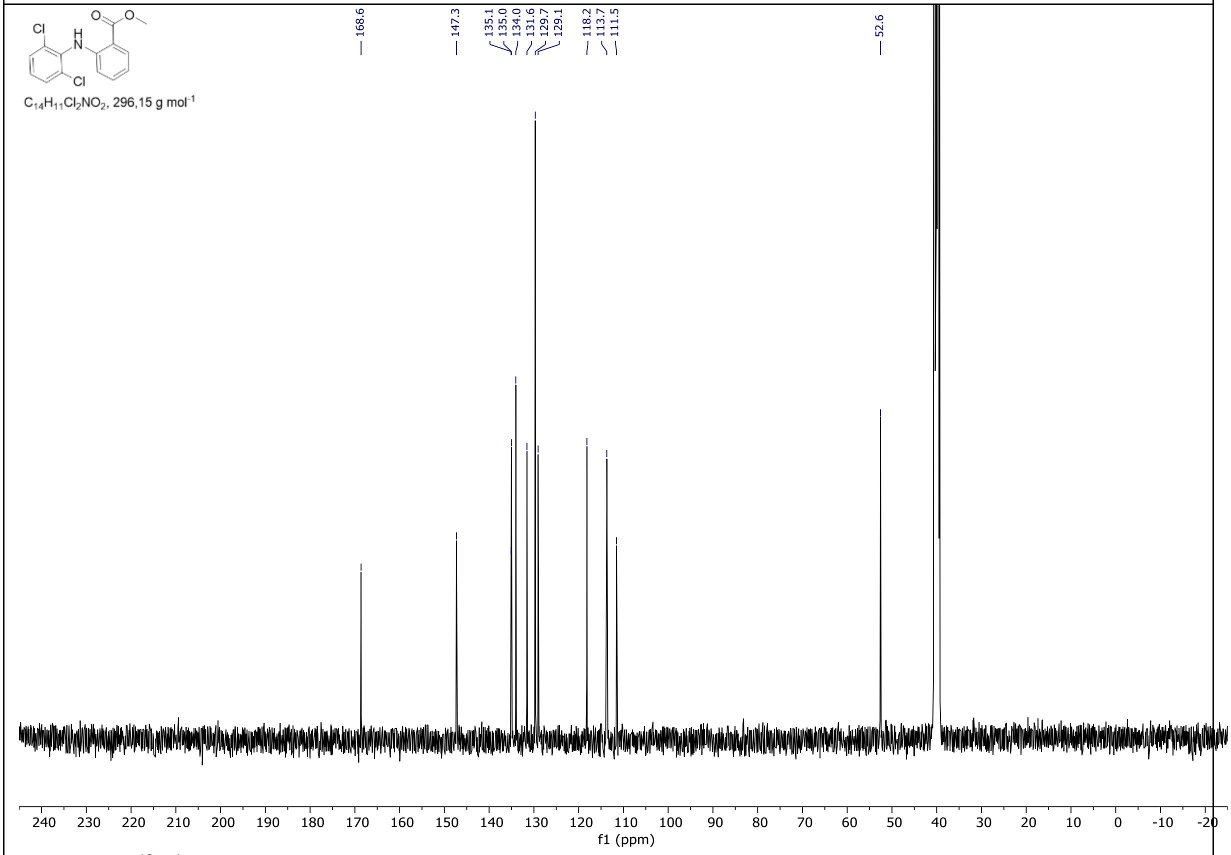

Figure S 31. $^{13}C\{^{1}H\}$ NMR spectrum of **8** in DMSO-d_6.

5 HPLC Data for compounds *m*1, *p*1, *m*2, *p*2, 3, 7, 8 and 11

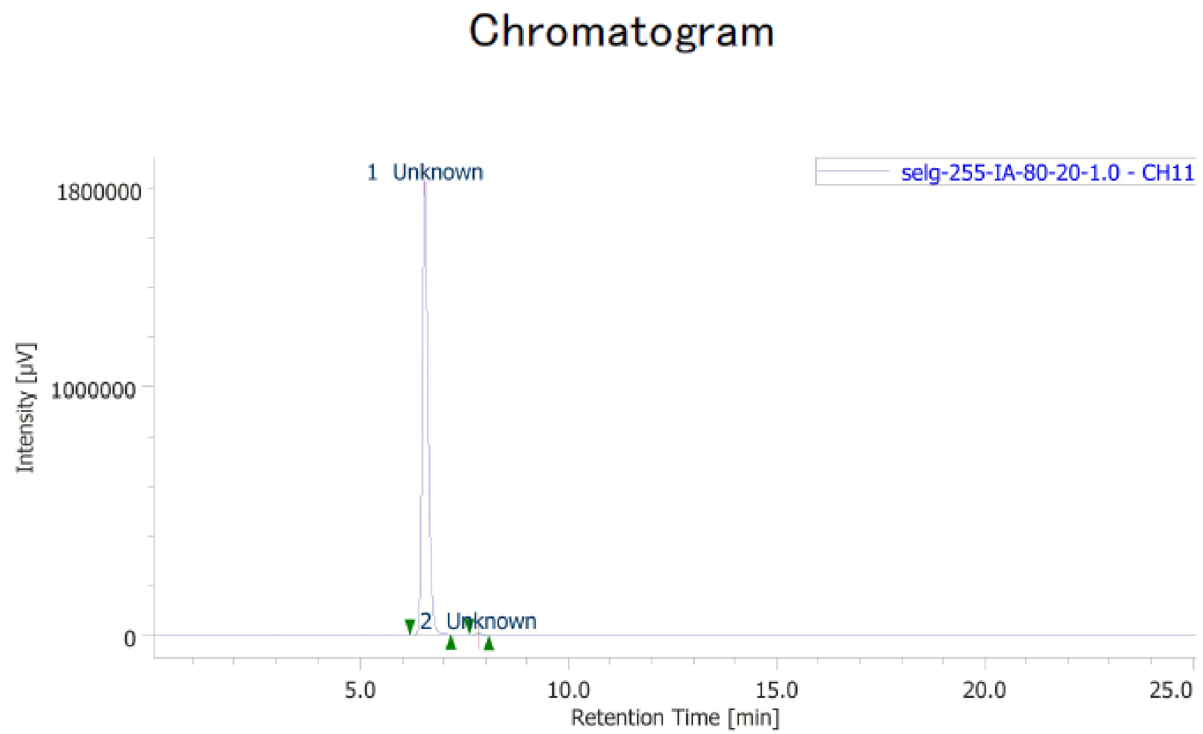

Chromatogram Information	
User Name	User
Date Modified	19.07.2023 07:49:00
Description	
HPLC System Name	HPLC (Melania)
Injection Date	19.07.2023 01:47:21
Volume	20.0 [μL]
Sample #	13
Project Name	Jun2023
Acquisition Time	30.0 [min]
Acquisition Sequence	18.07.2023 Tag2
Control Method	IA-30min-80A-20B-0C-0D-1.0
Peak ID Table	
Calibration Method	
Additional Information	

Channel & Peak Information Table	
Chromatogram Name	selg-255-IA-80-20-1.0-CH11
Sample Name	
Channel Name	255,0nm
Sampling Interval	200 [msec]
Peak Method	(Manual)

#	Peak Name	CH	tR [min]	Area [μV·sec]	Height [μV]	Area%	Height%	Quantity	NTP	Resolution	Symmetry Factor	Warning
1	Unknown	11	6.537	17145789	1830817	99.745	99.823	N/A	12361	4.276	1.295	
2	Unknown	11	7.830	43863	3248	0.255	0.177	N/A	7114	N/A	1.063	

Figure S 32. HPLC chromatogram of ***m1****.*

Chromatogram

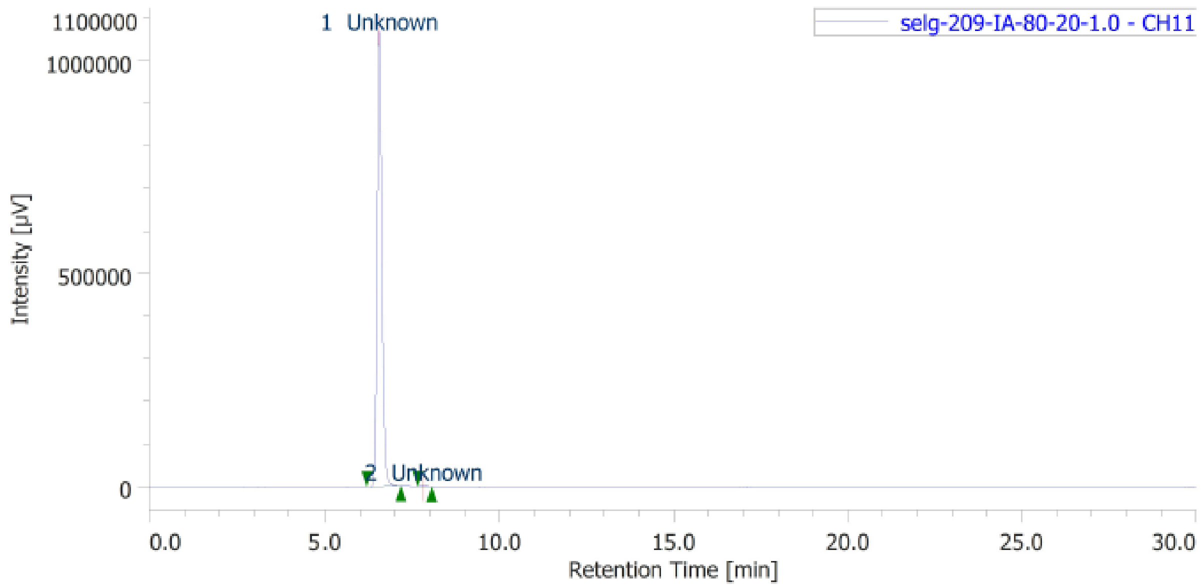

Chromatogram Information	
User Name	User
Date Modified	19.07.2023 07:41:37
Description	
HPLC System Name	HPLC (Melania)
Injection Date	19.07.2023 00:16:33
Volume	20.0 [µL]
Sample #	11
Project Name	Jun2023
Acquisition Time	30.0 [min]
Acquisition Sequence	18.07.2023 Tag2
Control Method	IA-30min-80A-20B-0C-0D-1.0
Peak ID Table	
Calibration Method	
Additional Information	

Channel & Peak Information Table	
Chromatogram Name	selg-209-IA-80-20-1.0-CH11
Sample Name	
Channel Name	256,0nm
Sampling Interval	200 [msec]
Peak Method	(Manual)

#	Peak Name	CH	tR [min]	Area [µV·sec]	Height [µV]	Area%	Height%	Quantity	NTP	Resolution	Symmetry Factor	Warning
1	Unknown	11	6.537	10152037	1070499	99.849	99.887	N/A	11814	4.325	1.260	
2	Unknown	11	7.817	15355	1214	0.151	0.113	N/A	7849	N/A	1.150	

*Figure S 33. HPLC chromatogram of **p1**.*

Chromatogram

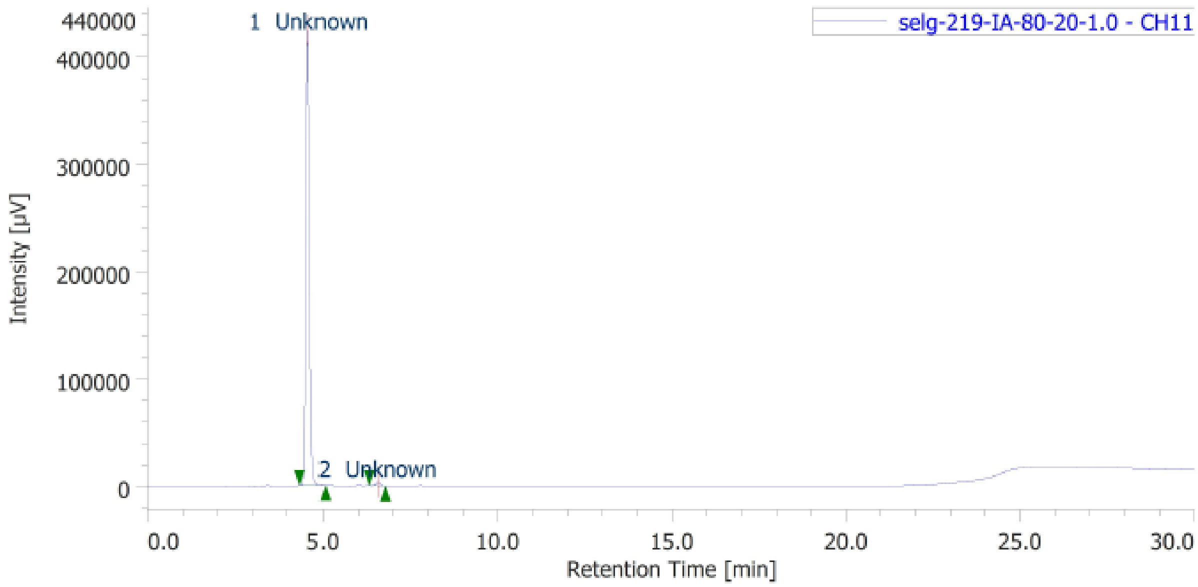

Chromatogram Information	
User Name	User
Date Modified	19.07.2023 07:39:39
Description	
HPLC System Name	HPLC (Melania)
Injection Date	18.07.2023 23:31:10
Volume	20.0 [µL]
Sample #	10
Project Name	Jun2023
Acquisition Time	30.0 [min]
Acquisition Sequence	18.07.2023 Tag2
Control Method	IA-30min-80A-20B-0C-0D-1.0
Peak ID Table	
Calibration Method	
Additional Information	

Channel & Peak Information Table	
Chromatogram Name	selg-219-IA-80-20-1.0-CH11
Sample Name	
Channel Name	270,0nm
Sampling Interval	200 [msec]
Peak Method	(Manual)

#	Peak Name	CH	tR [min]	Area [µV·sec]	Height [µV]	Area%	Height%	Quantity	NTP	Resolution	Symmetry Factor	Warning
1	Unknown	11	4.580	2917992	423745	98.984	99.202	N/A	10775	9.586	1.165	
2	Unknown	11	6.547	29946	3408	1.016	0.798	N/A	12427	N/A	1.195	

Figure S 34. HPLC chromatogram of ***m2****.*

Chromatogram

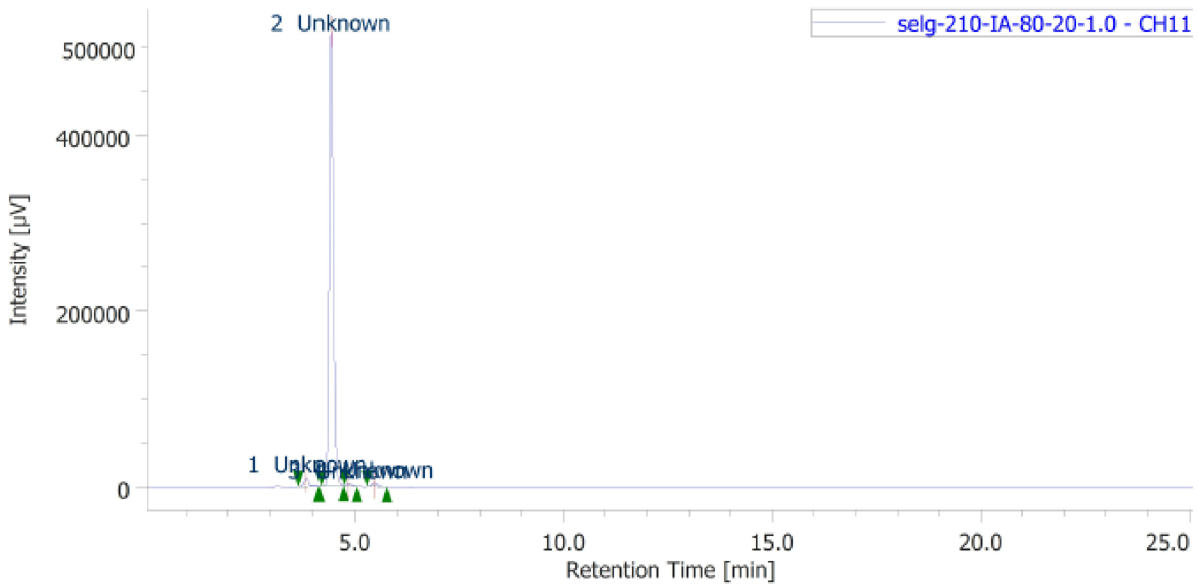

Chromatogram Information	
User Name	User
Date Modified	19.07.2023 07:55:47
Description	
HPLC System Name	HPLC (Melania)
Injection Date	19.07.2023 04:48:55
Volume	20.0 [µL]
Sample #	17
Project Name	Jun2023
Acquisition Time	30.0 [min]
Acquisition Sequence	18.07.2023 Tag2
Control Method	IA-30min-80A-20B-0C-0D-1.0
Peak ID Table	
Calibration Method	
Additional Information	

Channel & Peak Information Table	
Chromatogram Name	selg-210-IA-80-20-1.0-CH11
Sample Name	
Channel Name	258,0nm
Sampling Interval	200 [msec]
Peak Method	(Manual)

#	Peak Name	CH	tR [min]	Area [µV·sec]	Height [µV]	Area%	Height%	Quantity	NTP	Resolution	Symmetry Factor	Warning
1	Unknown	11	3.873	70724	10182	1.980	1.912	N/A	8205	3.264	0.970	
2	Unknown	11	4.430	3450872	516258	96.591	96.955	N/A	10741	2.279	1.185	
3	Unknown	11	4.850	15583	2109	0.436	0.396	N/A	9549	2.848	1.296	
4	Unknown	11	5.467	35488	3922	0.993	0.737	N/A	8604	N/A	1.193	

*Figure S 35. HPLC chromatogram of **p2**.*

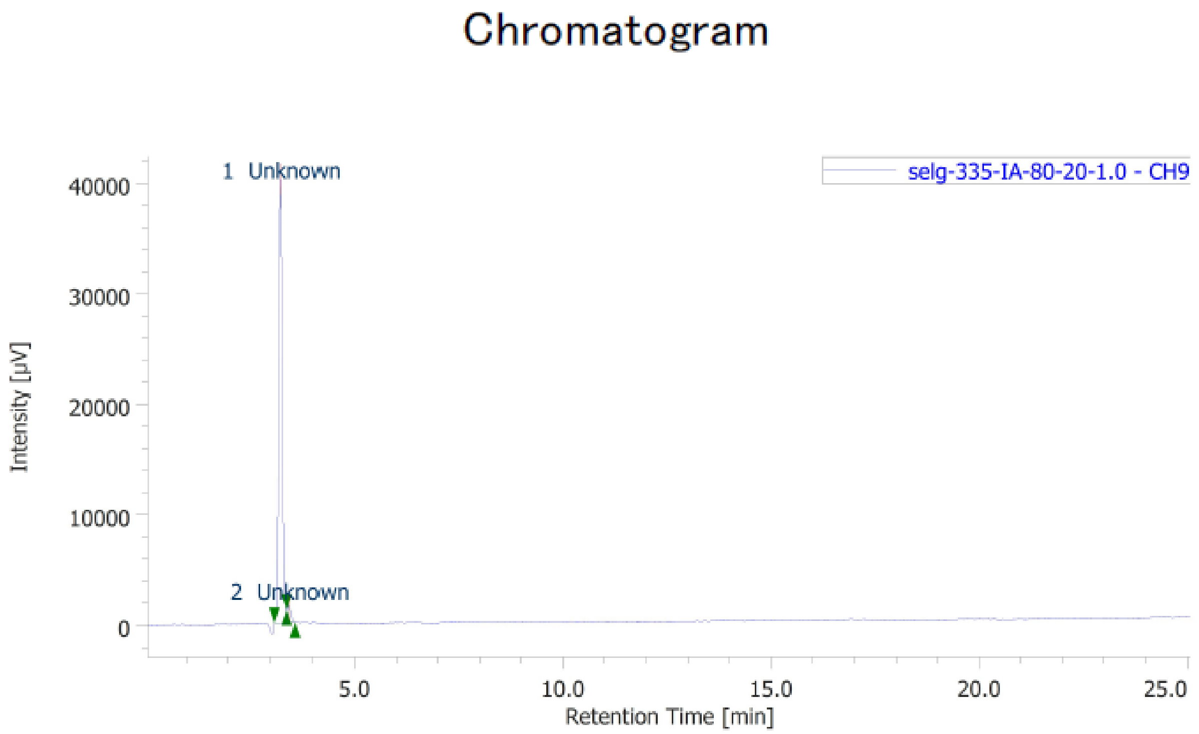

Chromatogram Information

User Name	User
Date Modified	19.07.2023 08:06:42
Description	
HPLC System Name	HPLC (Melania)
Injection Date	19.07.2023 05:34:19
Volume	20.0 [µL]
Sample #	18
Project Name	Jun2023
Acquisition Time	30.0 [min]
Acquisition Sequence	18.07.2023 Tag2
Control Method	IA-30min-80A-20B-0C-0D-1.0
Peak ID Table	
Calibration Method	
Additional Information	

Channel & Peak Information Table

Chromatogram Name	selg-335-IA-80-20-1.0-CH9
Sample Name	
Channel Name	MaxABS(210.0-250.0)
Sampling Interval	200 [msec]
Peak Method	(Manual)

#	Peak Name	CH	tR [min]	Area [µV·sec]	Height [µV]	Area%	Height%	Quantity	NTP	Resolution	Symmetry Factor	Warning
1	Unknown	9	3.253	232894	40160	95.884	96.105	N/A	7610	N/A	1.139	
2	Unknown	9	3.460	9997	1628	4.116	3.895	N/A	N/A	N/A	N/A	

Figure S 36. HPLC chromatogram of ***3****.*

Chromatogram

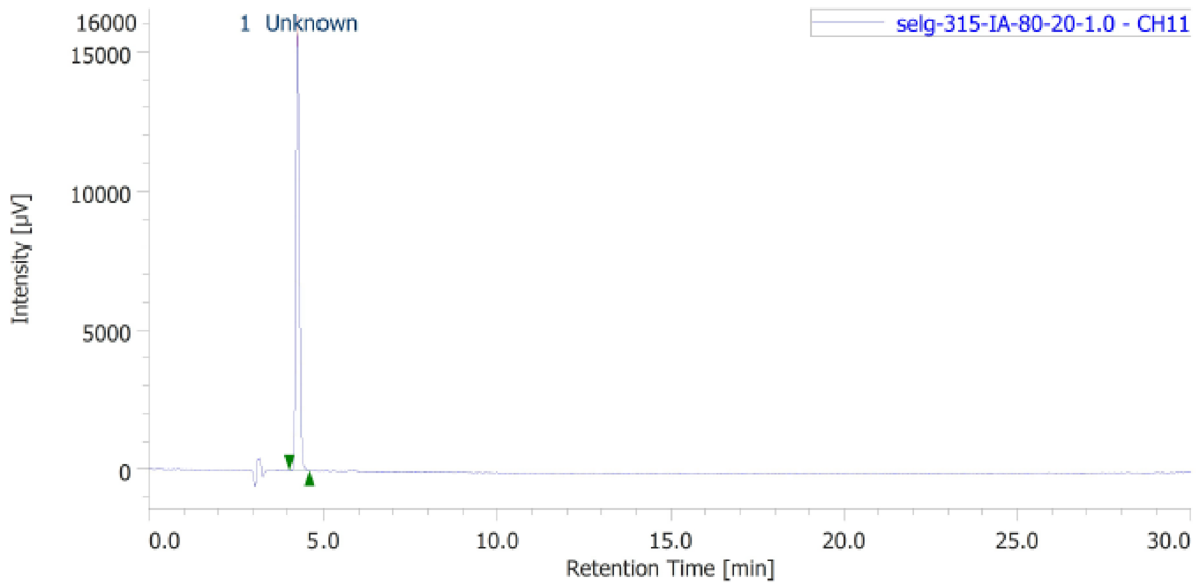

Chromatogram Information	
User Name	User
Date Modified	19.07.2023 07:32:12
Description	
HPLC System Name	HPLC (Melania)
Injection Date	18.07.2023 21:14:59
Volume	20.0 [µL]
Sample #	19
Project Name	Jun2023
Acquisition Time	30.0 [min]
Acquisition Sequence	18.07.2023 Tag2
Control Method	IA-30min-80A-20B-0C-0D-1.0
Peak ID Table	
Calibration Method	
Additional Information	

Channel & Peak Information Table	
Chromatogram Name	selg-315-IA-80-20-1.0-CH11
Sample Name	
Channel Name	338,0nm
Sampling Interval	200 [msec]
Peak Method	(Manual)

#	Peak Name	CH	tR [min]	Area [µV·sec]	Height [µV]	Area%	Height%	Quantity	NTP	Resolution	Symmetry Factor	Warning
1	Unknown	11	4.283	105230	15785	100.000	100.000	N/A	9825	N/A	1.181	

*Figure S 37. HPLC chromatogram of **7**.*

Chromatogram

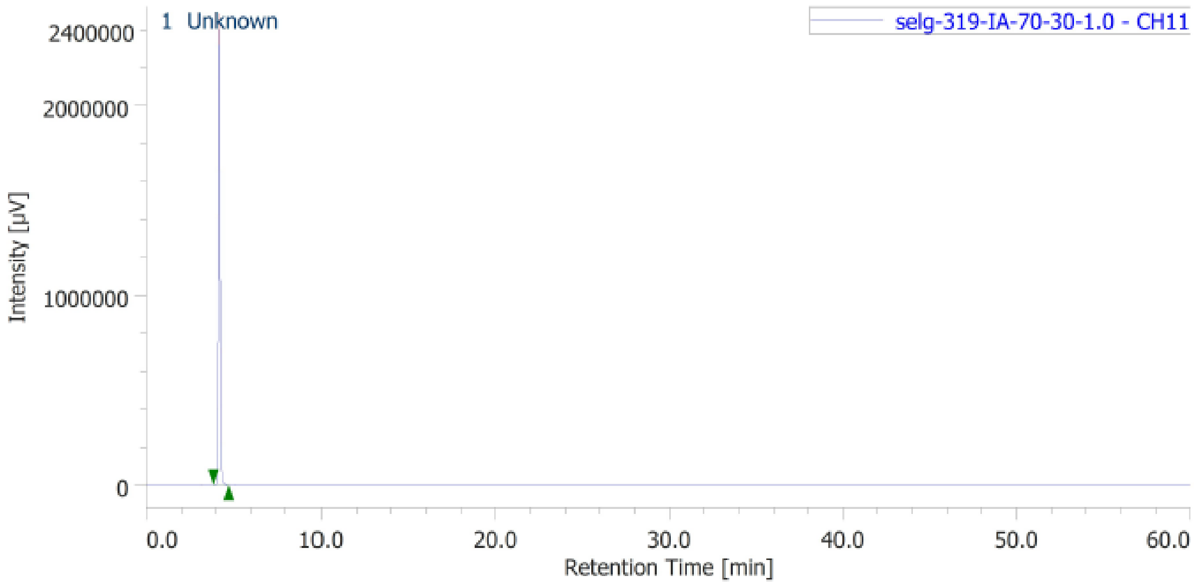

Chromatogram Information	
User Name	User
Date Modified	18.07.2023 10:10:41
Description	
HPLC System Name	HPLC (Melania)
Injection Date	17.07.2023 17:33:43
Volume	20.0 [µL]
Sample #	20
Project Name	Jun2023
Acquisition Time	60.0 [min]
Acquisition Sequence	13.07.2023 Tag1
Control Method	IA-60min-70A-30B-0C-0D-1.0
Peak ID Table	
Calibration Method	
Additional Information	

Channel & Peak Information Table	
Chromatogram Name	selg-319-IA-70-30-1.0-CH11
Sample Name	
Channel Name	337.0nm
Sampling Interval	200 [msec]
Peak Method	(Manual)

#	Peak Name	CH	tR [min]	Area [µV·sec]	Height [µV]	Area%	Height%	Quantity	NTP	Resolution	Symmetry Factor	Warning
1	Unknown	11	4.183	15604909	2399785	100.000	100.000	N/A	10843	N/A	1.161	

*Figure S 38. HPLC chromatogram of **8**.*

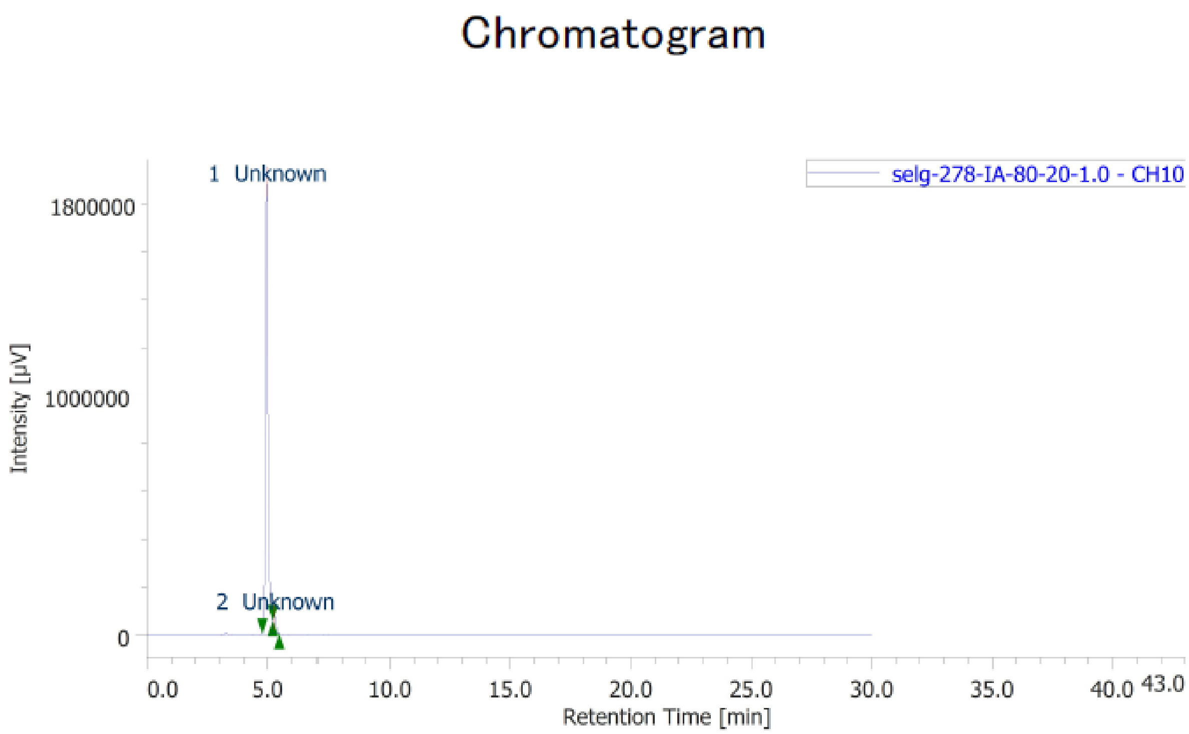

Chromatogram Information	
User Name	User
Date Modified	18.07.2023 15:48:23
Description	
HPLC System Name	HPLC (Melania)
Injection Date	18.07.2023 15:06:33
Volume	20.0 [µL]
Sample #	3
Project Name	Jun2023
Acquisition Time	30.0 [min]
Acquisition Sequence	18.07.2023 Tag2
Control Method	IA-30min-80A-20B-0C-0D-1.0
Peak ID Table	
Calibration Method	
Additional Information	

Channel & Peak Information Table	
Chromatogram Name	selg-278-IA-80-20-1.0-CH10
Sample Name	
Channel Name	230.0nm
Sampling Interval	200 [msec]
Peak Method	(Manual)

#	Peak Name	CH	tR [min]	Area [µV·sec]	Height [µV]	Area%	Height%	Quantity	NTP	Resolution	Symmetry Factor	Warning
1	Unknown	10	4.943	13704084	1885212	95.497	95.967	N/A	13429	N/A	1.430	
2	Unknown	10	5.270	646193	79219	4.503	4.033	N/A	N/A	N/A	N/A	

*Figure S 39. HPLC chromatogram of **11**.*
